# Supplementary material for: Spatial and single-cell expression analyses reveal complex expression domains in early wheat spike development
Source: Genome Biol. 2025 Oct 13;26:352. doi: 10.1186/s13059-025-03811-3 (PMC12516876; doi:10.1186/s13059-025-03811-3)

SUPPLEMENTAL FIGURES

TABLE OF CONTENTS

SPATIAL EXPRESSION AND CELL SEGMENTATION
Additional file 1: Fig. S1 Page 3
Additional file 1: Fig. S2 Page 4
Additional file 1: Fig. S3 Page 5
Additional file 1: Fig. S4 Page 6
Additional file 1: Fig. S5 Page 7
Additional file 1: Fig. S6 Page 10
Additional file 1: Fig. S7 Page 11
The basal region below the spike
Additional file 1: Fig. S8 Page 12
Additional file 1: Fig. S9 Page 13
Additional file 1: Fig. S10 Page 14
The transition zone between leaf and spikelet meristems
Additional file 1: Fig. S11 Page 15
The central region of the spike
Additional file 1: Fig. S12 Page 16
Additional file 1: Fig. S13 Page 18
The region proximal to the spikelet meristem (SM) and *FZP* validation
Additional file 1: Fig. S14 Page 19
Additional file 1: Fig. S15 Page 20
Additional file 1: Fig. S16 Page 21
Additional file 1: Fig. S17 Page 22
Spikelet meristem (SM) and floret meristem (FM) regions
Additional file 1: Fig. S18 Page 23
Changes within the inflorescence meristem (IM) and *SPL14* validation
Additional file 1: Fig. S19 Page 24
Additional file 1: Fig. S20 Page 25
Additional file 1: Fig. S21 Page 26
Pseudotime trajectories
Additional file 1: Fig. S22 Page 27

scRNA-SEQ CLUSTERING
Additional file 1: Fig. S23 Page 28
RNA-seq of bottom, middle, and top of spike at W3.0
Additional file 1: Fig. S24 Page 29
Cell cycle enriched clusters
Additional file 1: Fig. S25 Page 30
Epidermal clusters
Additional file 1: Fig. S26 Page 31
Additional file 1: Fig. S27 Page 33
Vascular clusters
Additional file 1: Fig. S28 Page 35

Additional file 1: Fig. S29 Page 38
Central spike clusters
Additional file 1: Fig. S30 Page 39
Additional file 1: Fig. S31 Page 41
Additional file 1: Fig. S32 Page 42
Transition zone between leaves and spike
Additional file 1: Fig. S33 Page 43
Suppressed bract cluster
Additional file 1: Fig. S34 Page 45
Boundary clusters
Additional file 1: Fig. S35 Page 46
Additional file 1: Fig. S36 Page 47
Additional file 1: Fig. S37 Page 48
Cortex cells below developing spikelets
Additional file 1: Fig. S38 Page 49
Additional file 1: Fig. S39 Page 51
Meristem clusters
Additional file 1: Fig. S40 Page 52
Additional file 1: Fig. S41 Page 55
Co-expression analysis
Additional file 1: Fig. S42 Page 56
Additional file 1: Fig. S43 Page 57
scRNA-seq trajectory analysis
Additional file 1: Fig. S44 Page 58

Additional file 1: Fig. S1. Microscopy images of Kronos developing spikes. A, B Stereo microscope images. **C, D** scanning electron microscope images. **A** Shoot apical meristem (SAM) producing leaf primordia. **B** Early stage of spike development showing initial meristem elongation and a lateral meristem before its differentiation into upper and lower ridges. IM= inflorescence meristem (red) and LM= lateral meristem (blue) **C** Double ridge stage (W2.5). SM= spikelet meristem (yellow) and repressed lower ridge (also known as leaf-ridge, violet). **D** Floret primordia stage (W3.5). Spikelet primordia are formed in a distichous organization. The glumes are indicated in green, the lemmas in blue, the floret meristem (FM) in pink, and the SM in yellow. In this spike the IM has already transitioned to a terminal spikelet showing two glume primordia, one lemma primordium with an incipient FM, and the terminal SM. Note the rotated orientation of the terminal spikelet relative to the lateral spikelets, with the glume and lemma primordia originating in the same orientation as previous SMs. **E** Putative section plane at W3.5 explaining the absence of SM cells adjacent to the large FM in the central spikelets of Fig. 1. The large FM may displace the SMs outside the section plane.

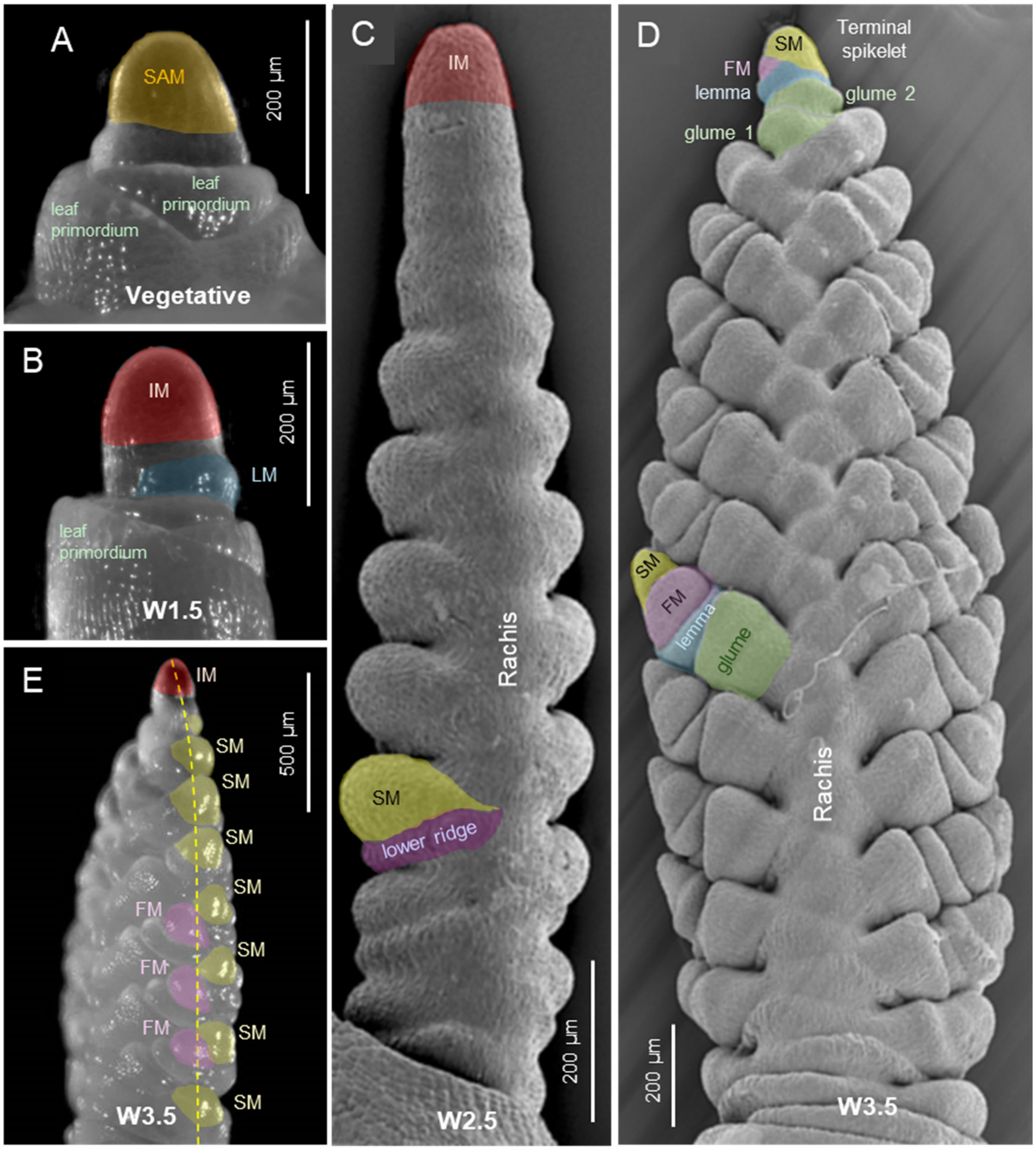

Additional file 1: Fig. S2. Additional sections showing cell segmentation and clustering. Cell clusters based on the expression of 99 genes studied by smFISH at three different stages of wheat spike development. **A** W1.5, initial transition from vegetative to reproductive stage (section D1-2, 4123 cells,). **B** W2.5, late double ridge (section B2-4, 7,491 cells). **C** W3.5, floret primordia stage (section A2-2, 11,742 cells). Same colors represent same cell clusters in all sections in Fig. 1 and S2. The inset in panel **B** is a vegetative bud at the base of the section (truncated to fit the three figures in the page). IM= inflorescence meristem, SM= spikelet meristem and FM= floret meristem.

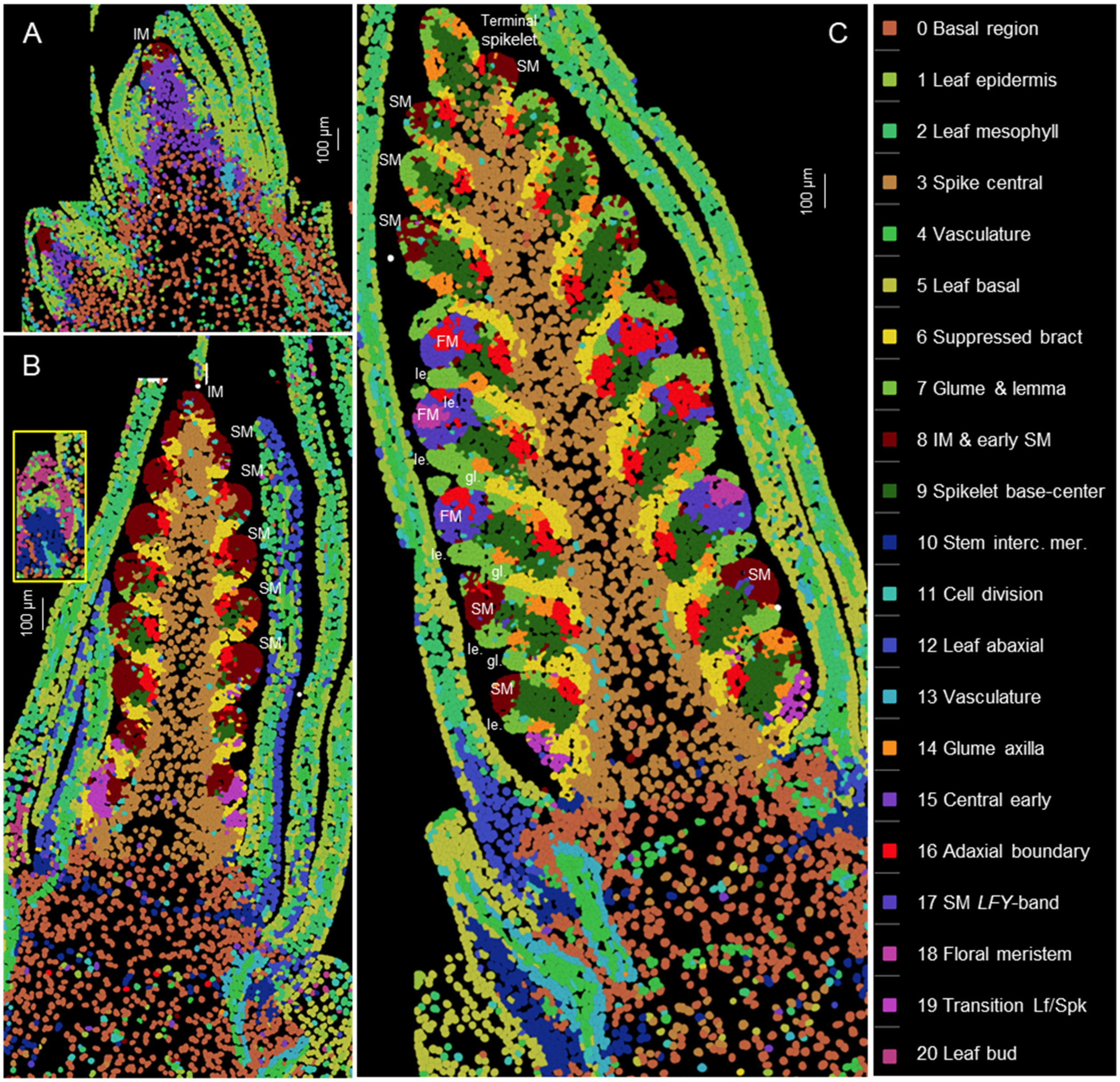

Additional file 1: Fig. S3. Calcofluor-white images of cell segmentation sections presented in Fig. 1. **A** W1.5, initial transition from vegetative to reproductive stage. **B** W2.5, late double ridge. **C** W3.5, floret primordia stage.

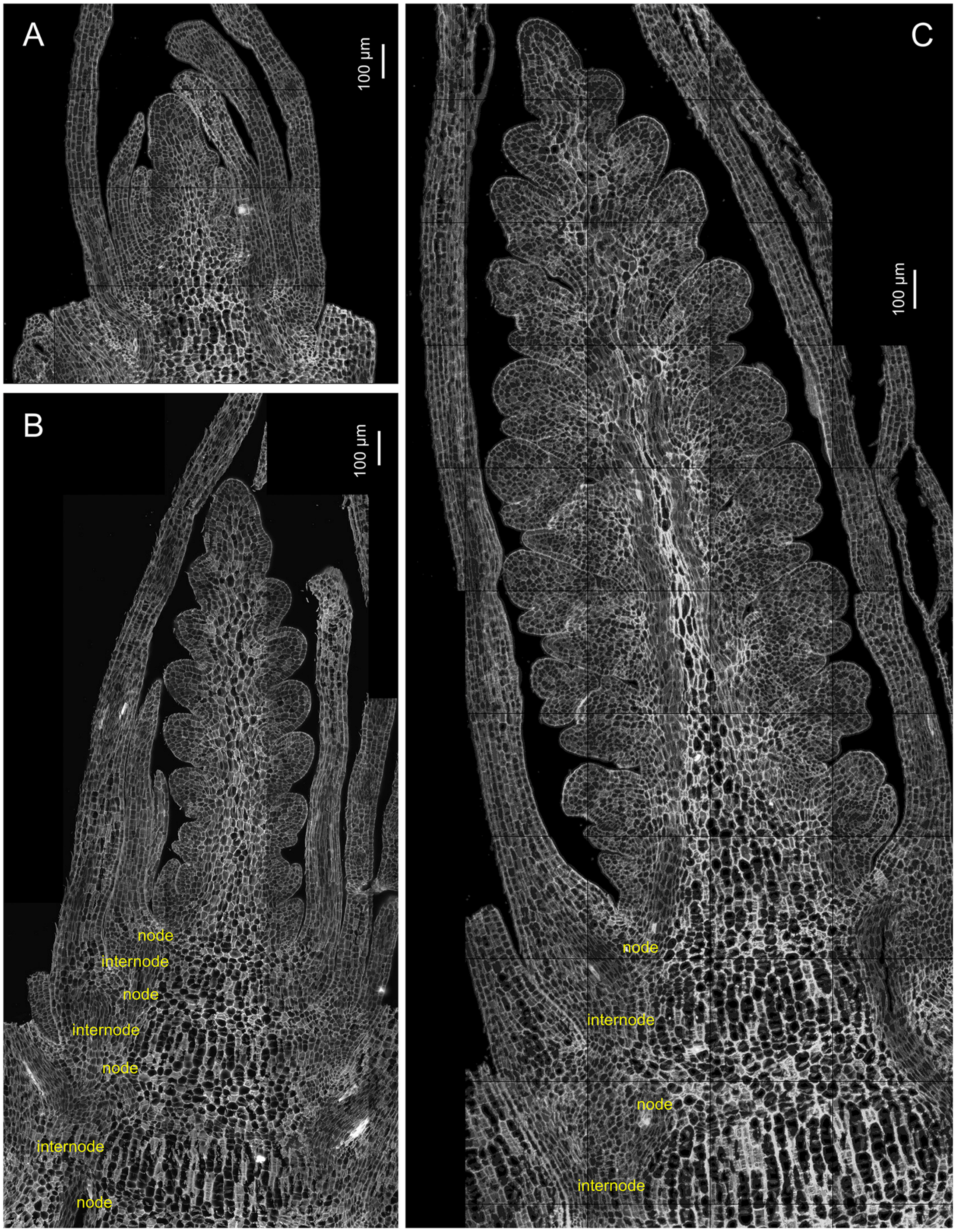

Additional file 1: Fig. S4. Calcofluor-white images of cell segmentation sections in Additional file 1: Fig. S2. A W1.5, initial transition from vegetative to reproductive stage. **B** W2.5, late double ridge. **C** W3.5, floret primordia stage. The inset in panel B is a vegetative bud at the base (left) of the section, which was truncated to fit the three figures in the page.

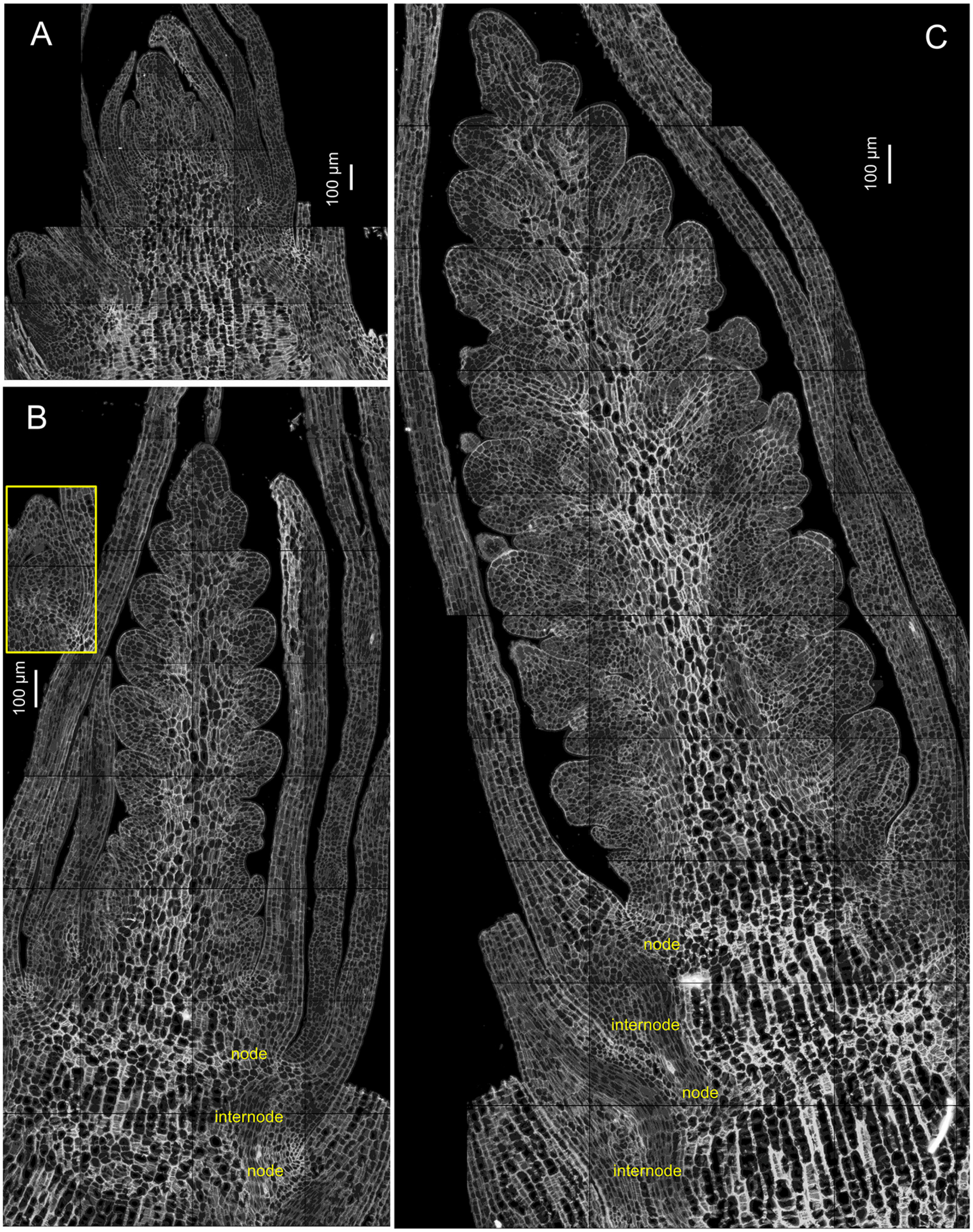

Additional file 1: Fig. S5. Individual cell clusters identified at three spike development stages (W3.5, W2.5 and W1.5). Cell clusters are numbered from 0 to 20 based on the expression of 99 genes studied by smFISH. Cells from all sections were clustered together. Cluster numbers are followed by the preferentially expressed genes in white and the preferentially not expressed genes in red.

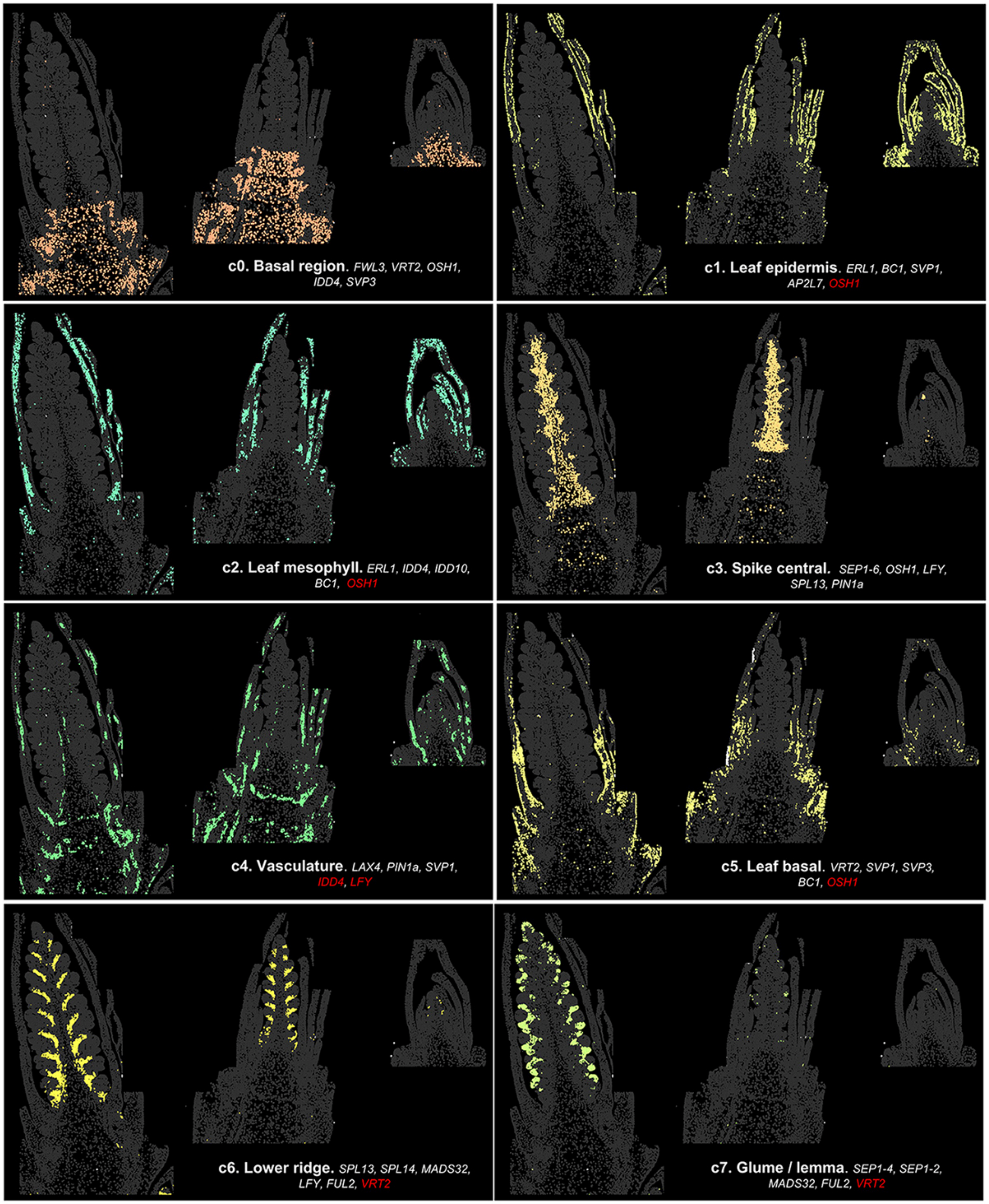

Additional file 1: Fig. S5. Continuation

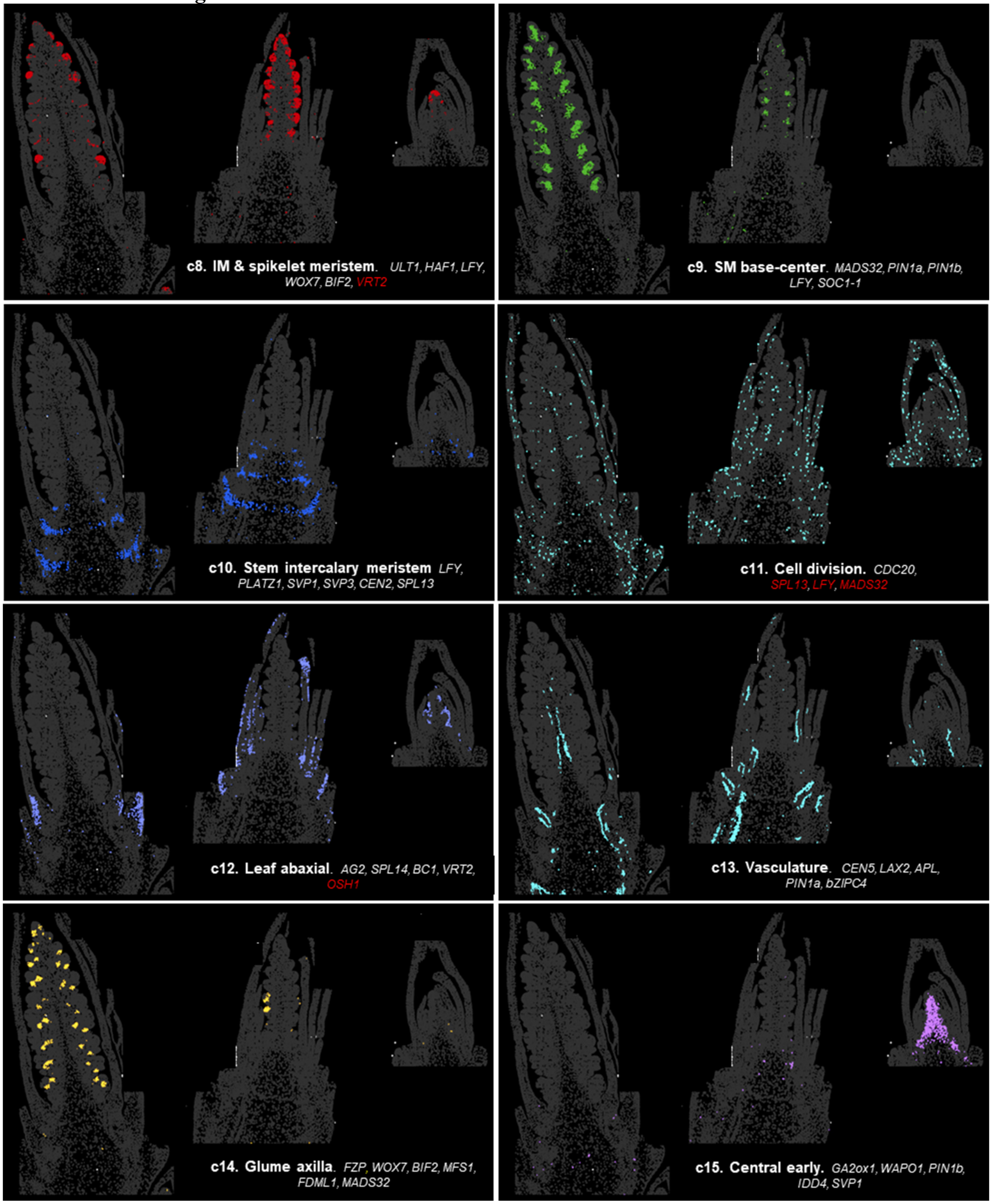

Additional file 1: Fig. S5. Continuation

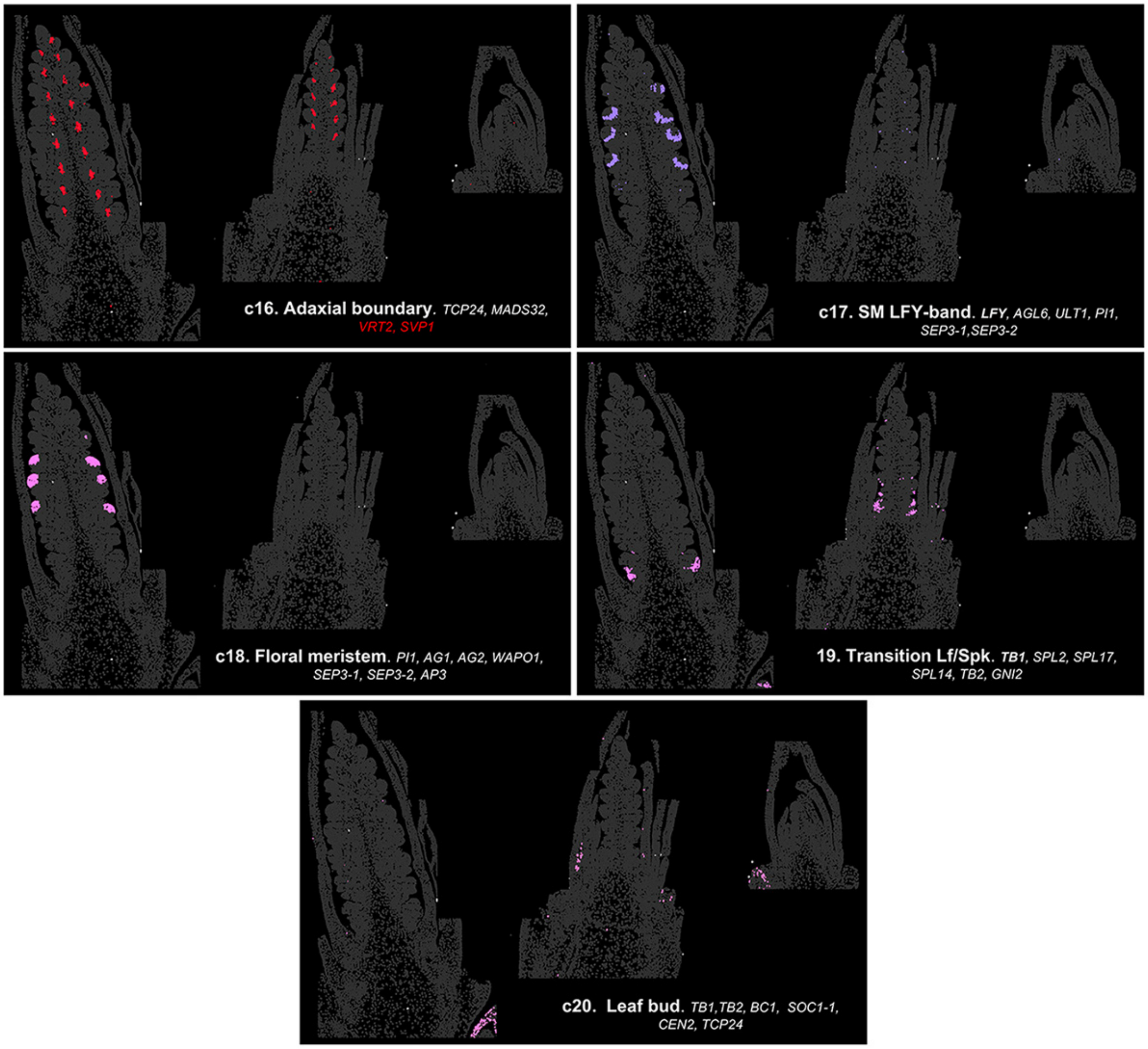

Additional file 1: Fig. S6. Validation of smFISH profiles. Comparison of Molecular Cartography expression profiles and *in situ* hybridization profiles previously obtained in our laboratory.

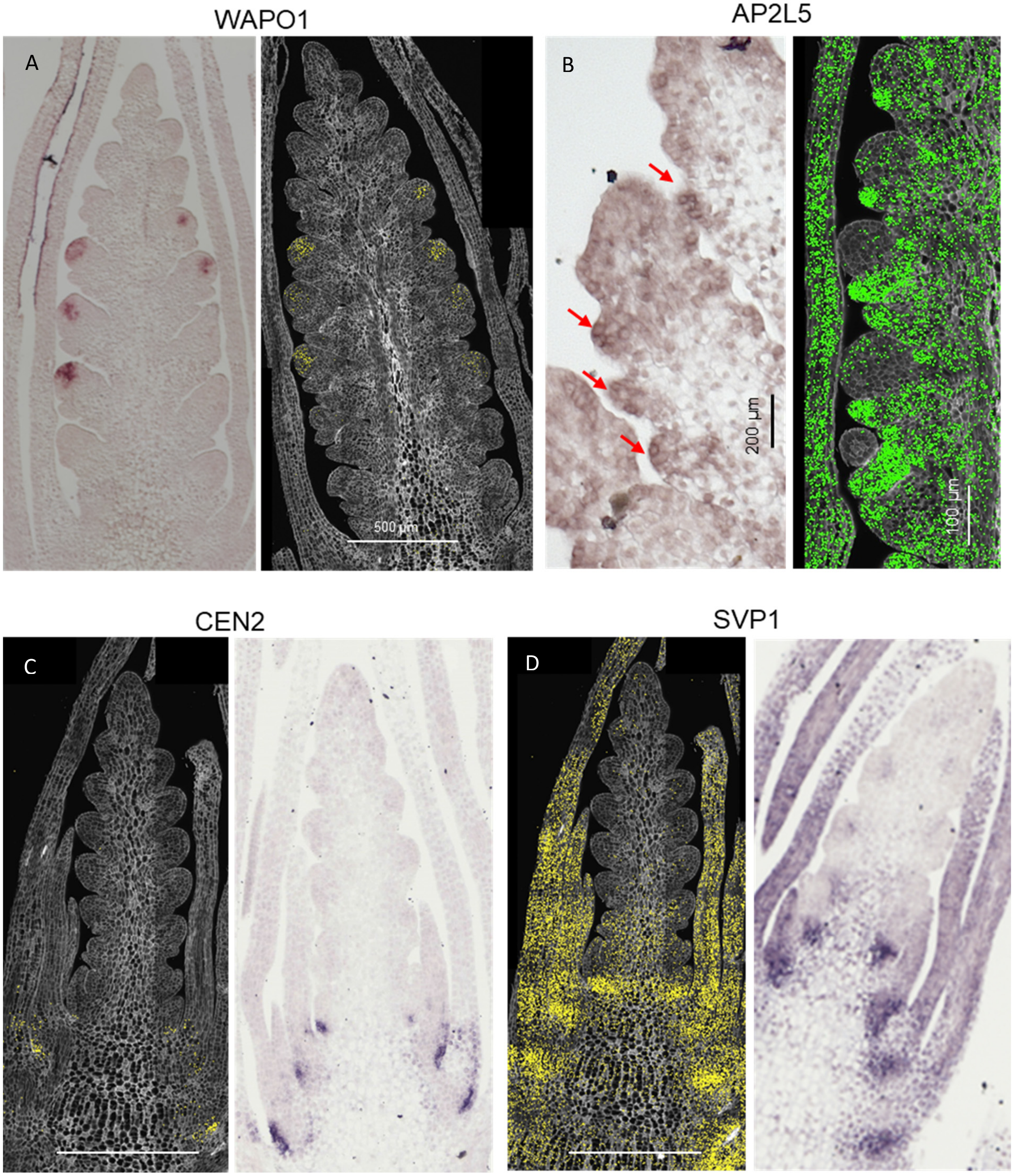

Additional file 1: Fig. S7. Validation of smFISH profiles. Comparison of Molecular Cartography expression profiles and MERFISH results in hexaploid wheat spikes at similar stages.

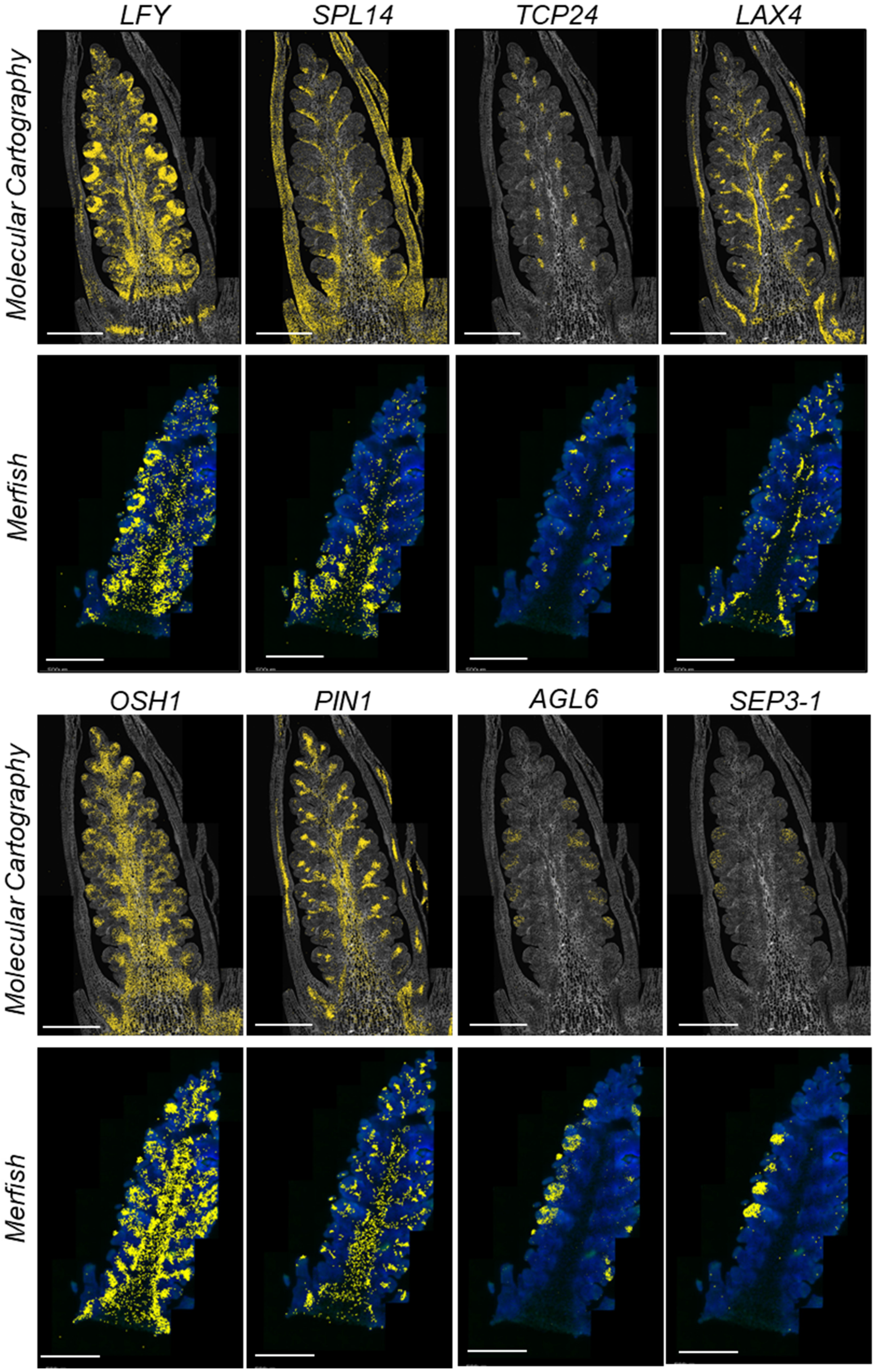

Additional file 1: Fig. S8. Basal region below the spike. Cell walls are stained with calcofluor-white. **A-B** W3.5. **C-D** W2.5. **A** *LFY, PLATZ1, ULT1*, and *SVP1*. **B** *LFY* and *NAL1*. **C** *LFY, PLATZ1, ULT1* and vascular markers *LAX4* and *APL.* **D** *LFY, NAL1*, and *SVP1*.

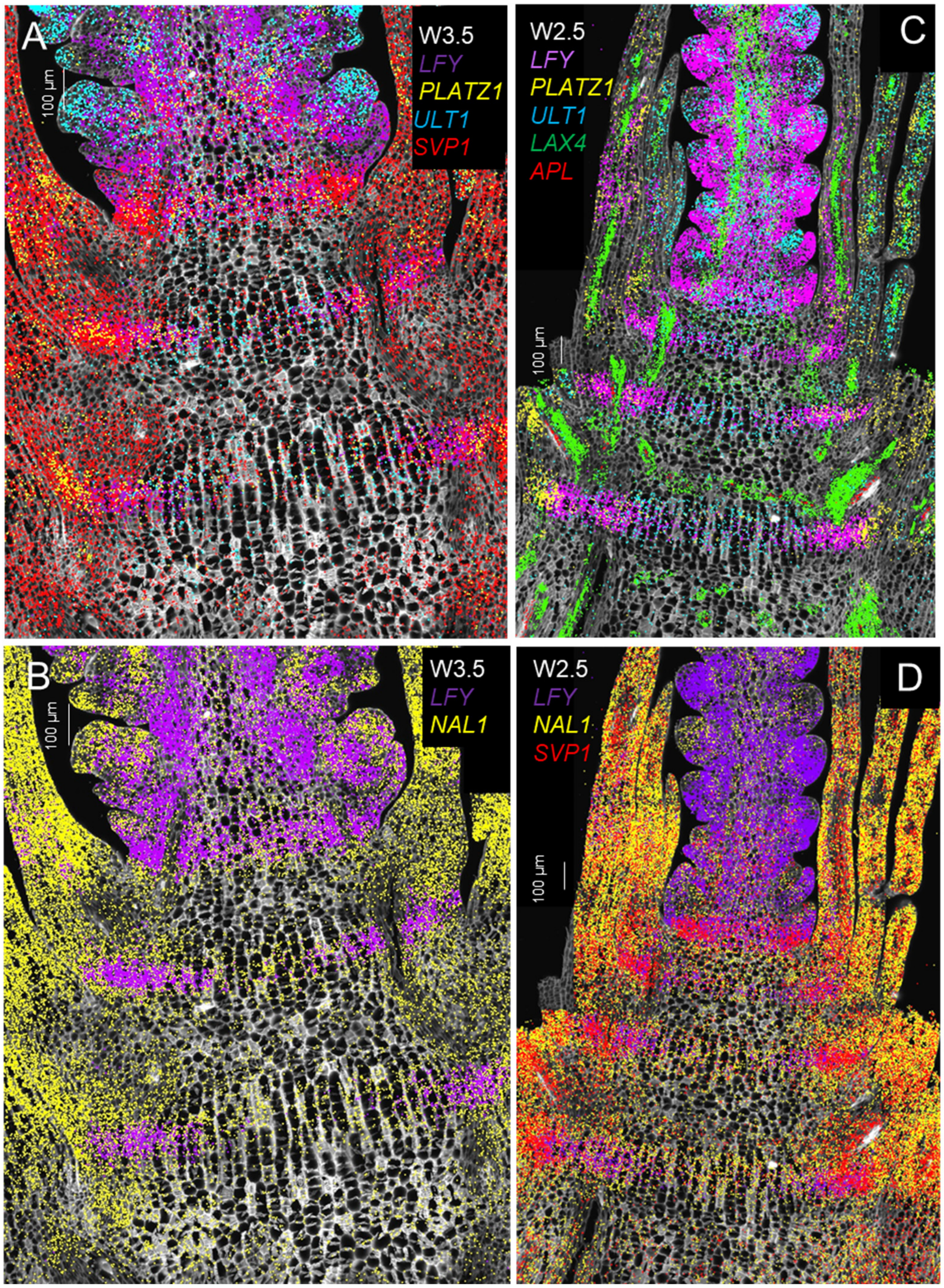

Additional file 1: Fig. S9. Functional validation of *LFY* role in the intercalary meristem. Effect of combined loss-of-function mutations in both *LFY* homeologs on **A** node diameter in mm, **B** internode diameter in mm, and **C** hollow or solid stem internodes. Internodes and nodes are numbered starting from the peduncle downward. Bars are averages of 5 wildtype-plants and 12 *lfy* mutant plants. Error bars are S.E.M. *= P< 0.05, **= P< 0.01, and ***= P< 0.001 using two-tailed t-Tests. Sections were stained with Toluidine Blue. Raw data and statistical analyses are available in Additional file 2: Table S4.

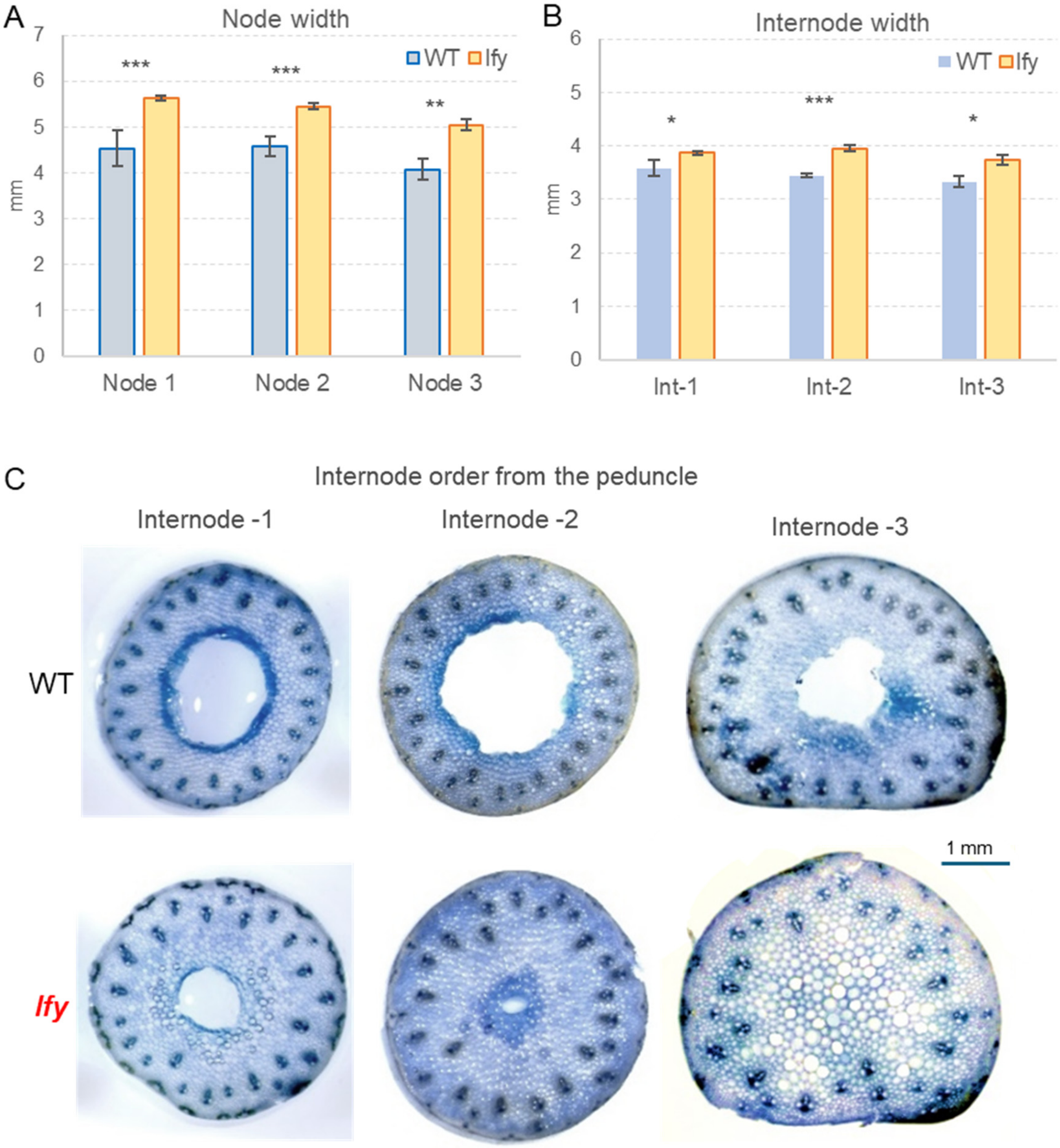

Additional file 1: Fig. S10. Expression of genes involved in the biosynthesis (*GA20ox1* and *GA20ox2*) and degradation (*GA2ox1*) of gibberellin in the basal region below spike. Cell from cluster c10 (stem intercalary meristem) are marked in blue. **A** W1.5. **B** W2.5. **C** W3.5. *GA20ox1* (yellow), *GA20ox2* (green), and *GA2ox1* (red).

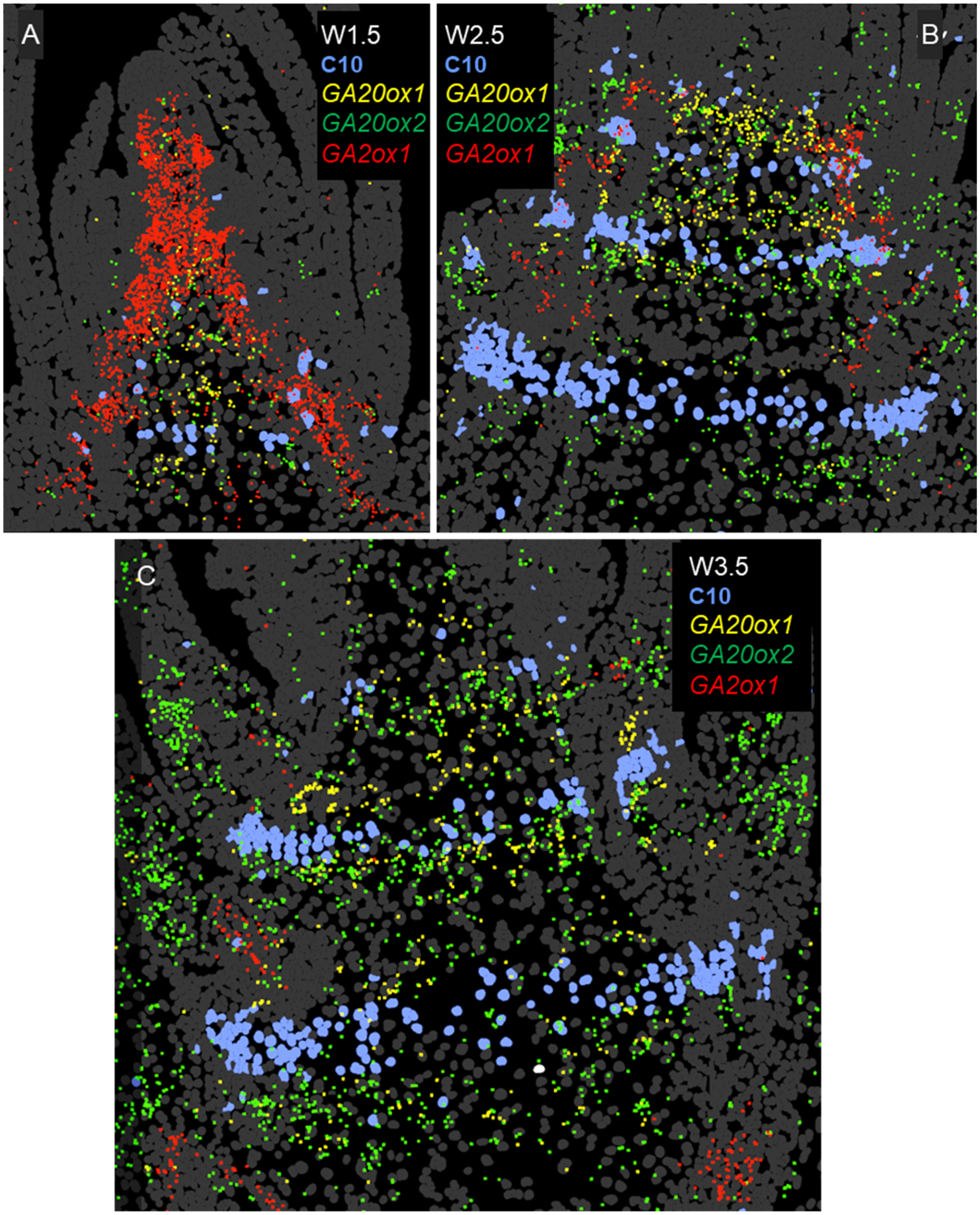

Additional file 1: Fig. S11. Differentially expressed genes in the transition zone between leaves and spike (cluster c19). A-B Cell included in cluster c9. Differentially expressed genes in c19 include **C-D** *SPL2, SPL14* and *SPL17* and **E-F** TB1 and TB2.

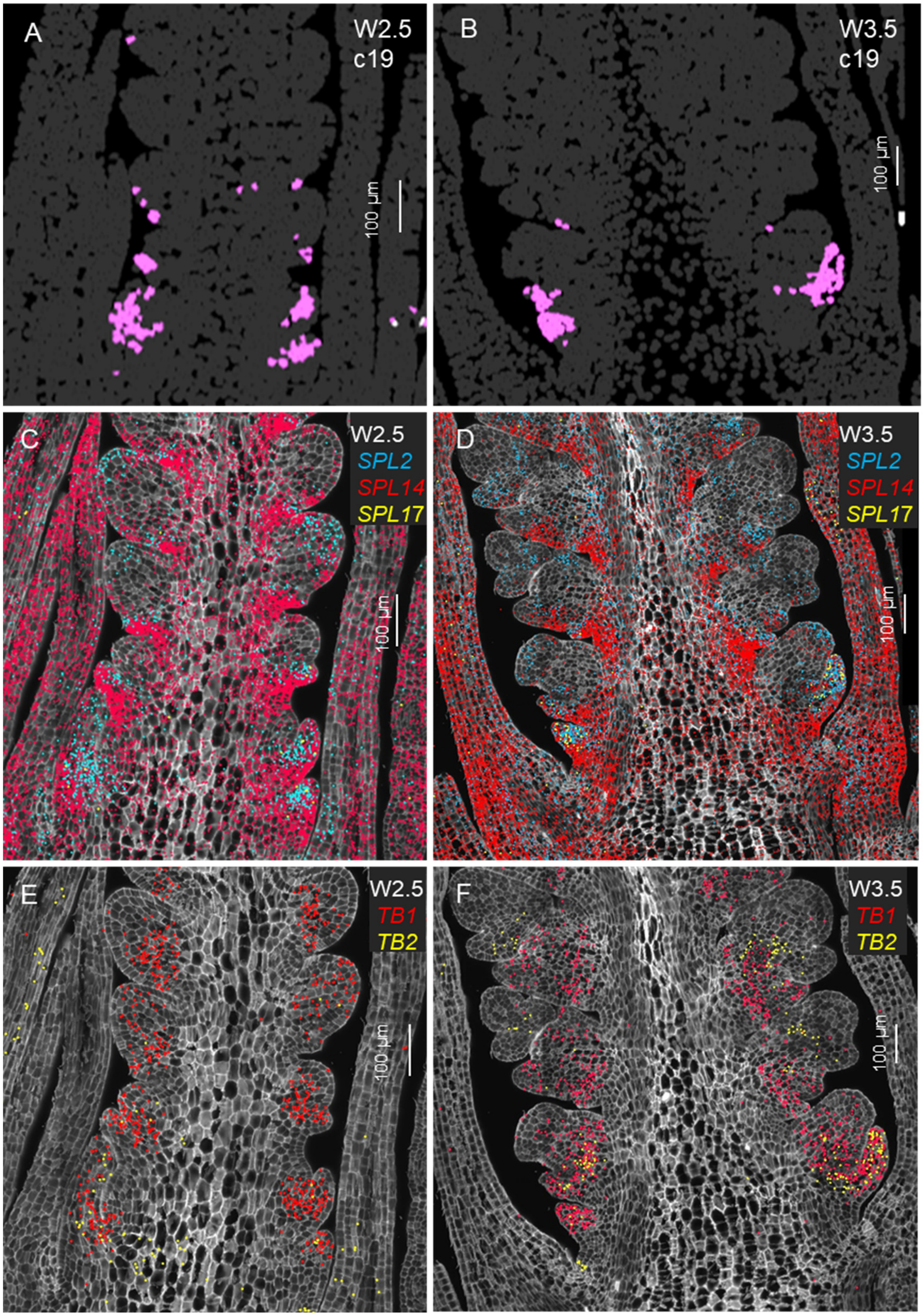

Additional file 1: Fig. S12. Spike central region with preferentially expressed genes. A, D W1.5 initial transition from vegetative to reproductive stages. **B, E** W2.5 late double ridge stage. **C, F** W3.5 floret primordia stage. Cells are stained with calcofluor-white in D-F. **A-C** *AP3* in yellow and *bZIPC4* in pink. **D-F** *IDD4* in yellow and *OSH1* in red. *AP3* genes are also expressed in stamen primordia in the central spikelets at W3.5.

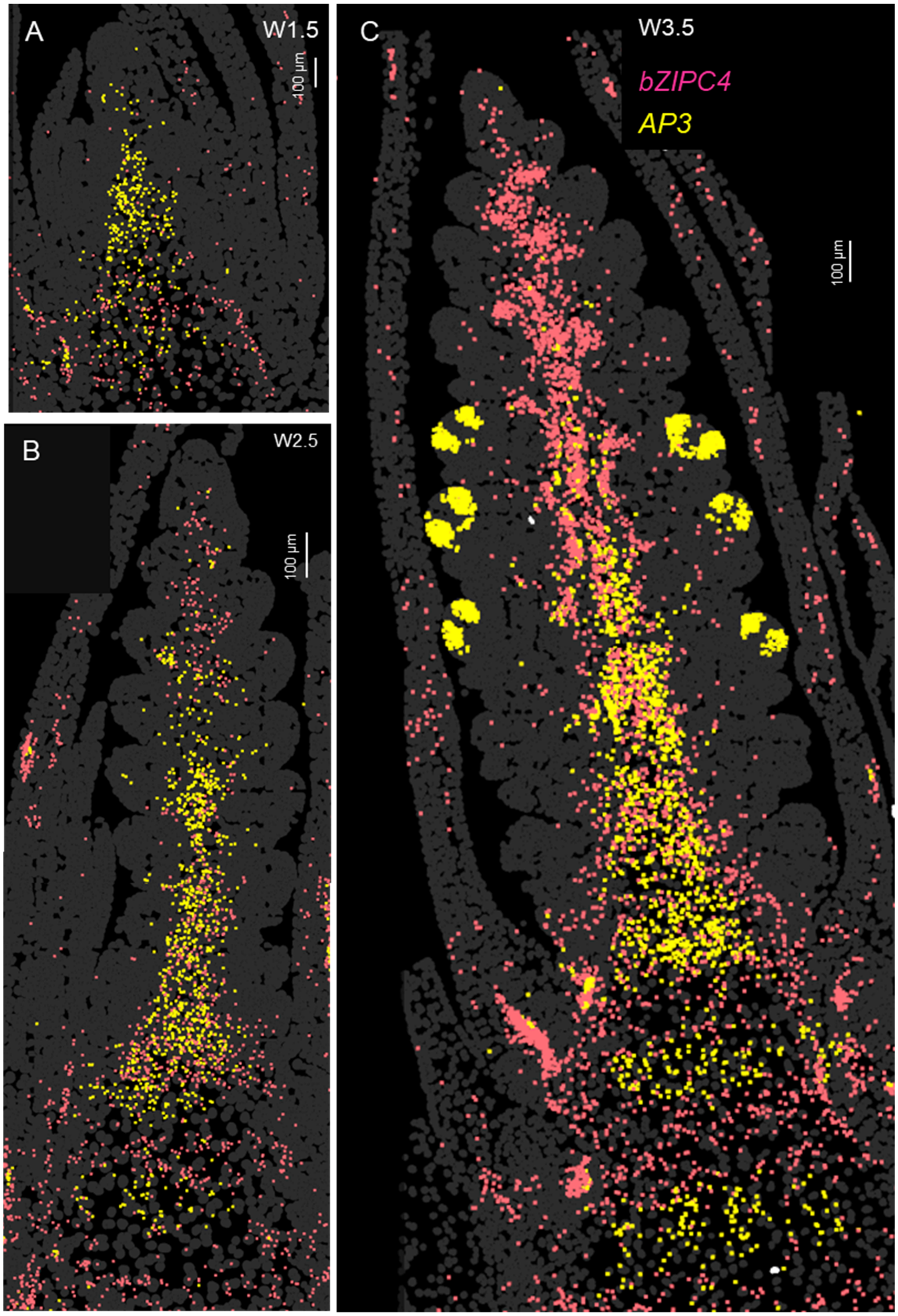

Additional file 1: Fig. S12. Spike central region differential genes. Continuation.

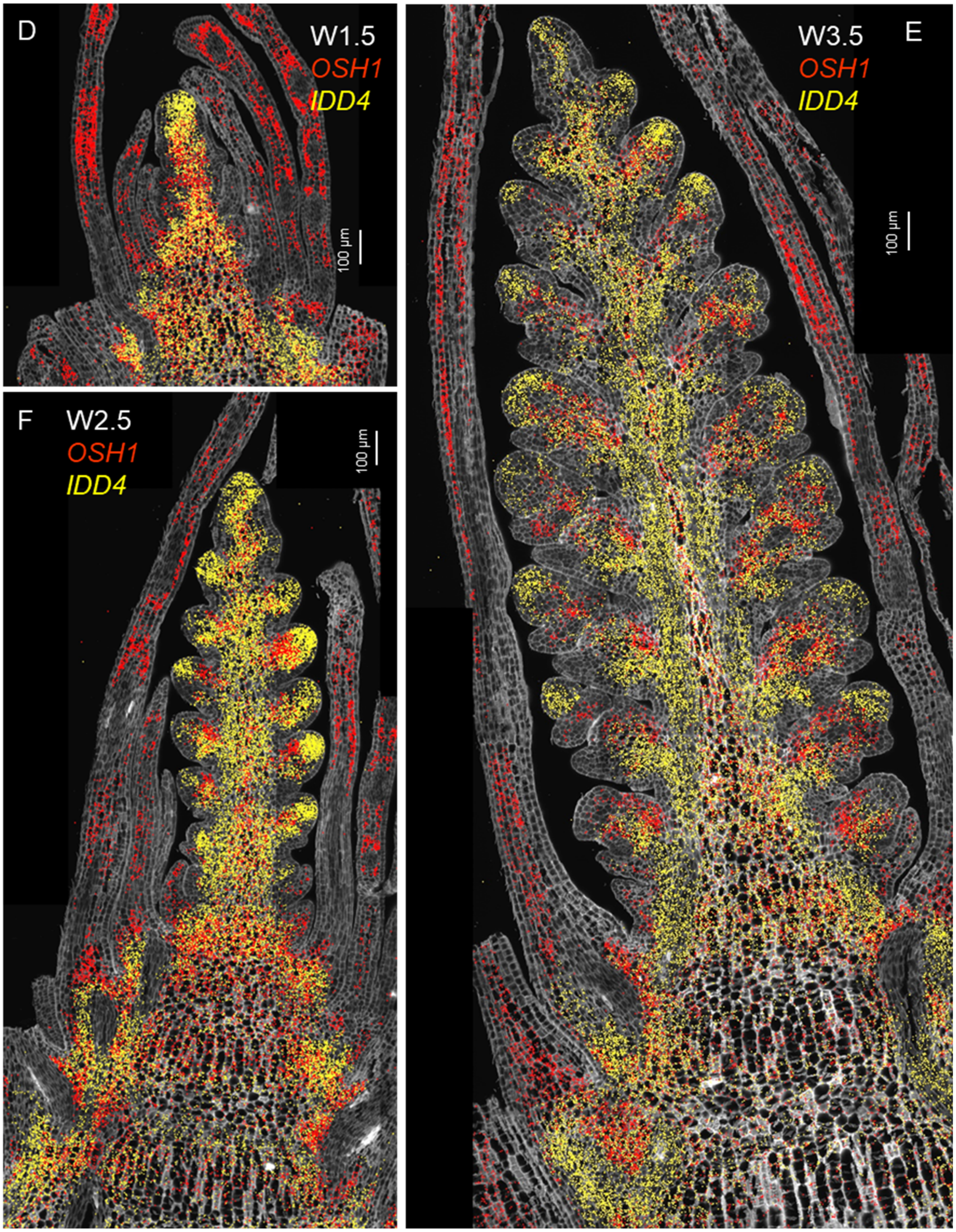

Additional file 1: Fig. S13. Genes preferentially in vasculature cell clusters sc4 and sc13 at W3.5. A Vasculature clusters c4 (green) and c13 (violet). Same cells with overlapping gene expression: **B** *APL* is a phloem marker located mainly outside c4 and c13, whereas *CEN5* overlaps with c13, **C** *LAX4*, **D** *PIN1a*.

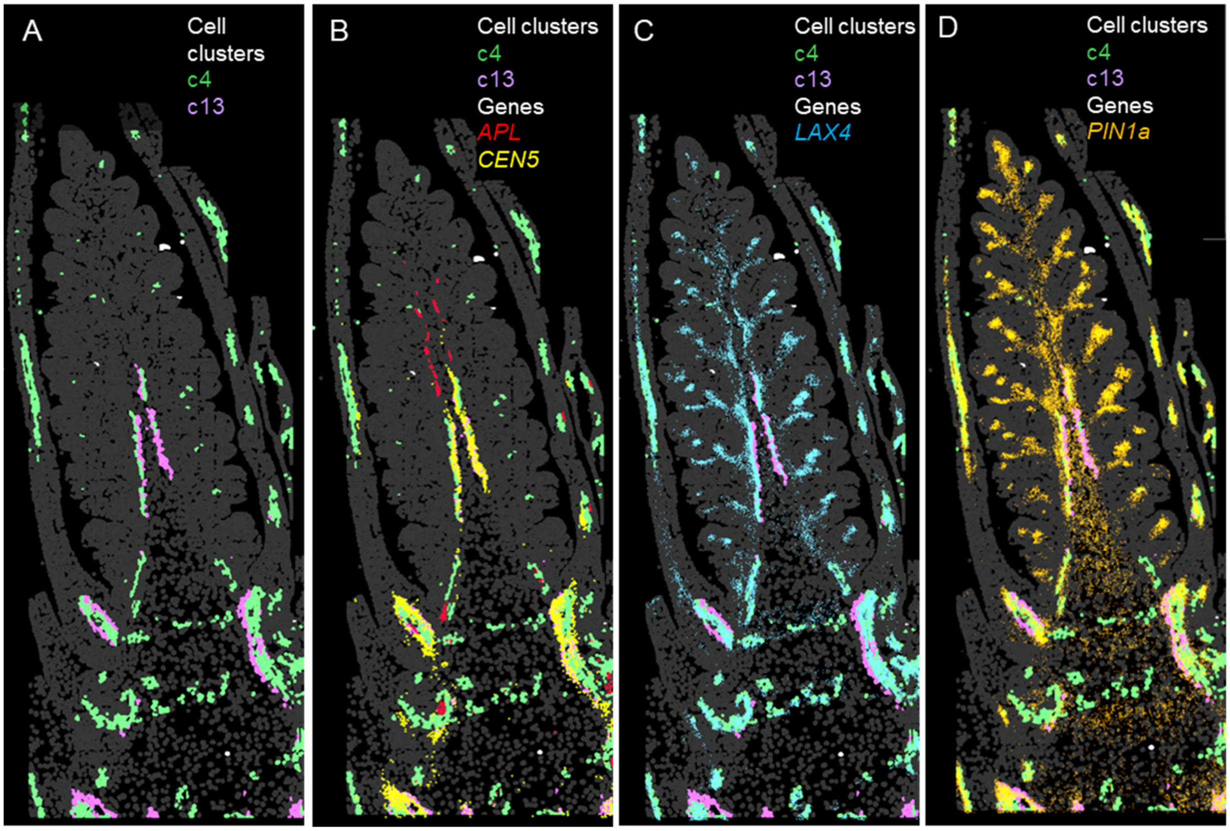

Additional file 1: Fig. S14. Genes preferentially expressed in the suppressed bract region (cluster c6). A, C W2.5 late double ridge stage. **B**, **D-F** W3.5 floret primordia stage. **A-B** *SPL14.* **C-D** *SPL13.* **E-F** Heatmaps: base of the spike at W3.5. **E** *FUL2.* **F** *VRN1.* **G** Combined *vrn1 ful2* mutant showing bract outgrowth (lost repression of lower ridge), indeterminate IM and vegetative lateral meristems).

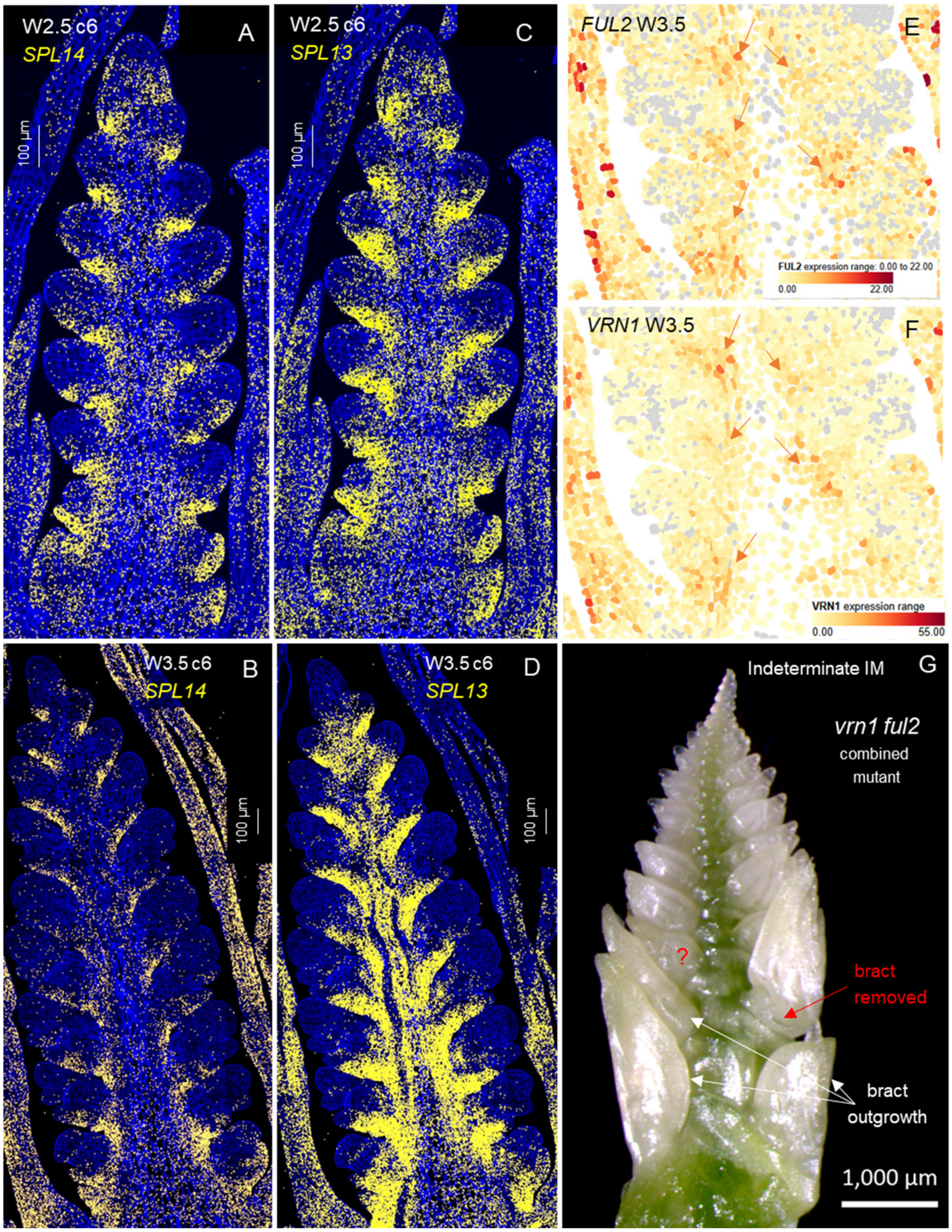

Additional file 1: Fig. S15. Expression profiles of diagnostic genes for cell clusters at the base of the developing spikelet. A W2.5 late double ridge stage. **B** W3.5 floret primordia stage. *SPL14* is preferentially expressed in the suppressed bract region (c6). *PIN1a* marks the developing spikelet vasculature (c9). *FZP* is expressed at the glume axilla (c14). *TCP24* is preferentially expressed at the adaxial boundary of the SM basal region (c16). *SEP1-4* is preferentially expressed in glumes and lemmas (stronger in glumes) and *SEP1-2* in lemmas at W3.5 (c7). Cell walls are stained with calcofluor-white.

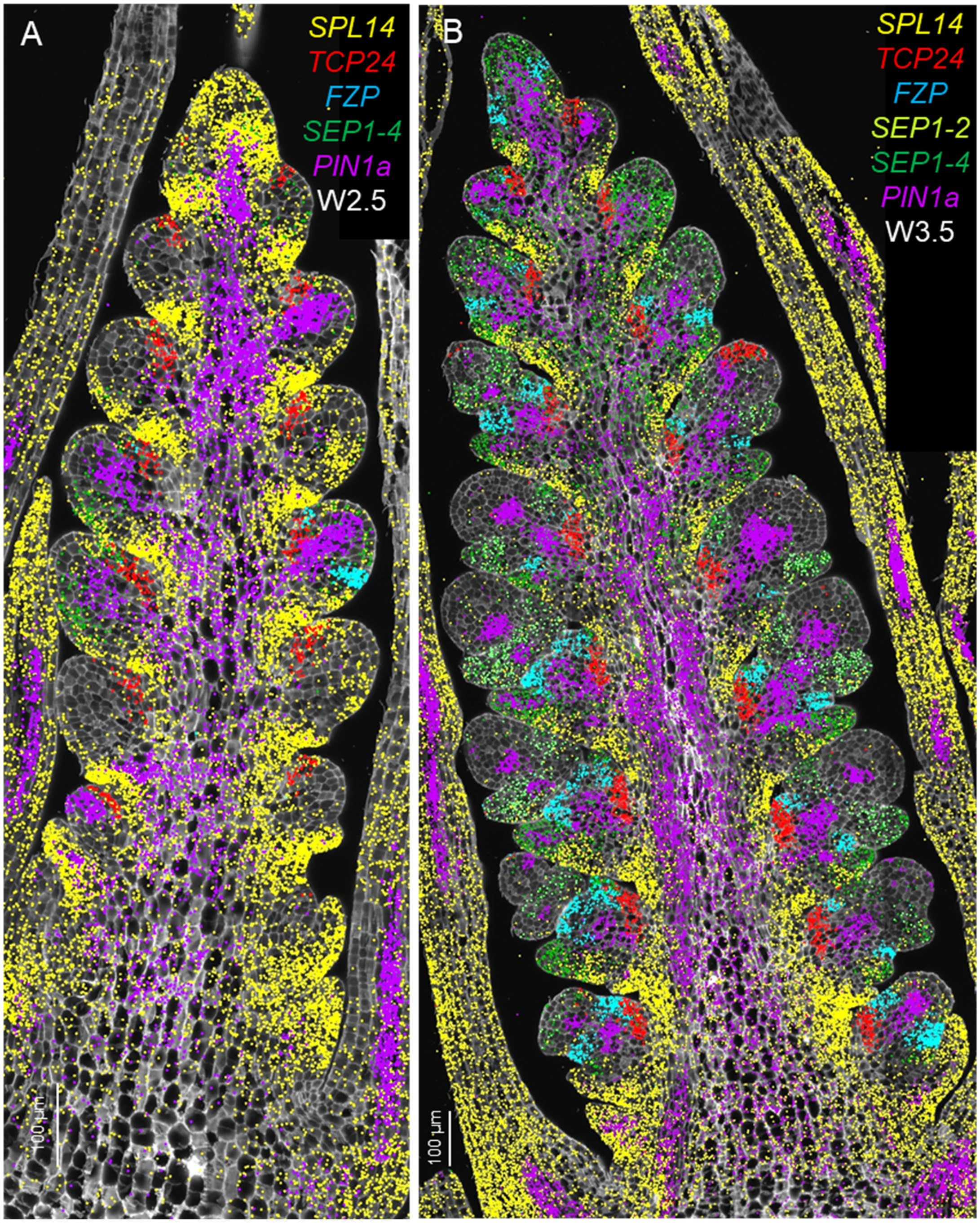

Additional file 1: Fig. S16. Expression profiles of genes preferentially expressed in cluster c14 at W3.5 (in addition to *FZP*). A-B Cells from cluster c14 (*FZP*) are marked in blue and those from c16 in violet (*TCP24*). **A** *WOX7*. **B** *BIF2*. These two genes overlap well with the cells of c14 but are also present in other cells outside c14. **C** Detail of W3.5 stained with calcofluor-white: *FZP* in blue, *BIF2* in yellow and *WOX7* in red.

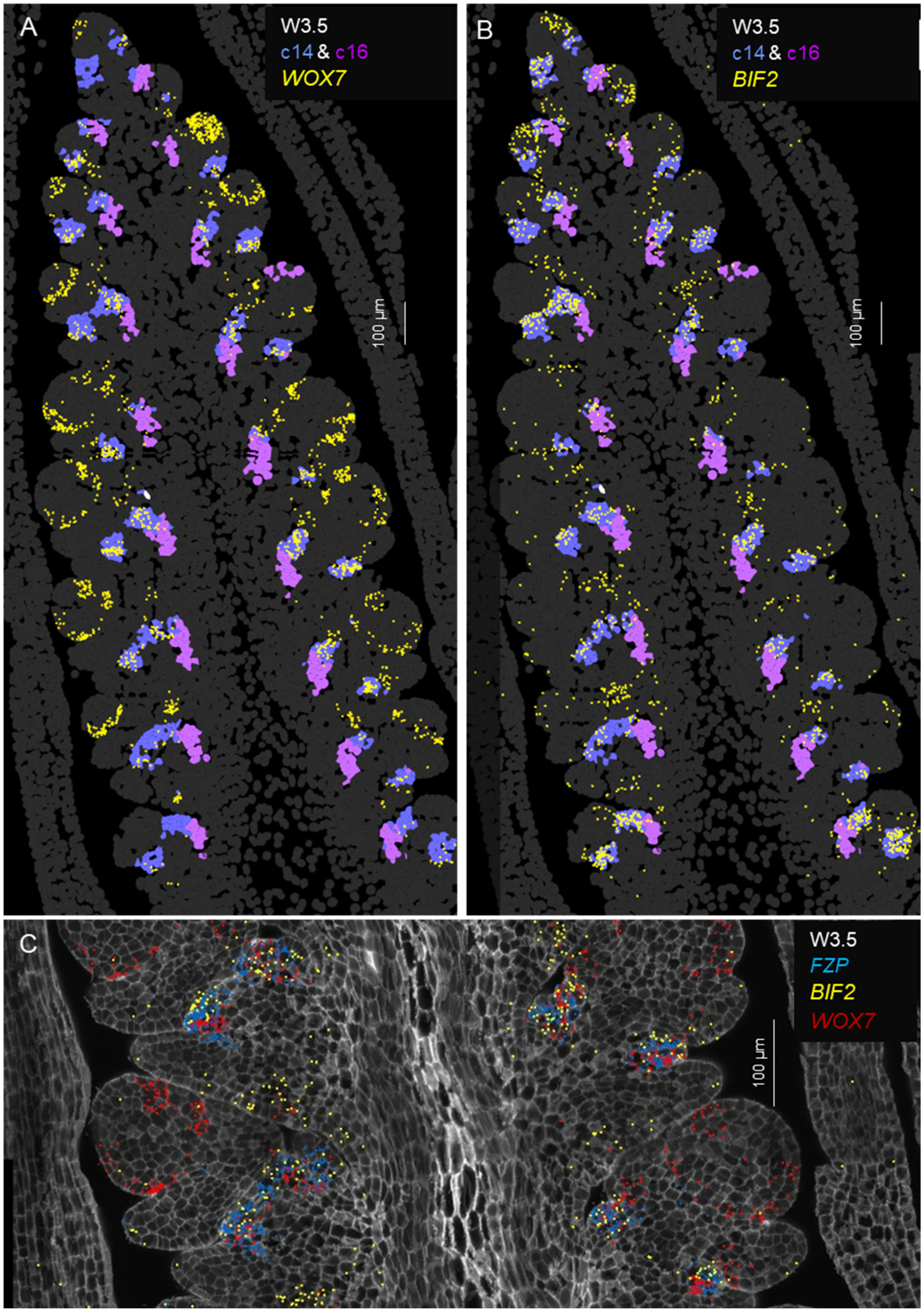

Additional file 1: Fig. S17. Functional validation of *FZP*. Effect of combined loss-of-function mutations in *fzp-A* and *fzp-B* (*fzp*) on spike development. **A** Schematic representation of *FZP* gene structure and location of the CRISPR induced deletions. **B-C** Spike of **B** wildtype and **C** *fzp*. **D-E** Detail of a spike node with a spikelet in **D** wildtype and **E** *fzp*. **F** Dissection of recurrent glumes at *fzp* node indicated in panel D by a red square. **G** Abnormal floral organs observed among the recurrent glumes. **H** Rachilla at the same *fzp* node with abnormal flowers. **I** Scheme of the developmental steps leading to the recurrent glumes: In each glume a new axis emerges from the axillary meristem and produces additional glumes that repeats the same process. At each cycle, fewer and smaller glumes are formed, until the SM is exhausted. The axes at different cycles are indicated by different colors (black → blue → green). Abnormal flowers are indicated by red circles. **J** Dissection of a wildtype Kronos spikelet (only the first floret is shown.). **K** Scheme of a wildtype Kronos spikelet.

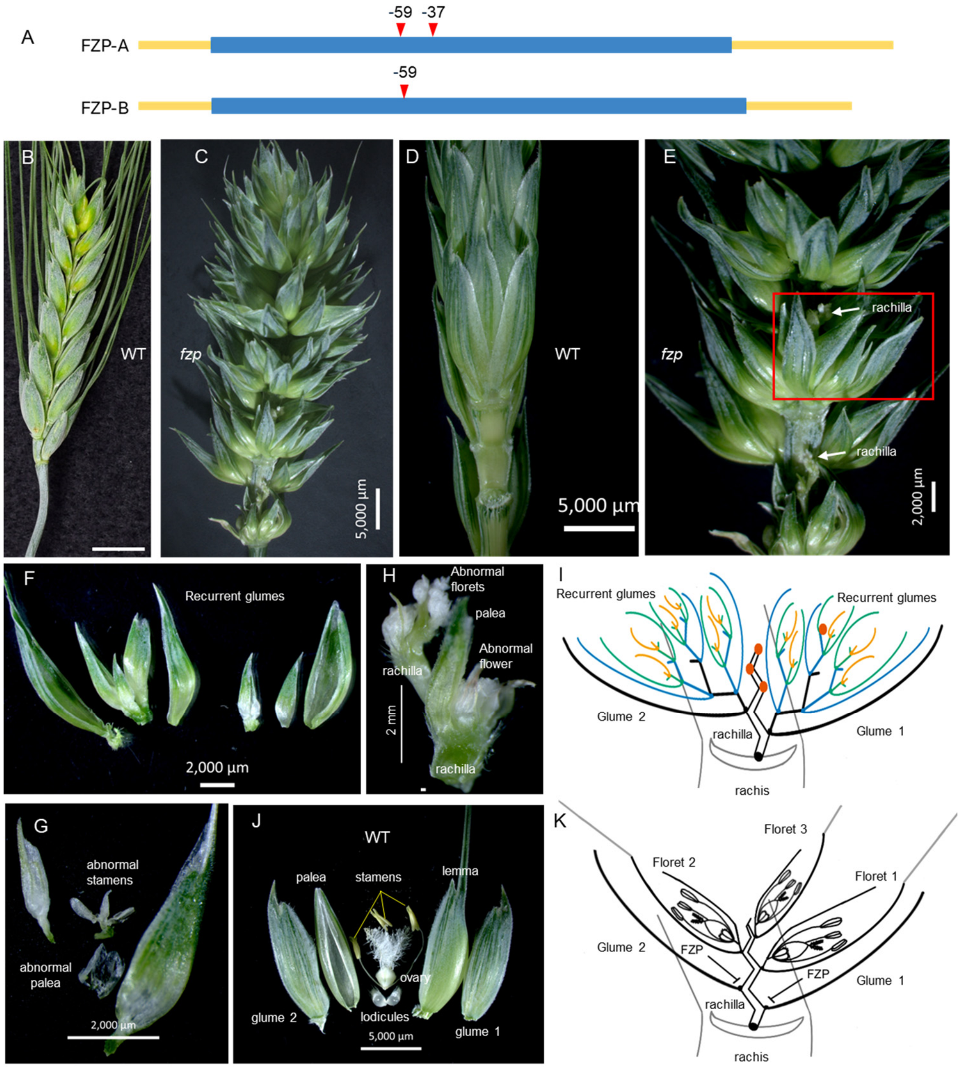

Additional file 1: Fig. S18. Expression profiles of genes preferentially expressed in the spikelet meristem (SM). A *HAF1* and *BIF2*. **B** *HAF1* and *AGL6* **C** *ULT1* and *WOX7*. **D** *ULT*, *WOX7* and *BIF2*. **A-C** W2.5 late double ridge stage. **B-D** W3.5 floret primordia stage. c8 = early SM development, c18 = floret meristem FM. **E-G** Floral homeotic genes expressed the FM (c18). **H** *WAPO1* is expressed at the FM in c18*,* whereas *LFY* is highly expressed in a band between the lemma primordia and the FM (c17).

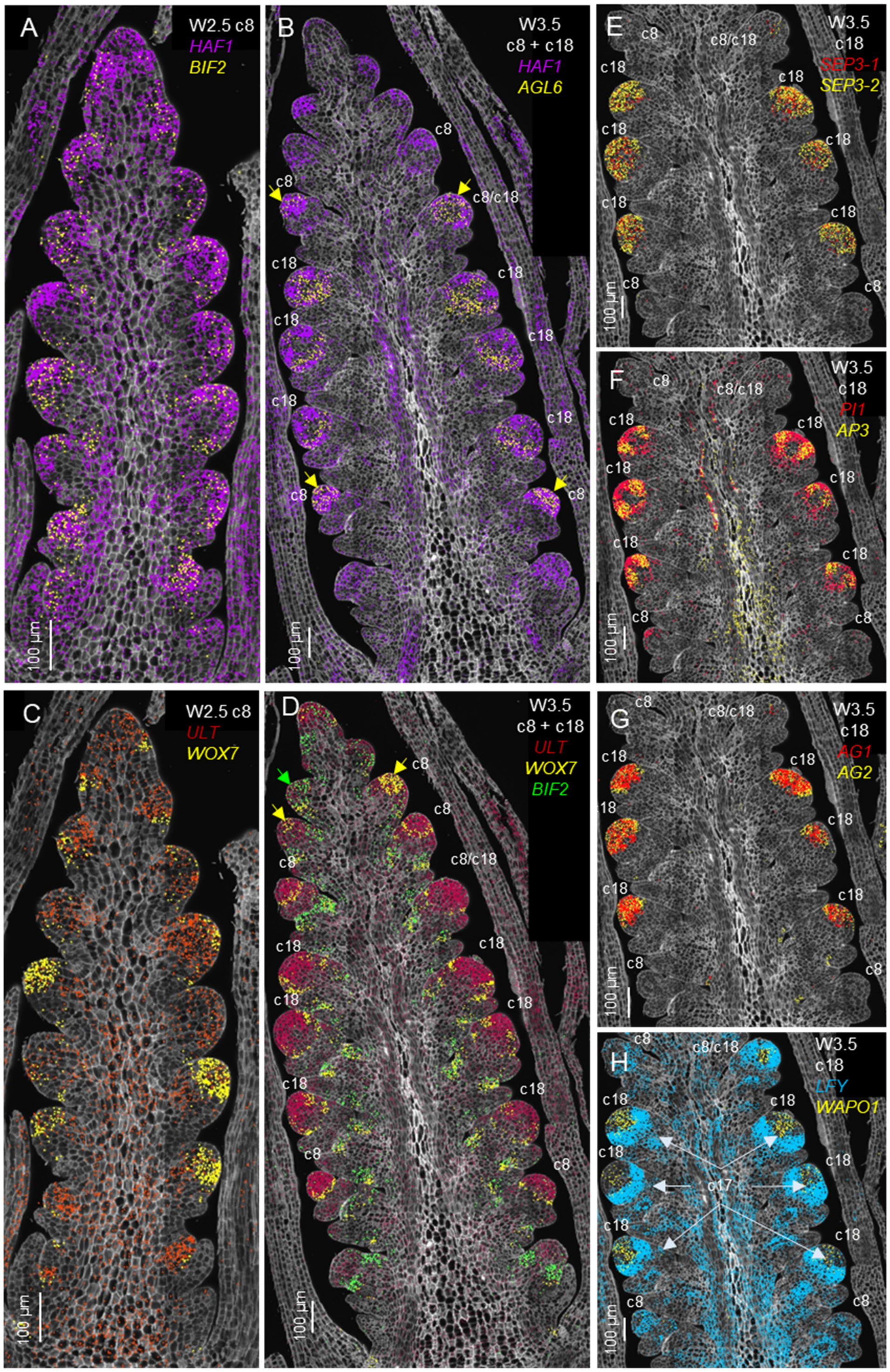

Additional file 1: Fig. S19. Selected regions in the inflorescence meristem. Example for the changes in hybridization density in the terminal and first two lateral meristems between W2.5 (IM+2LM) and W3.5 (SM + 2 glume primordia). **A** *SPL14* at W2.5. **B** *SPL14* at W3.5. **C** *FZP* expression at W3.5 is included as a control to show that the IM has already initiated the transition to a terminal spikelet.

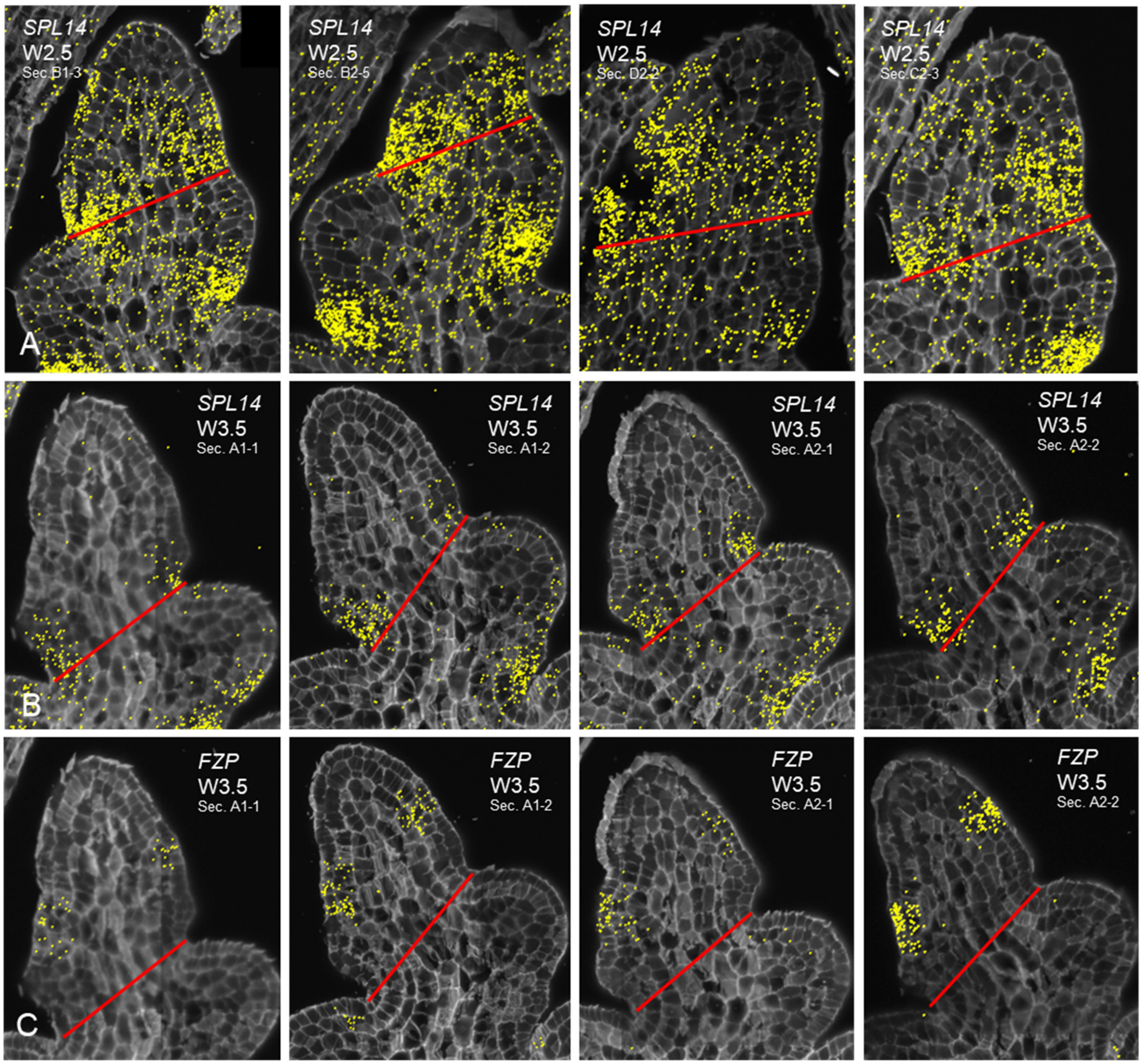

Additional file 1: Fig. S20. Genes with significant differences in hybridization density in the terminal region between W2.5 and W3.5. The selected region included the terminal meristem and the two youngest lateral meristems. At W2.5 the TM= IM and LM= SM, whereas at W3.5 TM= SM and LM= glume meristems. Genes downregulated at W3.5: **A** *SPL14*, and **B** *HAF1*. Genes up-regulated in W3.5: **C** *BC1* **D** *FUL2*, **E** *SOC1-2*, **F** *SEP1-4*, **G** *bZIPC1*, **H** *bZIPC3*, **I** *FDL2*, **J** *FDL6* **K** *PIN1b*, and **L** *CRN*. Four different sections at W2.5 and two at W3.5 (each with two subsamples) were used in the analyses. * $P < 0.05$, ** $P < 0.01$, and *** $P < 0.001$ based on two-tailed *t*-Tests. *HAF1* was marginally not significant $P < 0.12$) but was included since there were not many downregulated genes. Raw data and statistical analyses are presented in Additional file 2: Table S5.

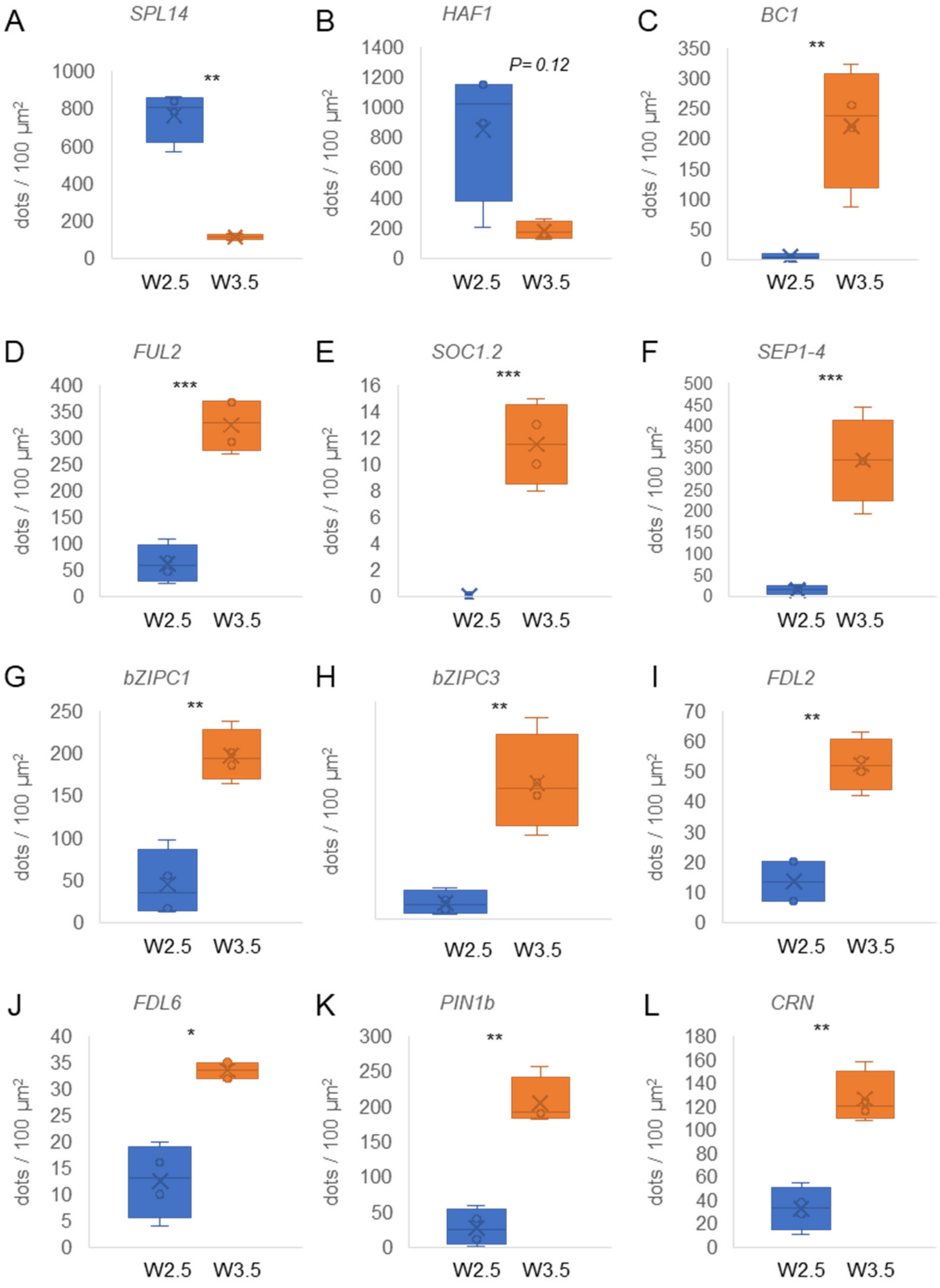

Additional file 1: Fig. S21. Functional validation of *SPL14*. **A** *SPL-A14* and *SPL-B14* gene structure and location of the CRISPR induced mutations **B** Conservation across different monocot species of the region including the frame shift insertion and premature stop in *spl-A14* and the C121 deletion in *spl-B14*. **C-H** Effect of the combined *spl-A14 spl-B14* mutations on **C & E** spikelet number per spike, **D &F** plant height, **G** heading time, and **H** leaf number. Plants are T_2 transgenic lines homozygous for mutations in both homeologs and without the CRISPR-CAS9 vector. Bars are the averages of 12 different T_1 homozygous edited plants and error bars are s.e.m. ***= $P<0.0001$. Raw data is available in Additional file 2: Table S6.

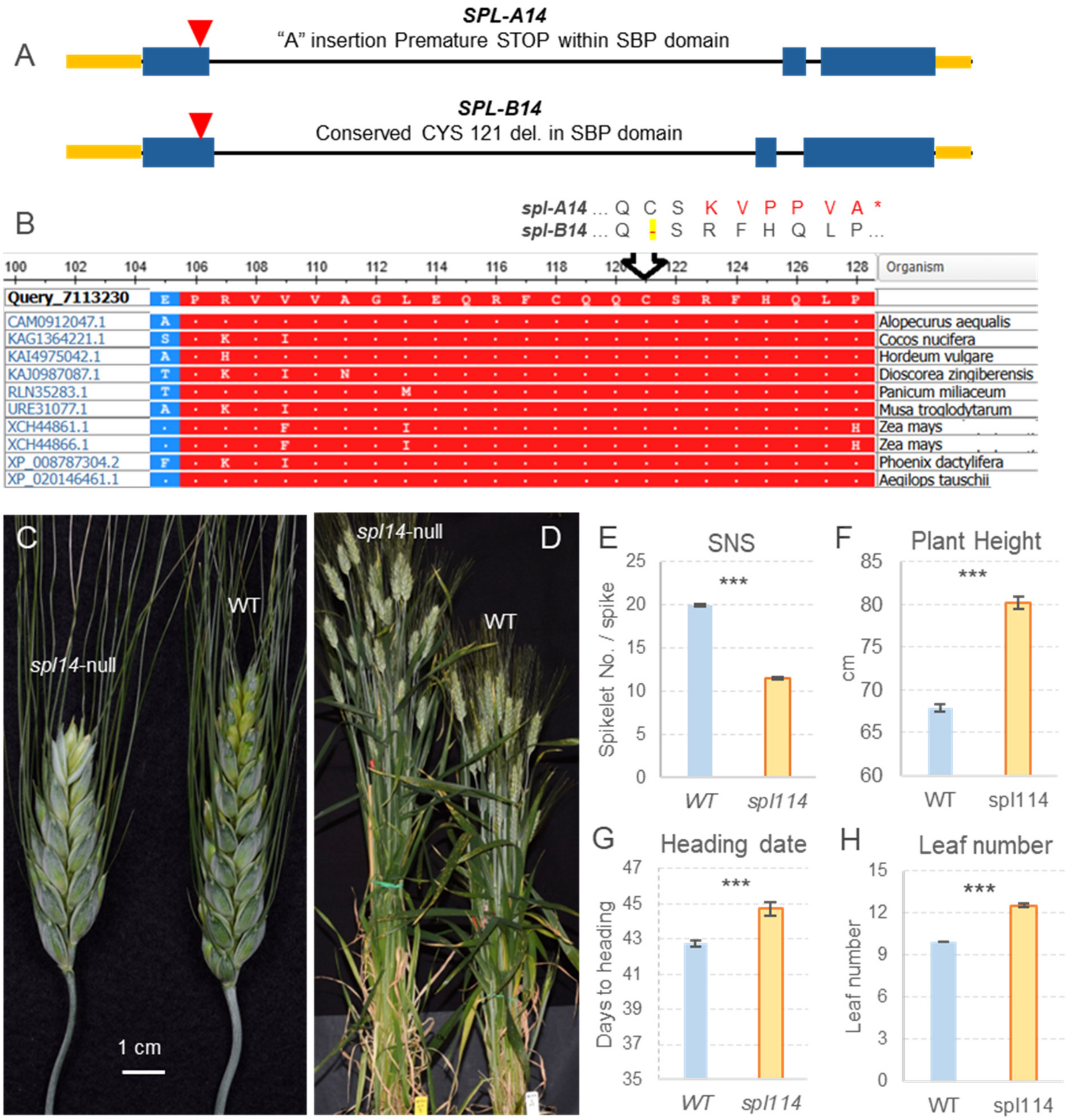

Additional file 1: Fig. S22. Trajectory analysis. The 21 cell clusters from the smFISH analysis were leveraged to constrain trajectory inferences generated with program Monacle 3. The cluster numbers and colors are the same as in Fig. 1. Cluster c8, which includes both inflorescence meristem (IM) and spikelet meristems (SM) was used as root. Starting from c8 one trajectory went towards the floral meristem (c17 and c18), and another one in the opposite direction towards the transition zone (c19) and the repressed bract (c6). A third trajectory trifurcated into the glume and lemma primordia (c7), the central cluster associated with the spikelet vasculature, and the boundary clusters including a trajectory that bifurcates into the glume axilla (c14) and the adaxial boundary (c16). The ground tissue at the center of the spike (c3) is connected by trajectories to both the meristem (c8) the suppressed bract (c6) and the leaves. Cluster c11, associated with dividing cells, was placed close to the spike meristems (c8) and the leaf buds (c20). Leaf clusters were not analyzed in this study.

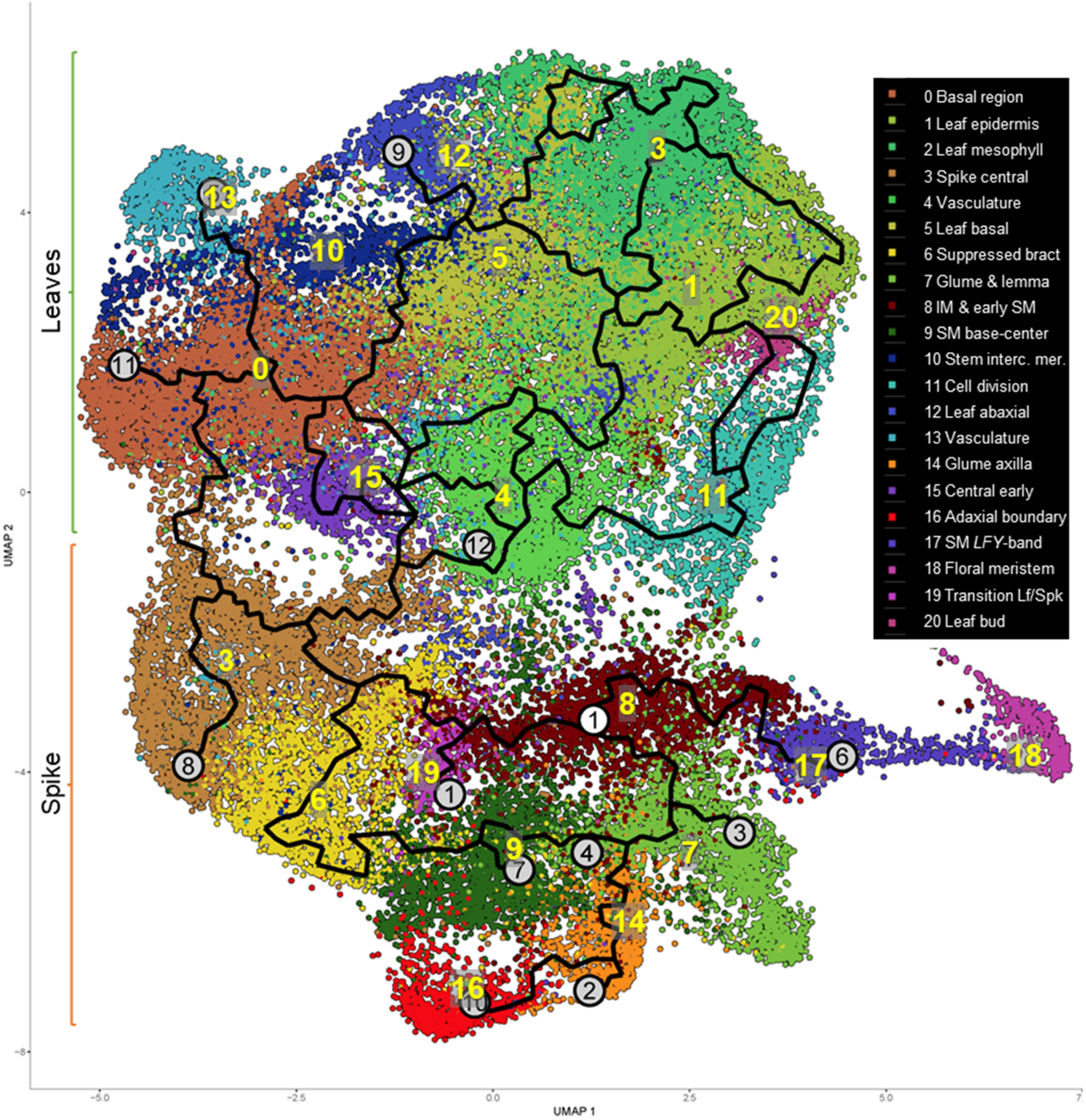

Additional file 1: Fig. S23. Comparison between initial and final scRNA-seq clustering. A Initial 25 clusters including 9 cell-cycle clusters showing good integration of the three replications and the two stages. **B** Identification of cells in the S (*HISA1*) and G2/M (*CYCB2*) phases in the UMAP. **C** Bubble-plots for *CYCB2* and *HIS2A1*. **D** Final 23 clusters used in this study showing good integration of the three replications and the two stages.

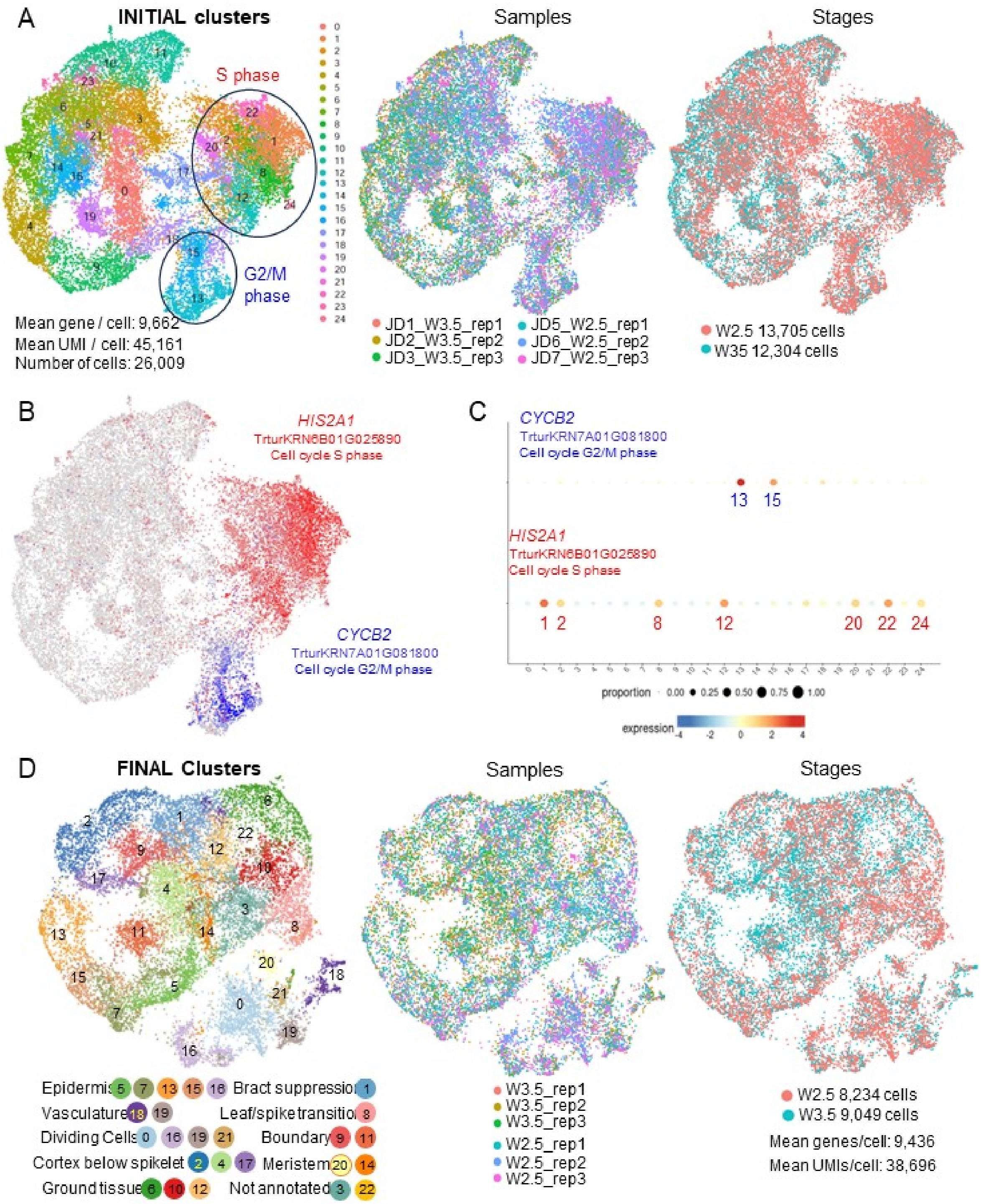

Additional file 1: Fig. S24. RNA-seq for three different sections of the spike. A Representative Kronos spike at W3.0 (glume development stage) and the three regions where the developing spikes were sectioned (dotted red lines). **B** Venn Diagram indicating the number of differentially expressed genes among the three different regions based on Tukey test (Additional file 2: Table S11). **C** Principal component analyses for the nine samples. Red color indicates the basal region, green the mid region and blue the tip. Different geometric figures indicate the three replications.

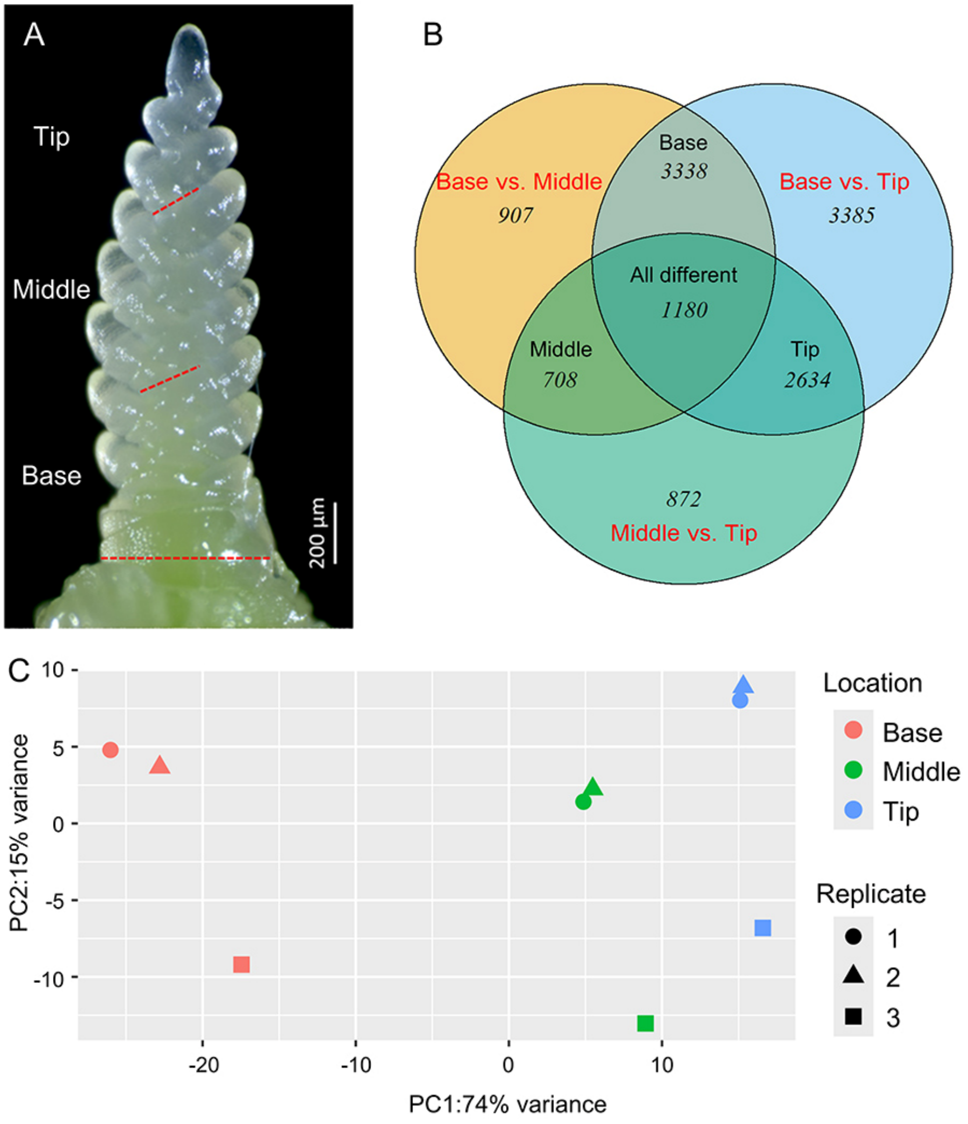

Additional file 1: Fig. S25. Cell cycle scRNA-seq clusters. A-F UMAPS and **G** bubble plots of known cell-cycle marker genes for clusters sc0, sc16, sc19 and sc21. Annotation of the marker genes and references are available in Additional file 2: Table S1. Additional genes preferentially expressed in cell-cycle clusters are available in Additional file 2: Table S9.

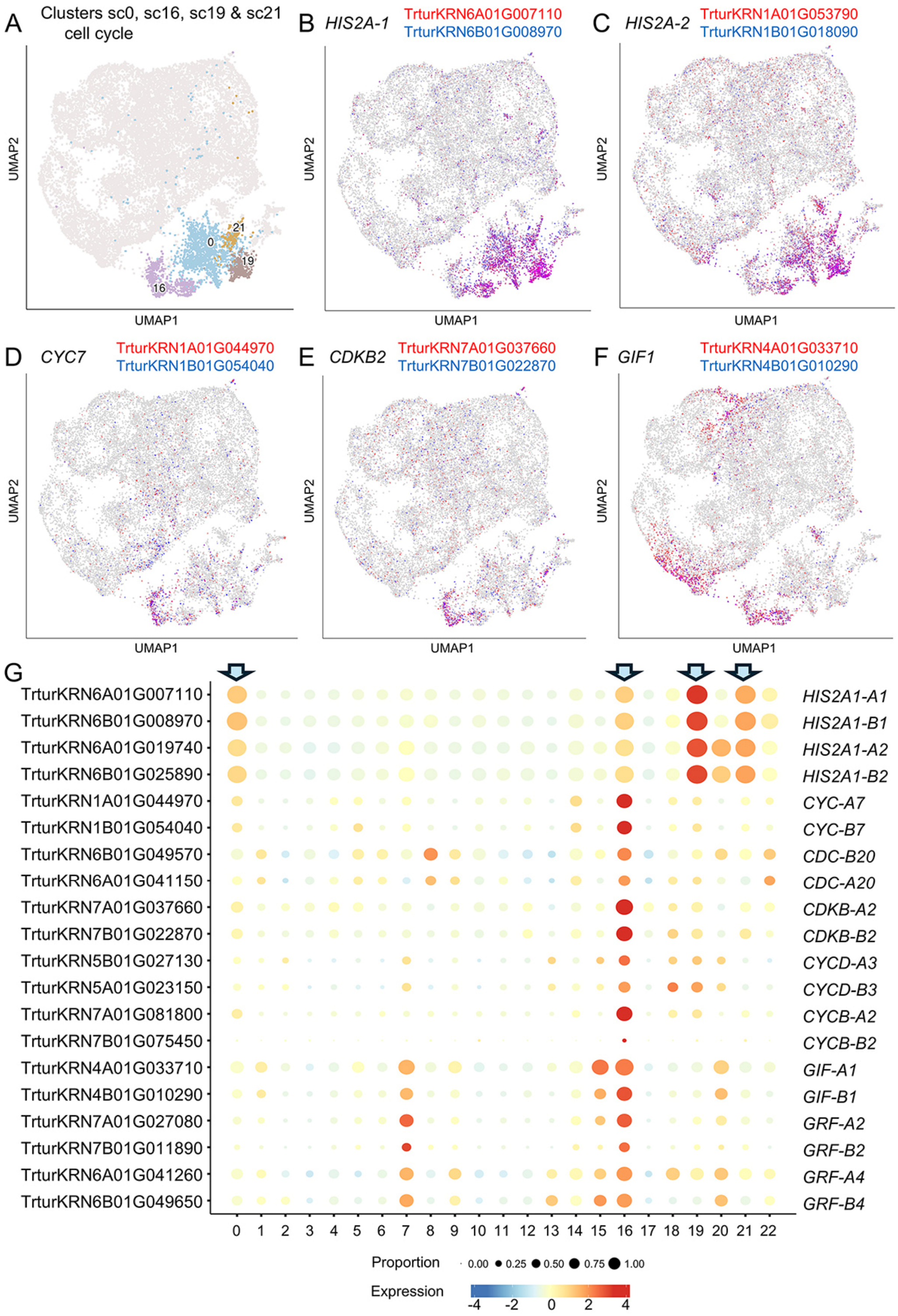

Additional file 1: Fig. S26. Known markers for single-cell epidermal clusters. A-F UMAPS and **G** bubble plots of known epidermal marker genes for clusters sc5, sc7, sc13, sc15 and sc16. Epidermal clusters showed relatively low expression of the homeodomain-box gene *OSH1*. **H** Spatial pattern of epidermal genes using imputed expression. The upper scale is the cell-fill color for the homeolog with higher expression, and the lower scale is for the cell-border color for the homeolog with lower expression. Annotation and references for marker genes are available in Additional file 2: Table S1. Additional genes preferentially expressed in epidermal cell clusters are provided in Additional file 2: Table S9.

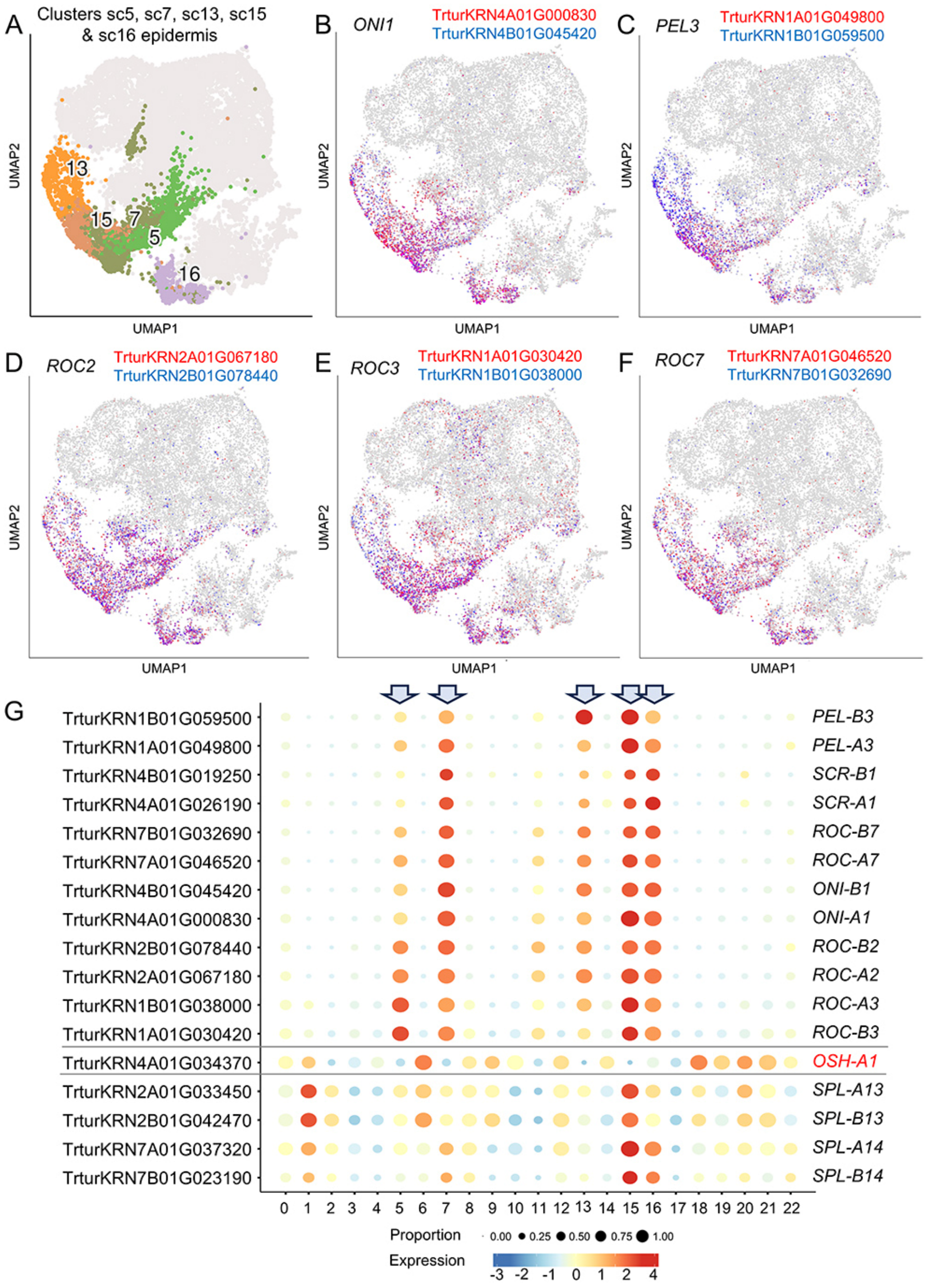

Additional file 1: Fig. S26. Continuation.

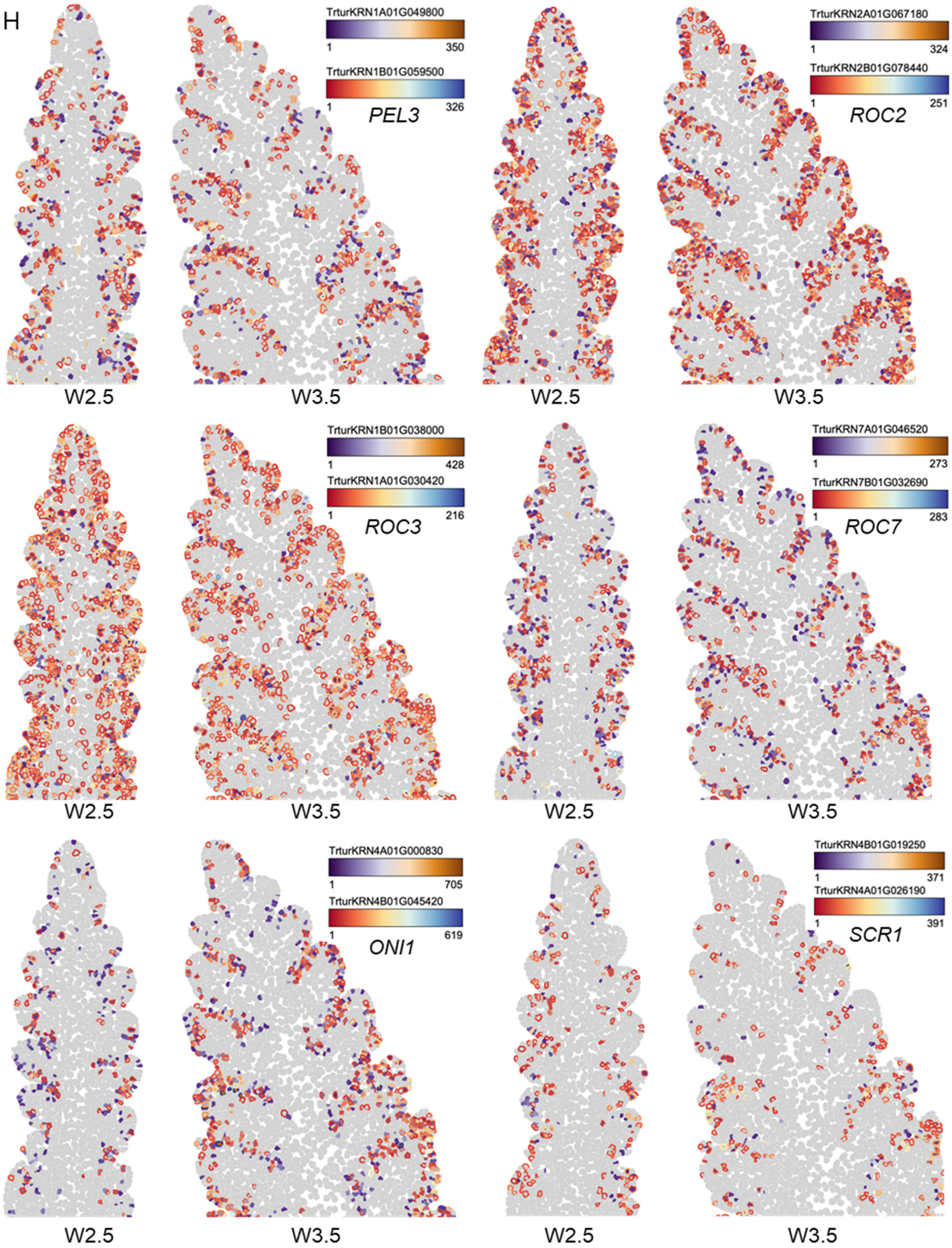

Additional file 1: Fig. S27. Differential genes among scRNA-seq epidermal clusters. A-I UMAPS of markers that differentiate the epidermal clusters. Annotation and references for marker genes are available in Additional file 2: Table S1. UMAPS for epidermal – cell-cycle cluster sc16 are provided in Additional file 1: Fig. S25. **A-C** Markers for sc7. **A** *AGL6*, **B** *WOX3b* and **C** *YAB3*. **D-E** Markers for sc15. **D** *SPL13* and **E** *SPL14*. **F-G** Markers for sc13. **F** *CUC3* and **G** *LAX1*. **H-I** Markers for sc5. **H** GSTF13 and **I** CHT6. **J-P** Imputed expression. **J** *WOX3b*. **K** *CLE33* (meristem sc7). **L** Lateral organs *YAB3* and *YAB4*. **M-P** Imputed expression of boundary genes expressed in cluster c14 and c16 at W3.5 including epidermal cells. **M** *TCP22*. **N** *CUC3*. **O** *LAX1*. **P** *DP1*. Only one homeolog is shown using a ocre-blue scale for the cell-border color. Gene identification numbers and annotations are in Additional file 2: Table S1. Additional genes preferentially expressed in the different epidermal cell clusters are in Additional file 2: Table S17.

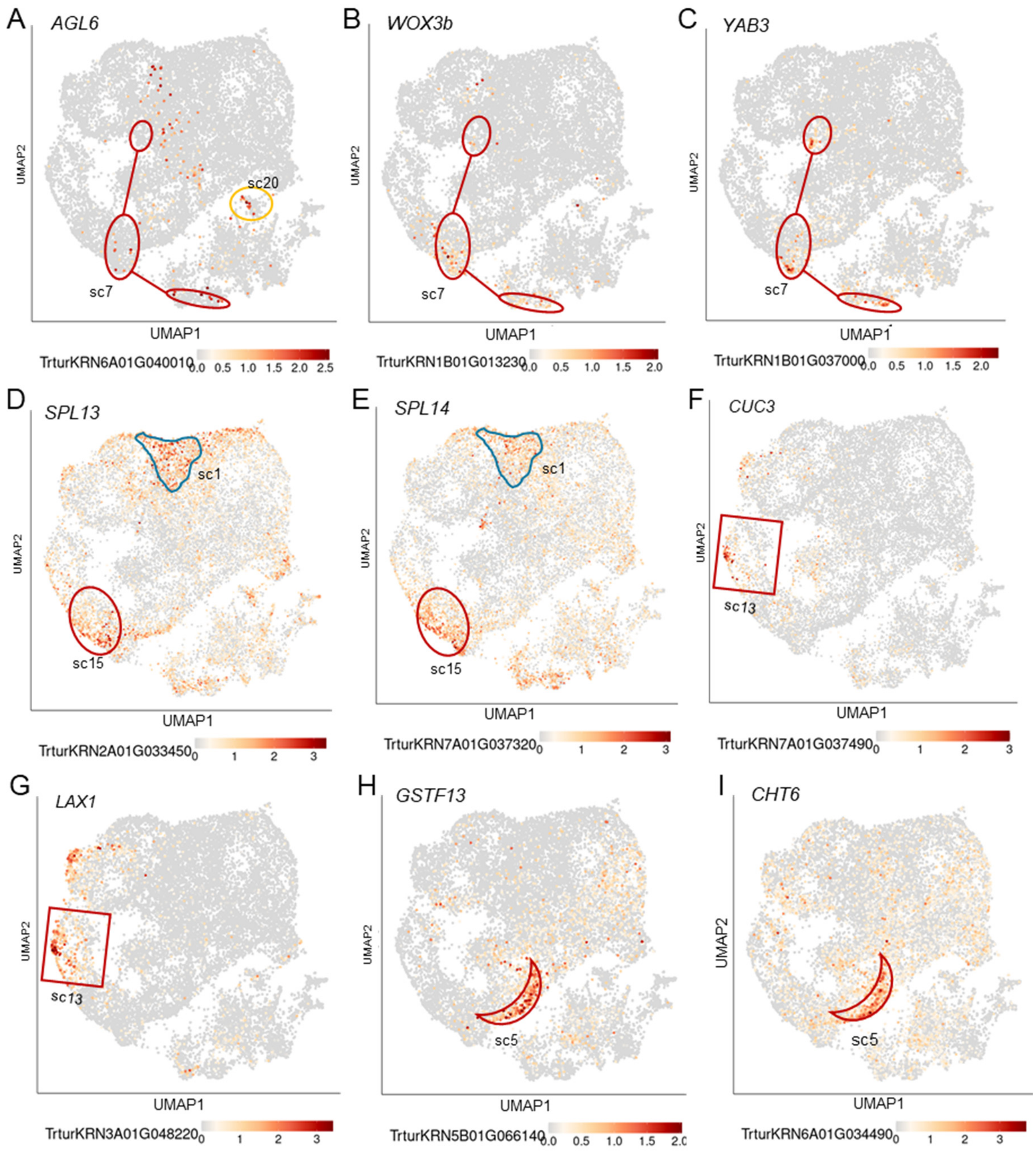

Additional file 1: Fig. S27. Continuation.

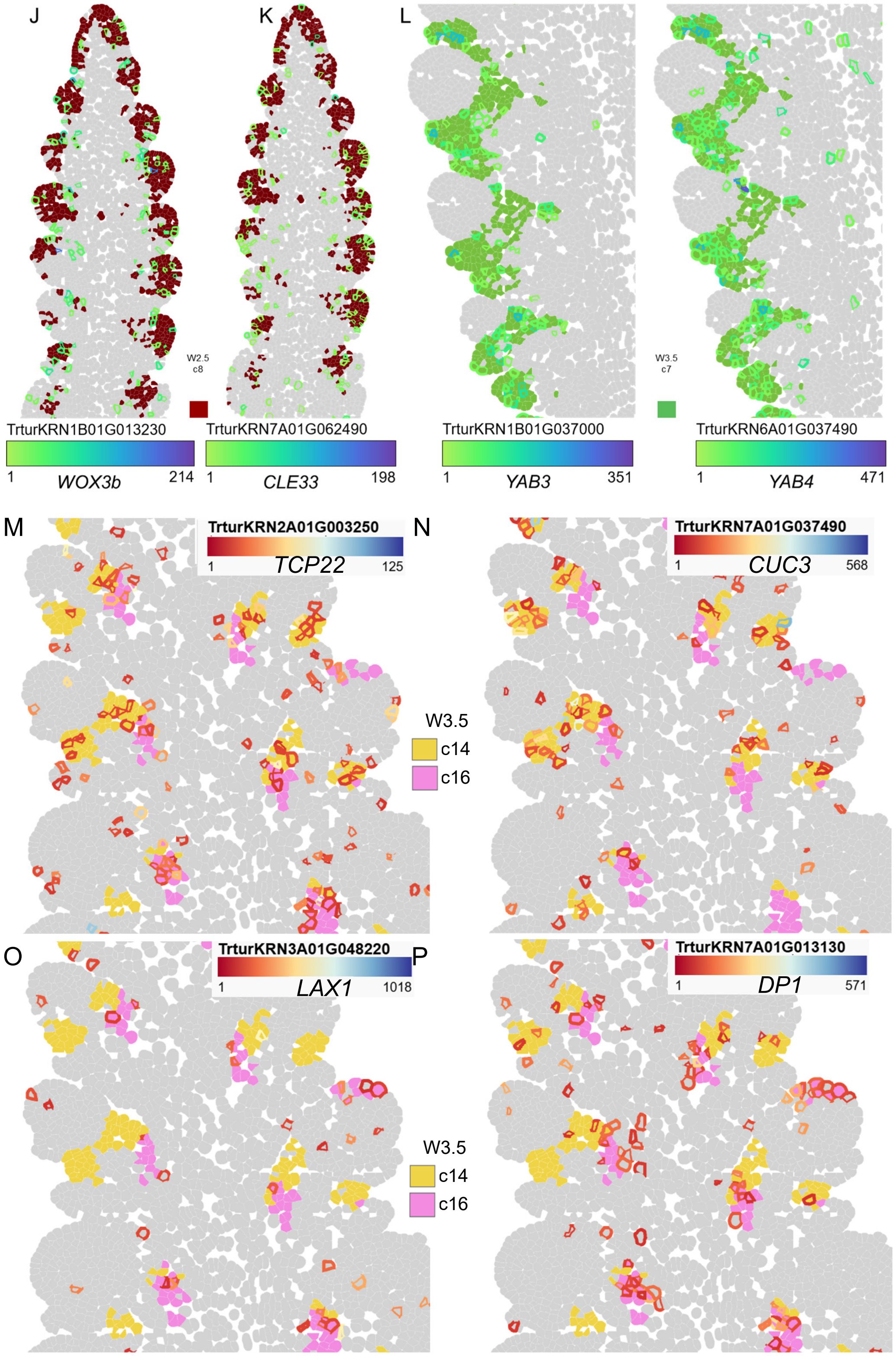

Additional file 1: Fig. S28. Known vasculature markers. A UMAPS showing cells from clusters sc18 and sc19. **B-C** Phloem markers *APL* and *JUL1*. **D** Procambium marker *LAX4*. **E-F** Xylem markers *WAT1* and *XCP1*. **G** Bubble plots of known vascular marker genes from clusters sc18 and sc19. **H-I** Spatial pattern of vascular genes using imputed expression at W3.5. The upper scale is the cell-fill color for the homeolog with higher expression, and the lower scale is for the cell-border color for the homeolog with lower expression. **H** Vascular markers (phloem marker *APL* and procambial marker *LAX4* included in the smFISH study are references). **I** Hormone-associated vascular markers. Annotation of marker genes and references are available in Additional file 2: Table S1. Additional genes preferentially expressed in vascular and pro-vascular clusters sc18 and sc19 are in Additional file 2: Table S9, and sub-clustering of sc18 in Additional file 2: Table S18. Cluster sc19 includes both cell cycle and vasculature markers.

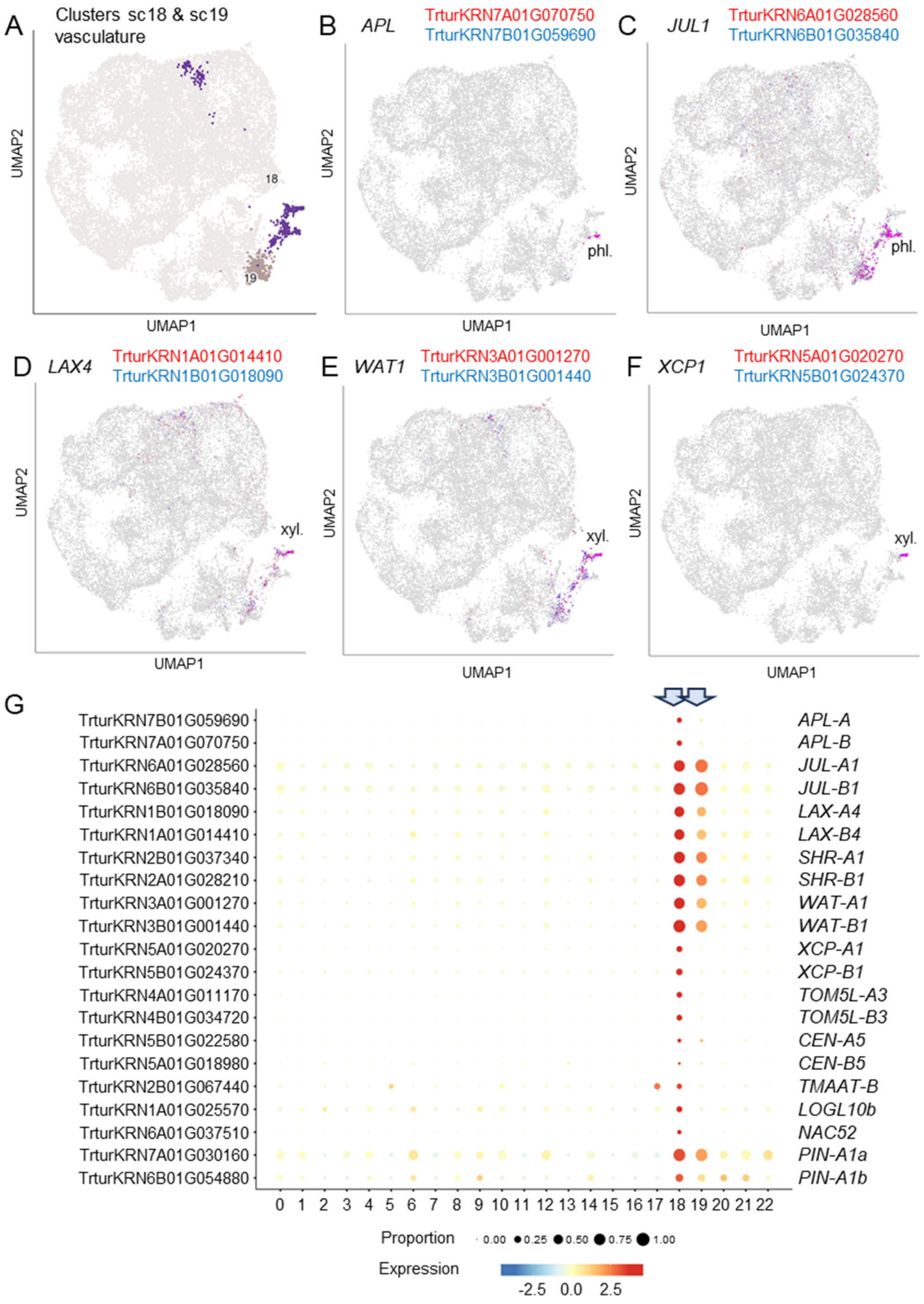

Additional file 1: Fig. S28. Continuation. Genes expressed in the vasculature at W3.5. Vasculature: *SHR1*, procambial: *LAX4*, phloem: *JUL1*, *DOF19*, and *APL*, early xylem: *WAT1*, xylem: *TMO5L1, TMO5L3*, *TMAAT*, *XCP1*, *LOG10b*, and *NAC52*.

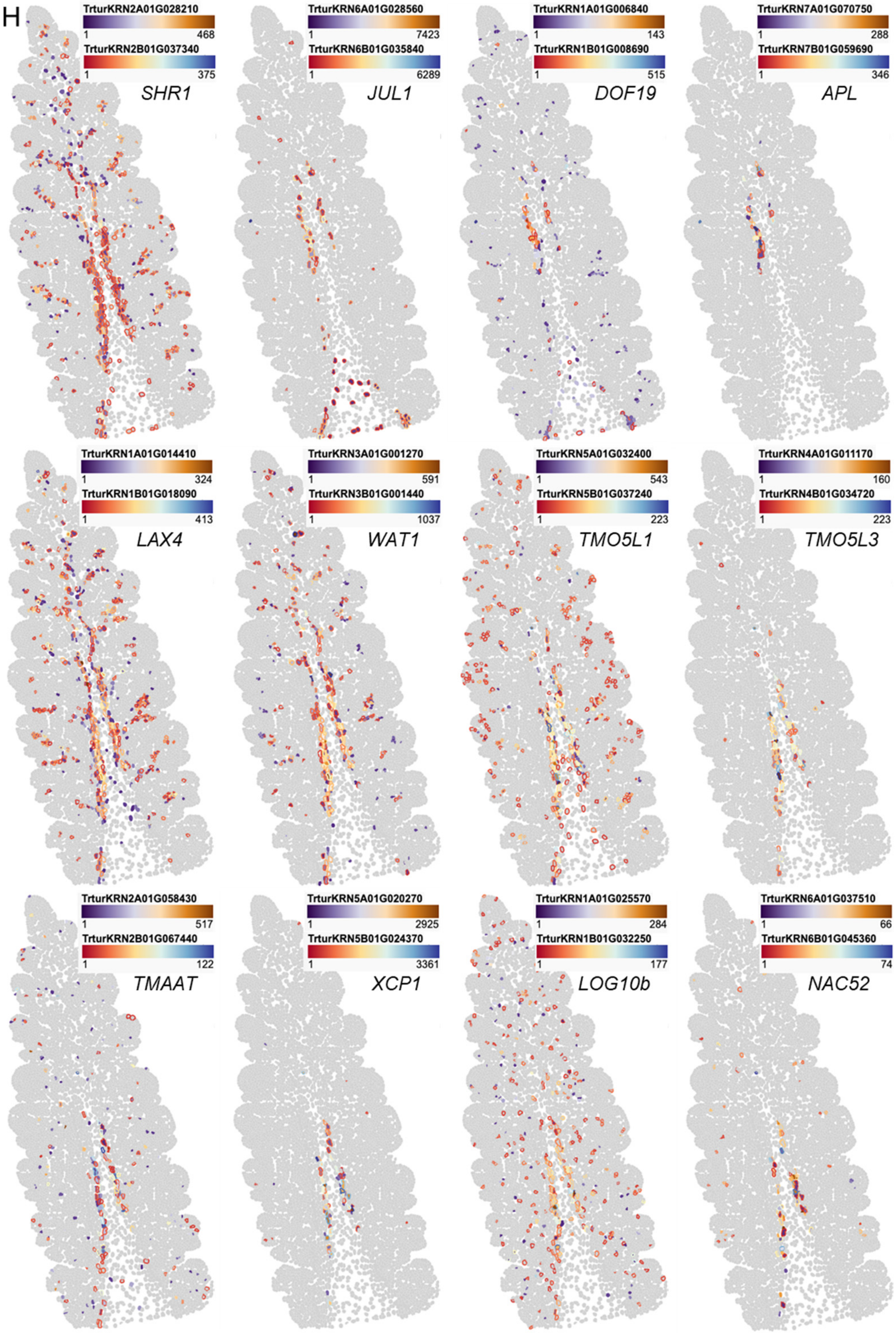

Additional file 1: Fig. S28. Continuation. Hormone-related vascular genes. Auxin: *ARF1*, jasmonic acid: *OPR3*, gibberellin: *CPS1*, cytokinin: *CKX*3, *CKX5*, *CKX11*. W3.5.

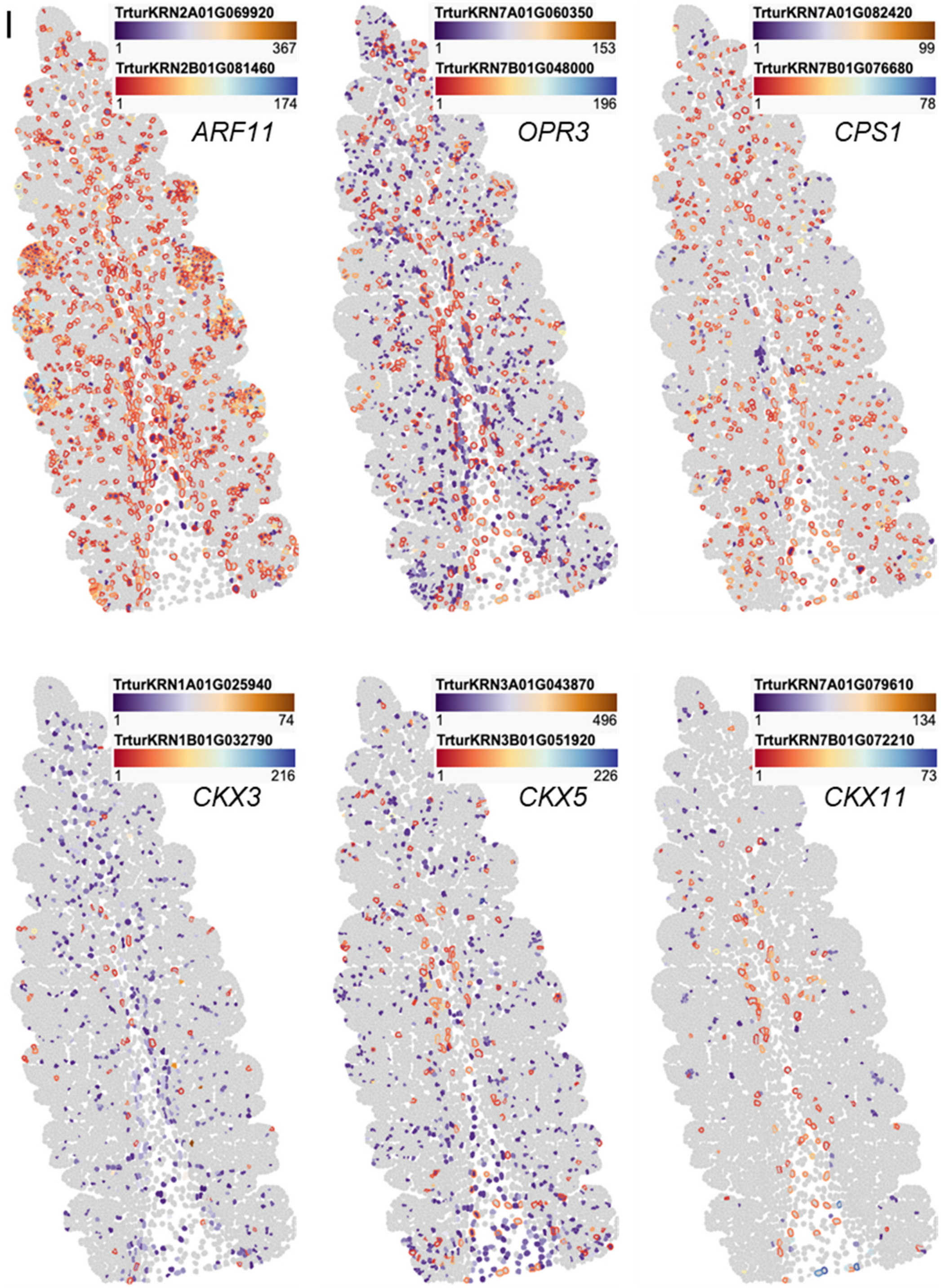

Additional file 1: Fig. S29. Co-expression of *APL* phloem marker with B-class MADS-box genes *PI1* and *AP3*. A-B UMAPS showing expression of *AP3* and *PI1* in vasculature cluster sc18. **C-E** Co-localization of *APL, AP3* and *PI1* in the vascular tissue of the leaves at W1.5 **C** and W2.5 **D**, and at the center of the spike at W3.5 **E**. Cell walls in E are stained with calcofluor-white.

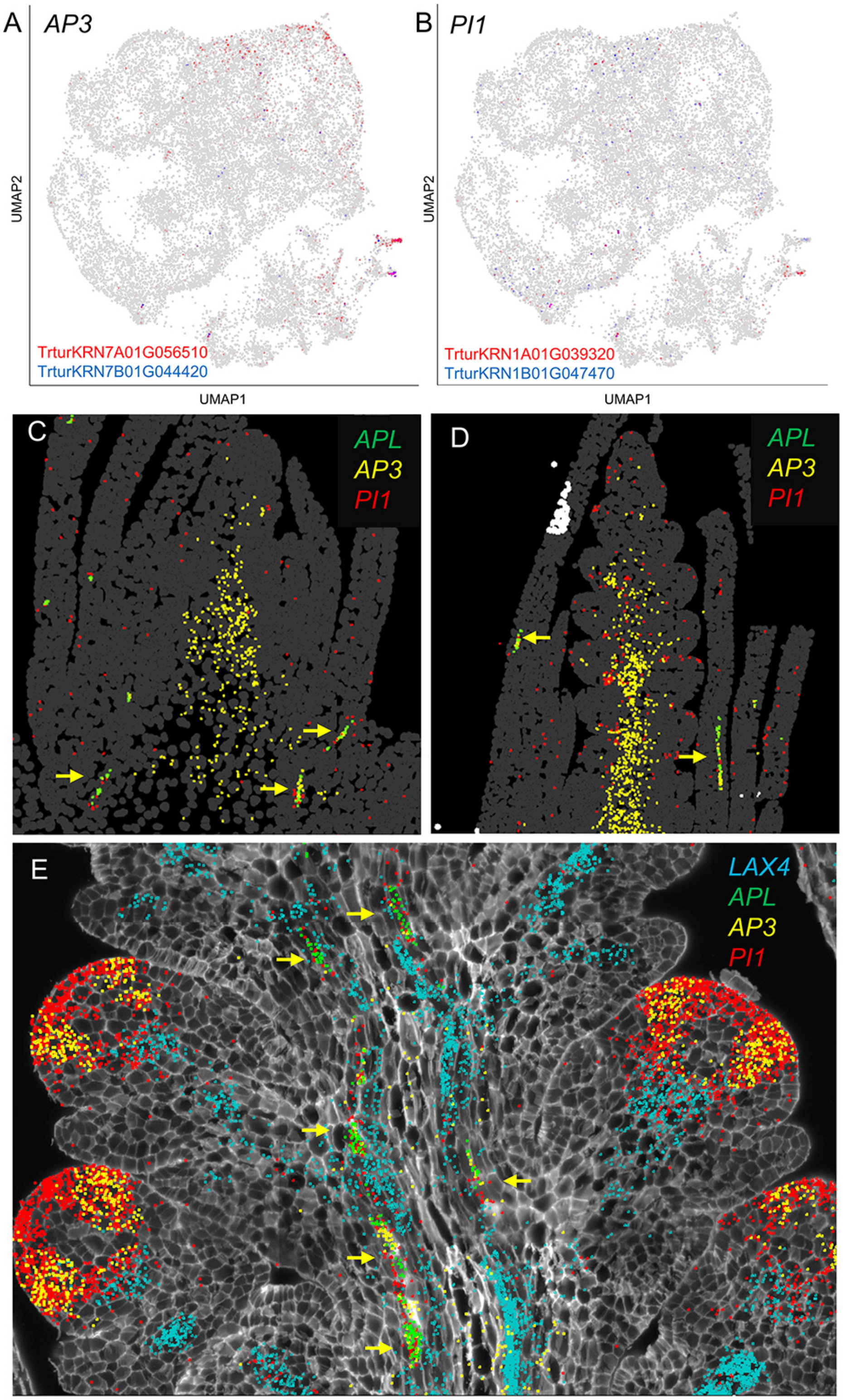

Additional file 1: Fig. S30. Genes expressed in the central spike photosynthetic ground tissue. A-F UMAPS and **G** bubble plots of known central spike ground tissue (scRNA-seq cluster sc6). **H** Imputed expression of central spikelet ground tissue genes *CAP2R* and *LHCB1.1* genes expressed in smFISH cluster c3 (brown). Only one homeolog is shown using the green-blue scale for the cell-border color. Annotation of the marker genes and references are available in Additional file 2: Table S1. Additional genes preferentially expressed in cluster c6 are provided in Additional file 2: Table S9.

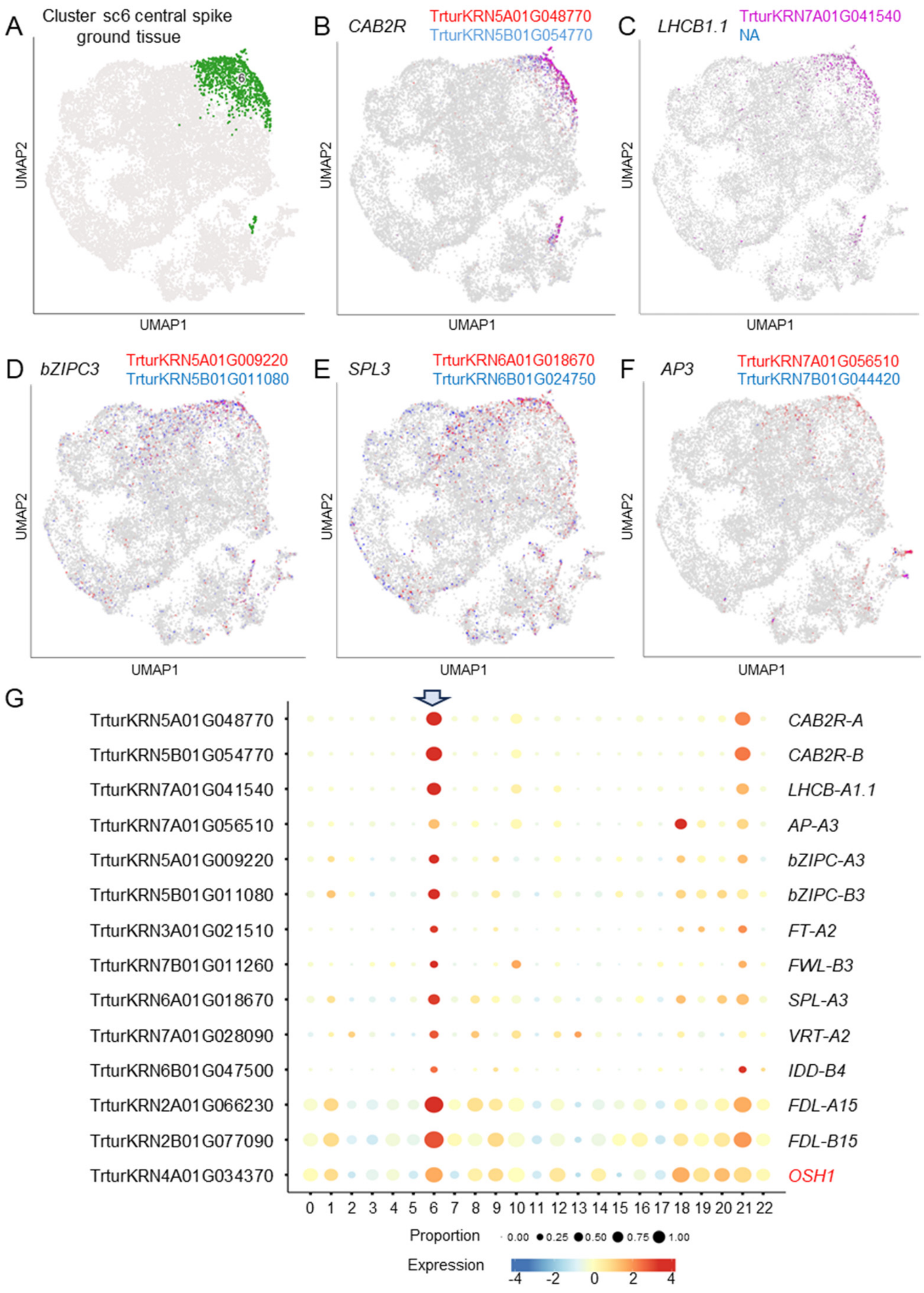

Additional file 1: Fig. S30. Continuation.

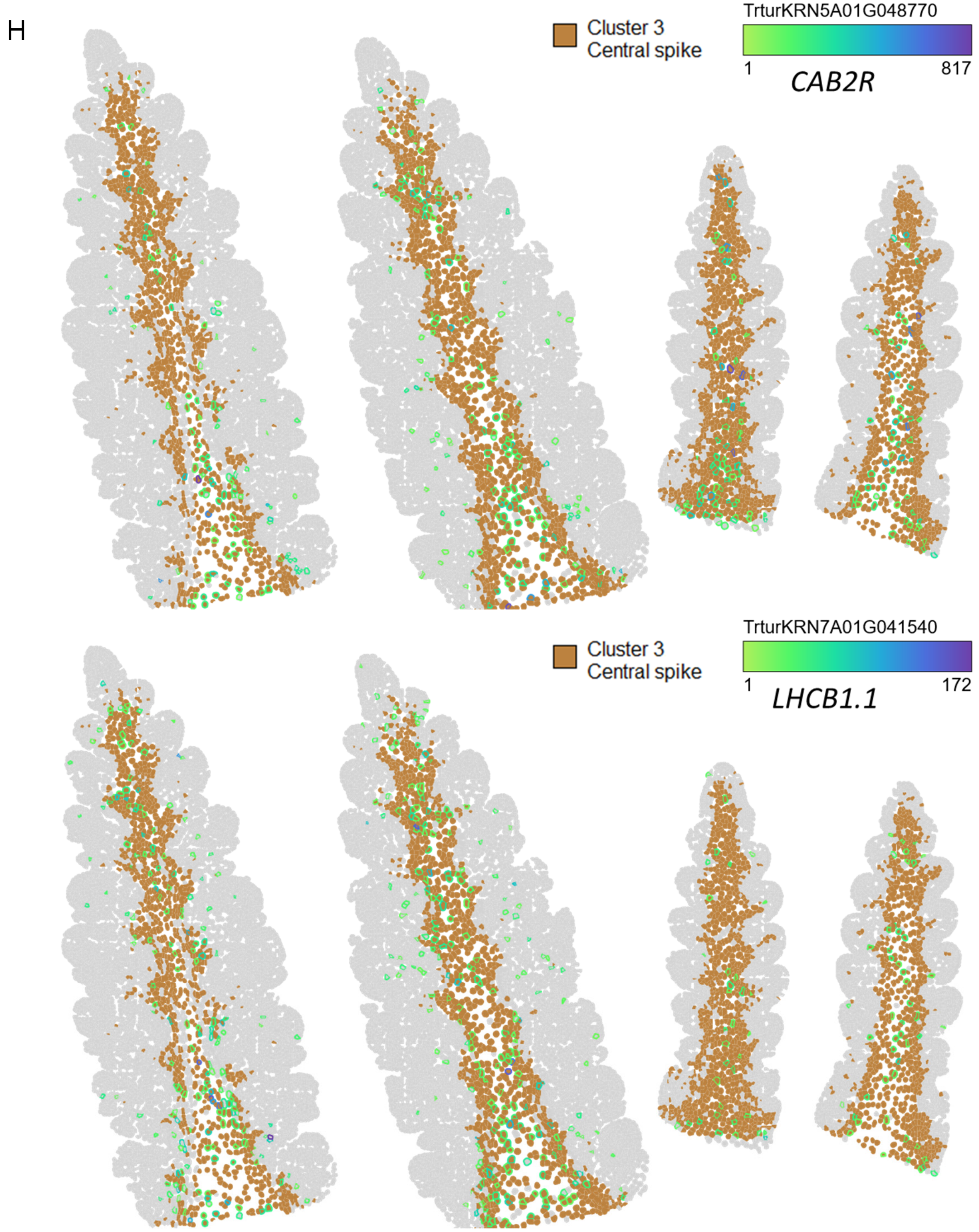

Additional file 1: Fig. S31. Heat map for genes with photosynthesis-related annotations. These genes were selected from the highest preferentially expressed genes in sc6 with photosynthesis related annotations to test the presence of these genes in other clusters. Cluster sc21 was enriched in both cell cycle and photosynthetic markers.

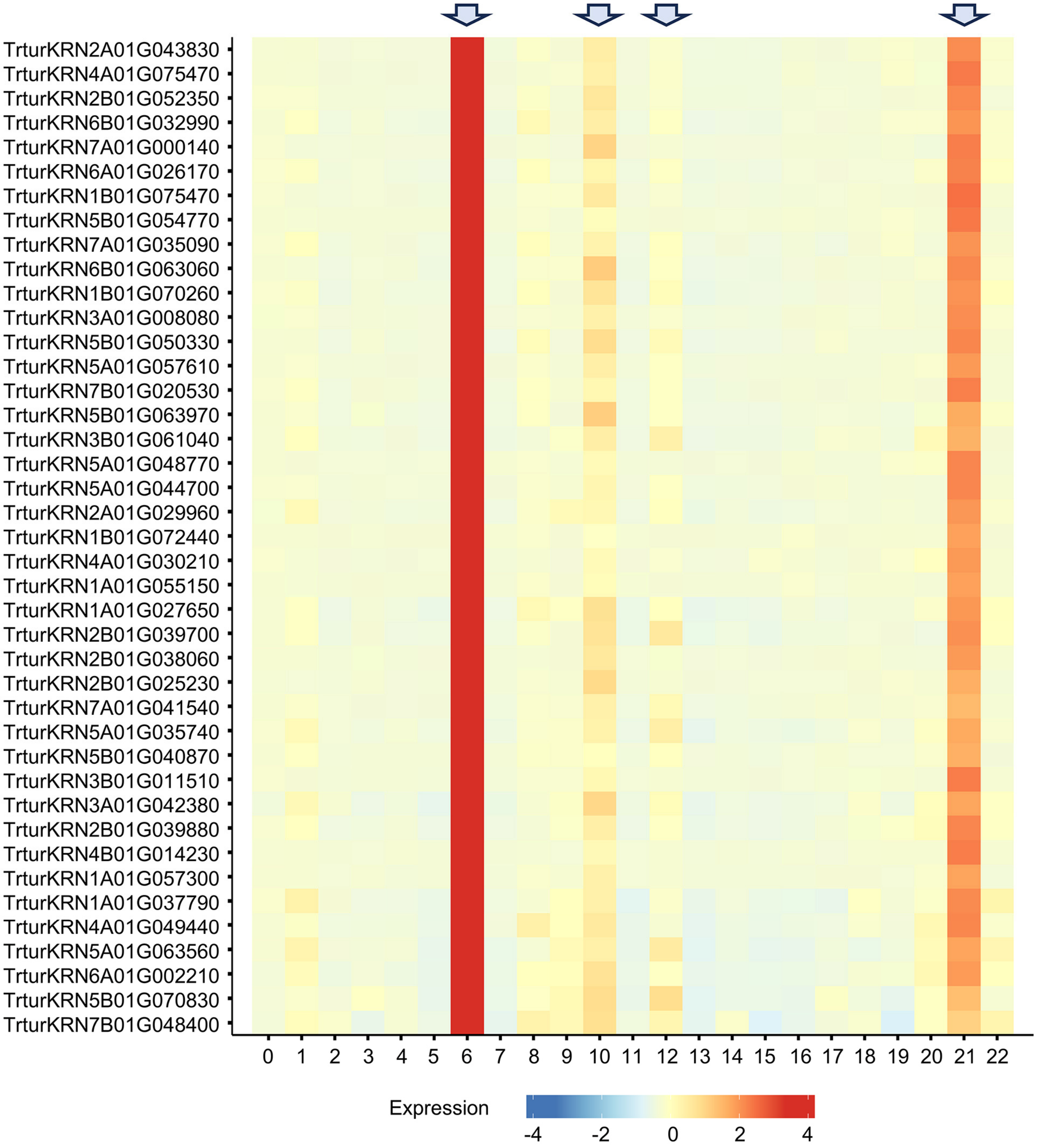

Additional file 1: Fig. S32. Genes expressed in ground tissue. A-F UMAPS and **G** bubble plots of ground tissue marker genes (sc10 and sc12). Annotation of the marker genes and references are available in Additional file 2: Table S1. Additional genes preferentially expressed in ground tissue clusters sc10 and sc12 are provided in Additional file 2: Table S9.

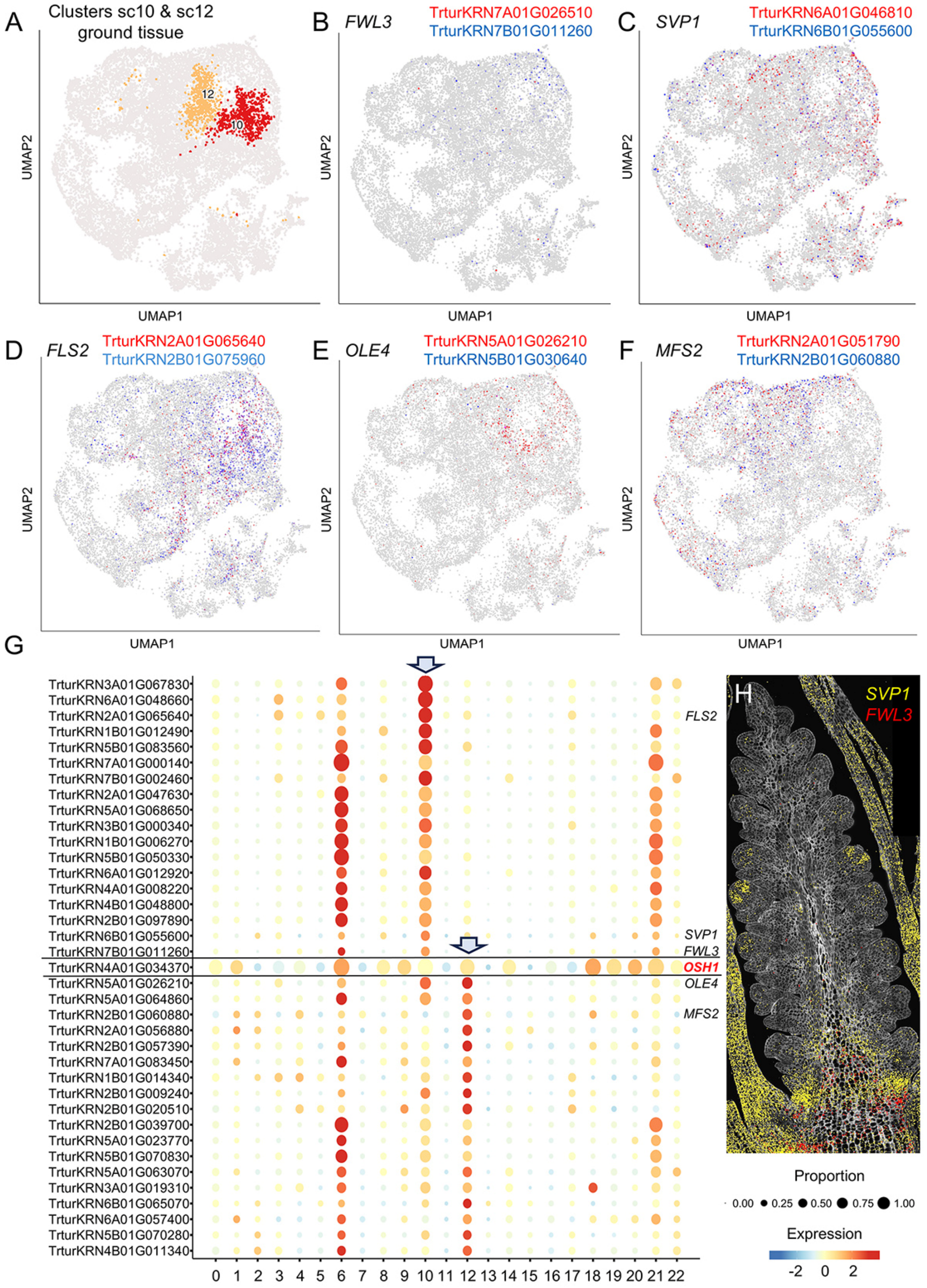

Additional file 1: Fig. S33. Genes expressed in the transition region between leaf and spike. A smFISH for genes enriched in the transition between leaves and spike at W2.5 (left) and W3.5 (right). **B-D** UMAPS and **E** bubble plots of marker genes for the transition region between the leaves and the spike (sc8). **F** Imputed expression of transition zone genes *LEC1* and *TRD1* at W3.5, JA-related genes *MYC2* and *JAZ8*, and reference genes *TB1* and *SPL17* (smFISH cluster c19). Annotation of the marker genes and references are available in Additional file 2: Table S1. Additional genes preferentially expressed in cluster sc8 are provided in Additional file 2: Table S9.

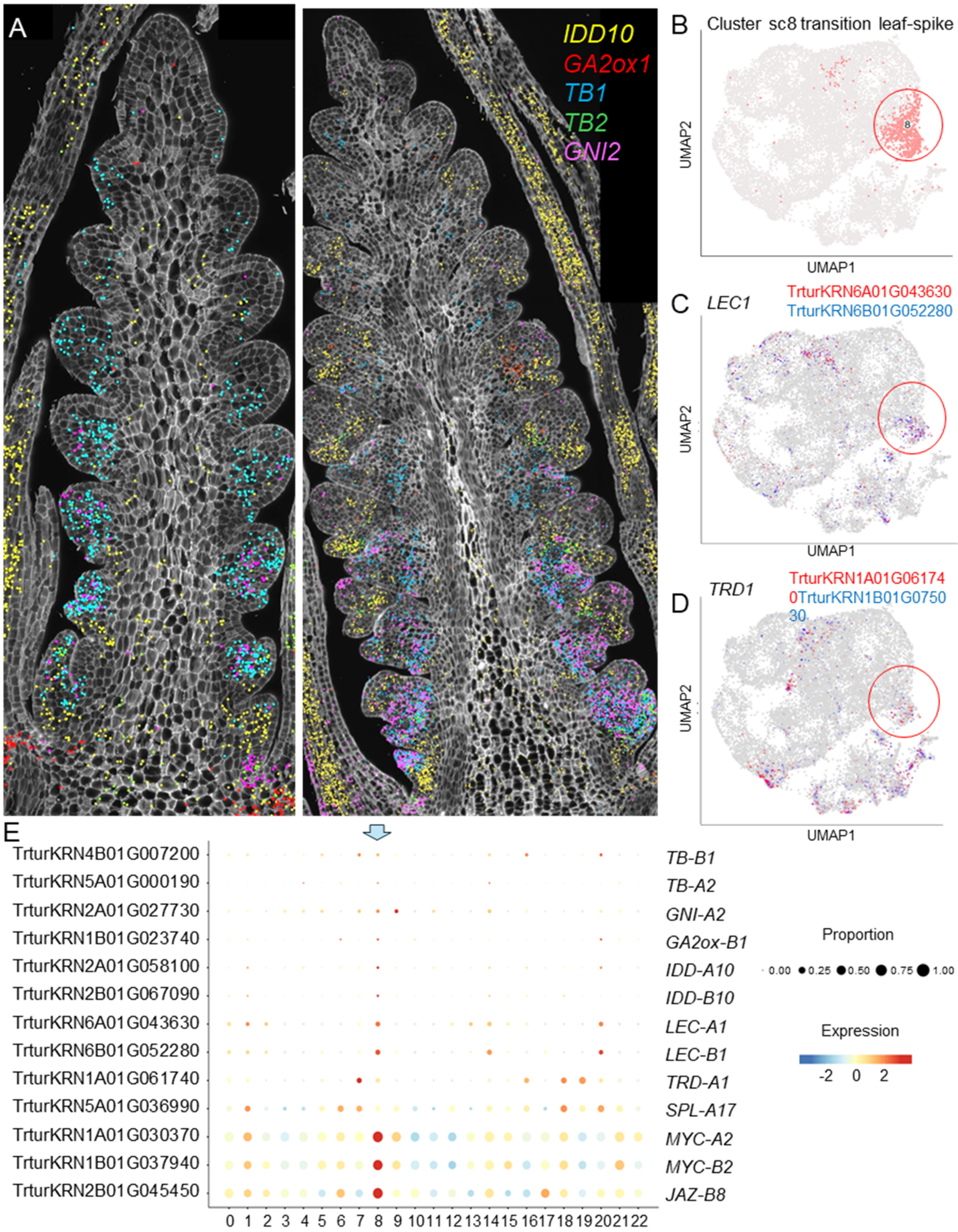

Additional file 1: Fig. S33. Continuation.

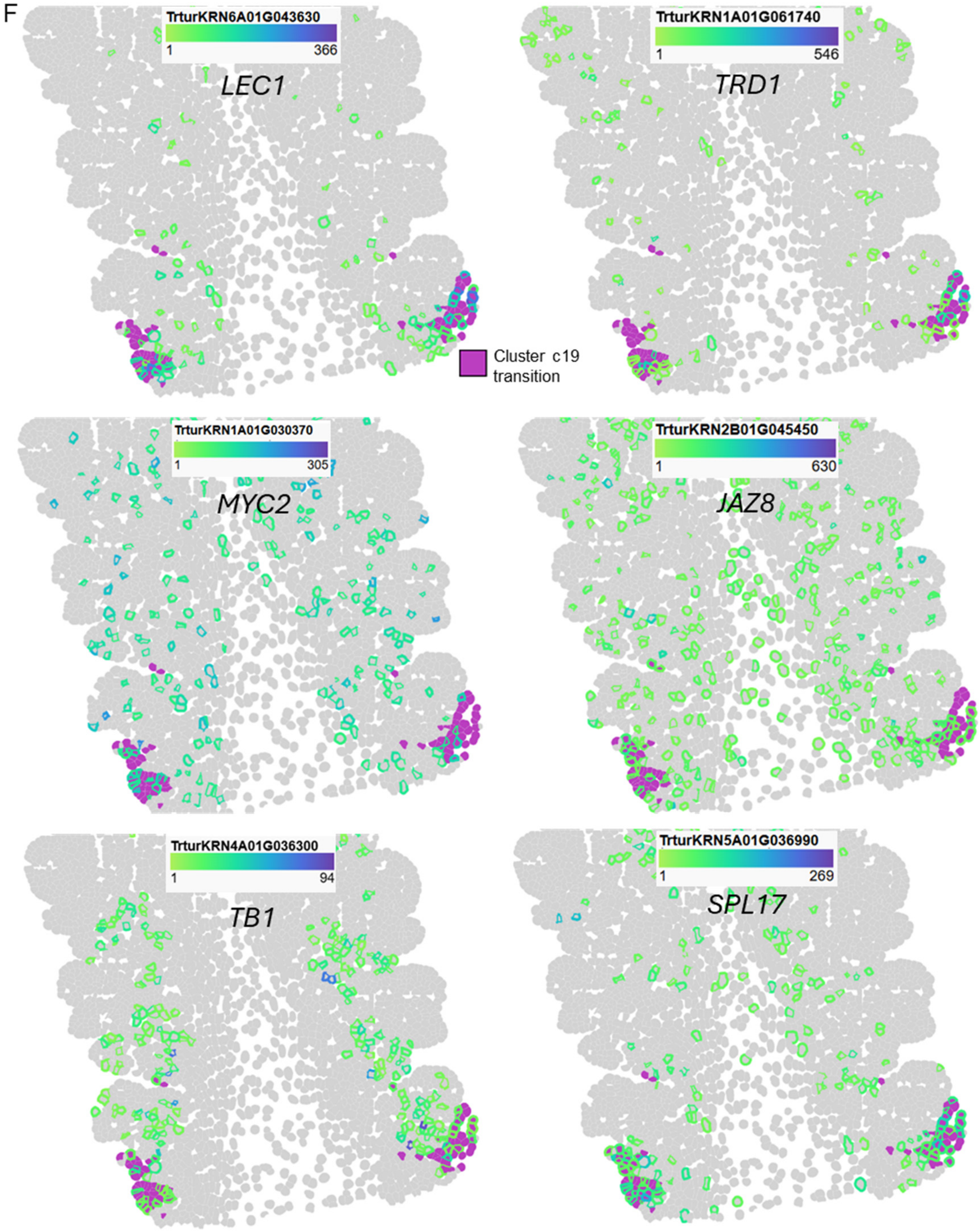

Additional file 1: Fig. S34. Genes expressed in the suppressed bract. A-F UMAPS and **G** bubble plots of marker genes for the suppressed bract cluster sc1. Annotation of the marker genes and references are available in Additional file 2: Table S1. Additional genes preferentially expressed in cluster sc1 are provided in Additional file 2: Table S9.

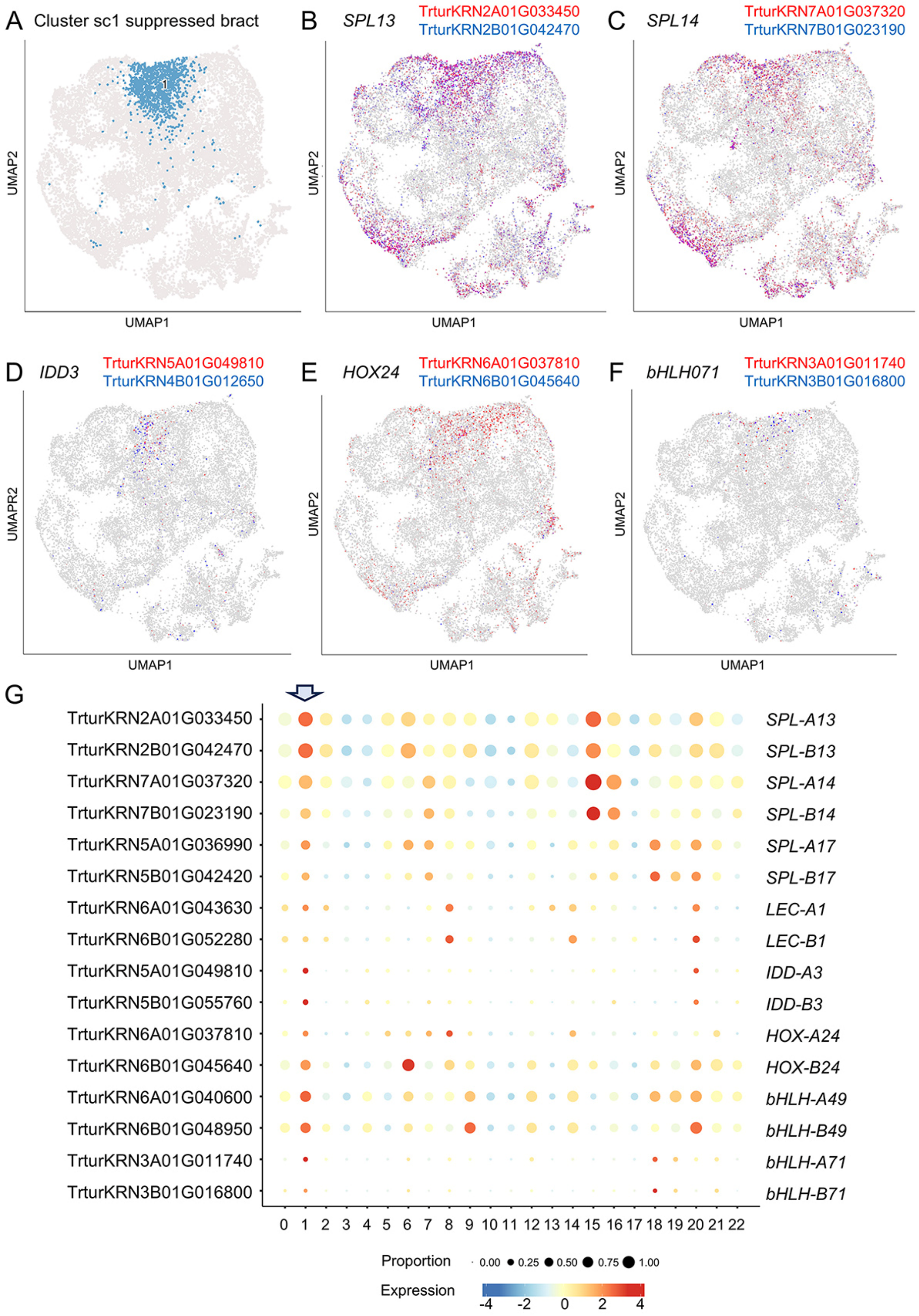

Additional file 1: Fig. S35. Validation of co-expression results using imputed expression and smFISH data. **A-B** Imputed expression of boundary genes in developing spike at W3.5 showing clusters c14and c16. Only one homeolog is shown using the green-blue scale for the cell-border color. **A** *WOX9c.* **B** *MFS1*. **C** smFISH expression of *MFS1*, *FZP*, and *TCP24* in spikelet at W3.5. **D** UMAP expression of *MFS1* in single-cell clusters. **E** *CLE33* imputed expression at W3.5 (W2.5 in Fig, S27K). **F** *RA2* imputed expression.

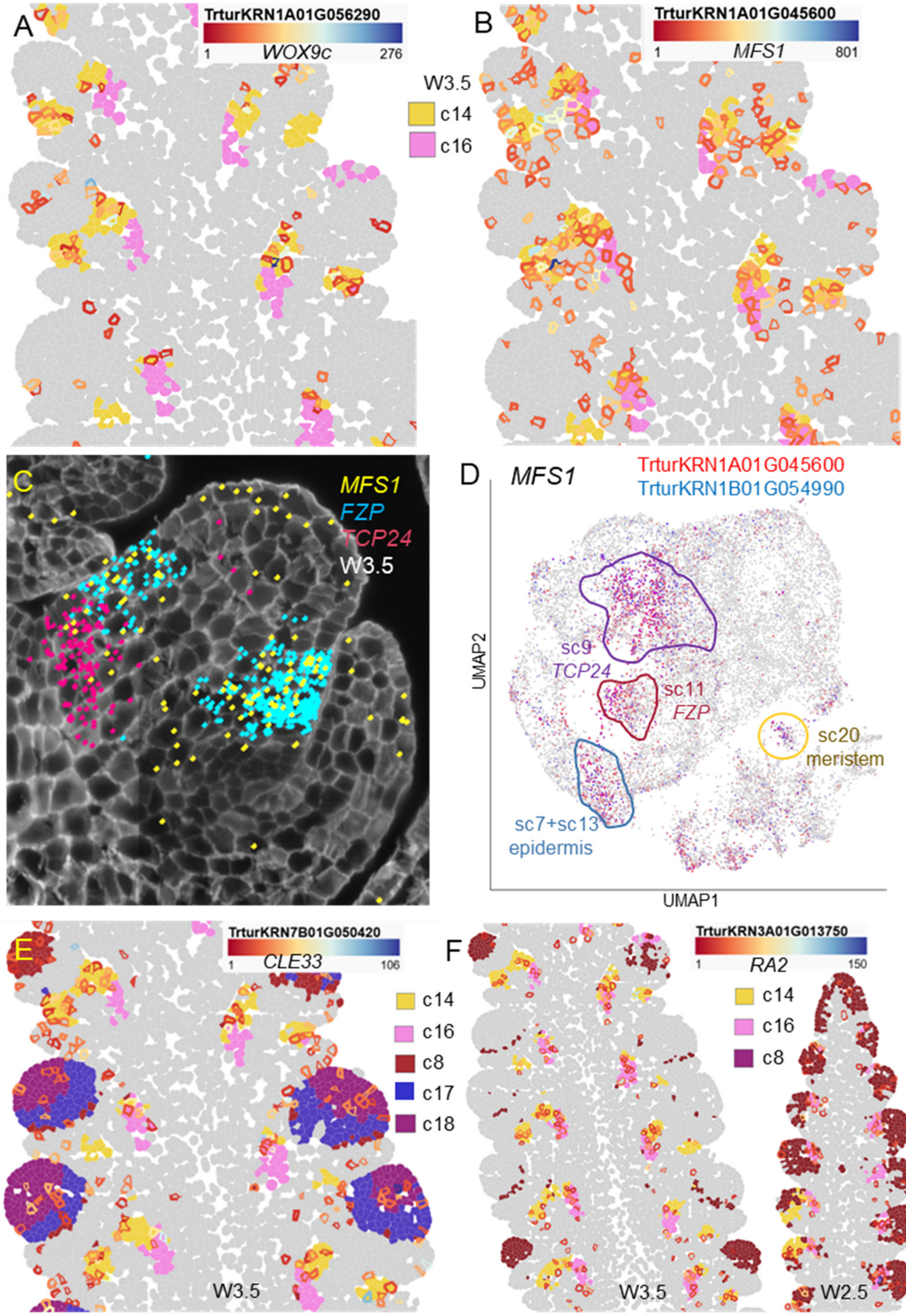

Additional file 1: Fig. S36. Cluster expressing *FZP* and co-expressed genes. A-F UMAPS and **G** bubble plots for *FZP* and co-expressed genes in the glume axilla cluster sc11. Annotation of the marker genes and references are available in Additional file 2: Table S1. Additional genes preferentially expressed in sc11 are available in Additional file 2: Table S9.

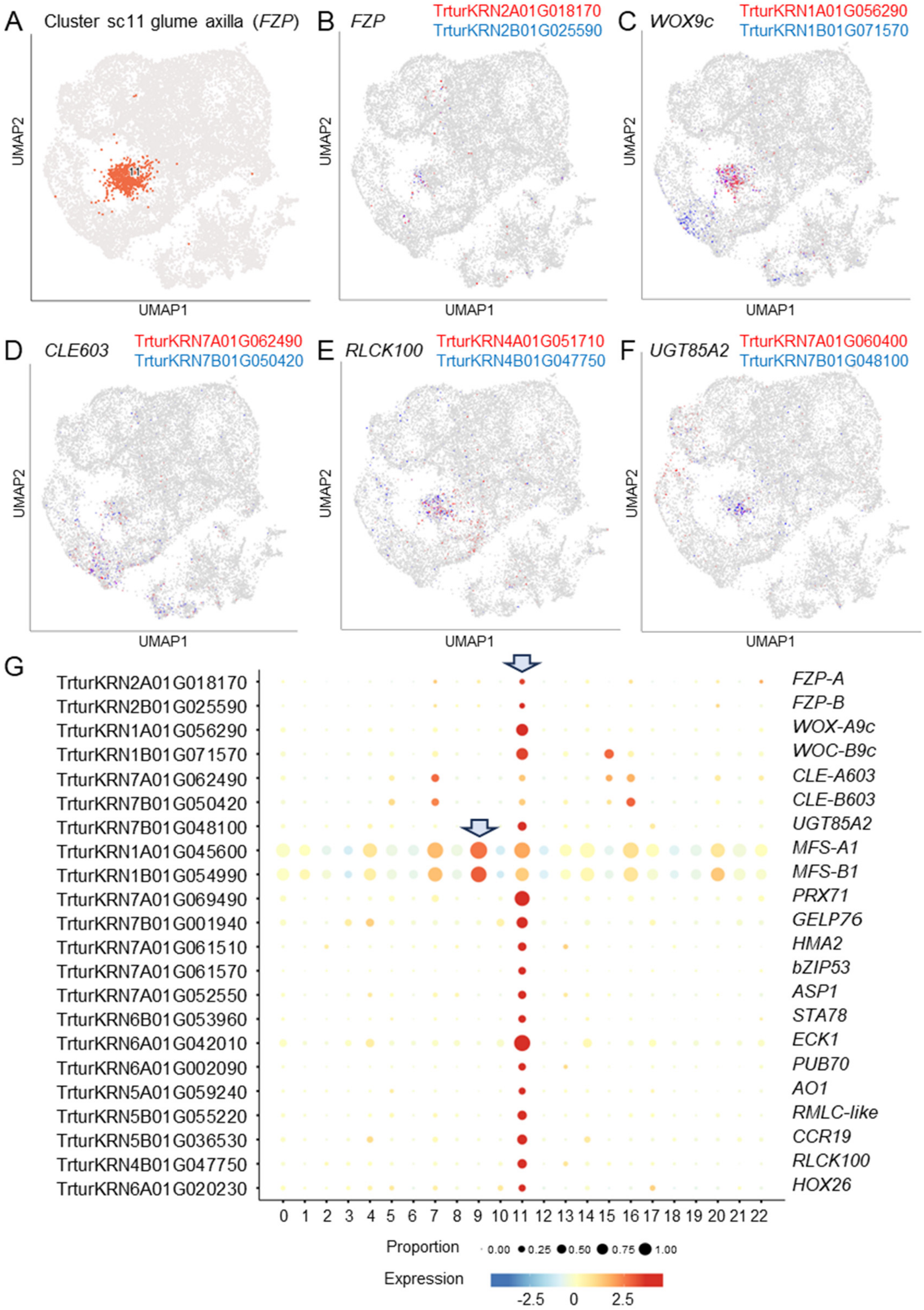

Additional file 1: Fig. S37. Clusters expressing *TCP24* and co-expressed genes. A-F UMAPS and **G** bubble plots for *TCP24* gene and co-expressed genes in the adaxial boundary clusters sc9 and sc13. Cluster sc13 also expressed epidermal markers. Annotation of the marker genes and references are available in Additional file 2: Table S1. Additional genes preferentially expressed in sc9 and sc13 are available in Additional file 2: Table S9.

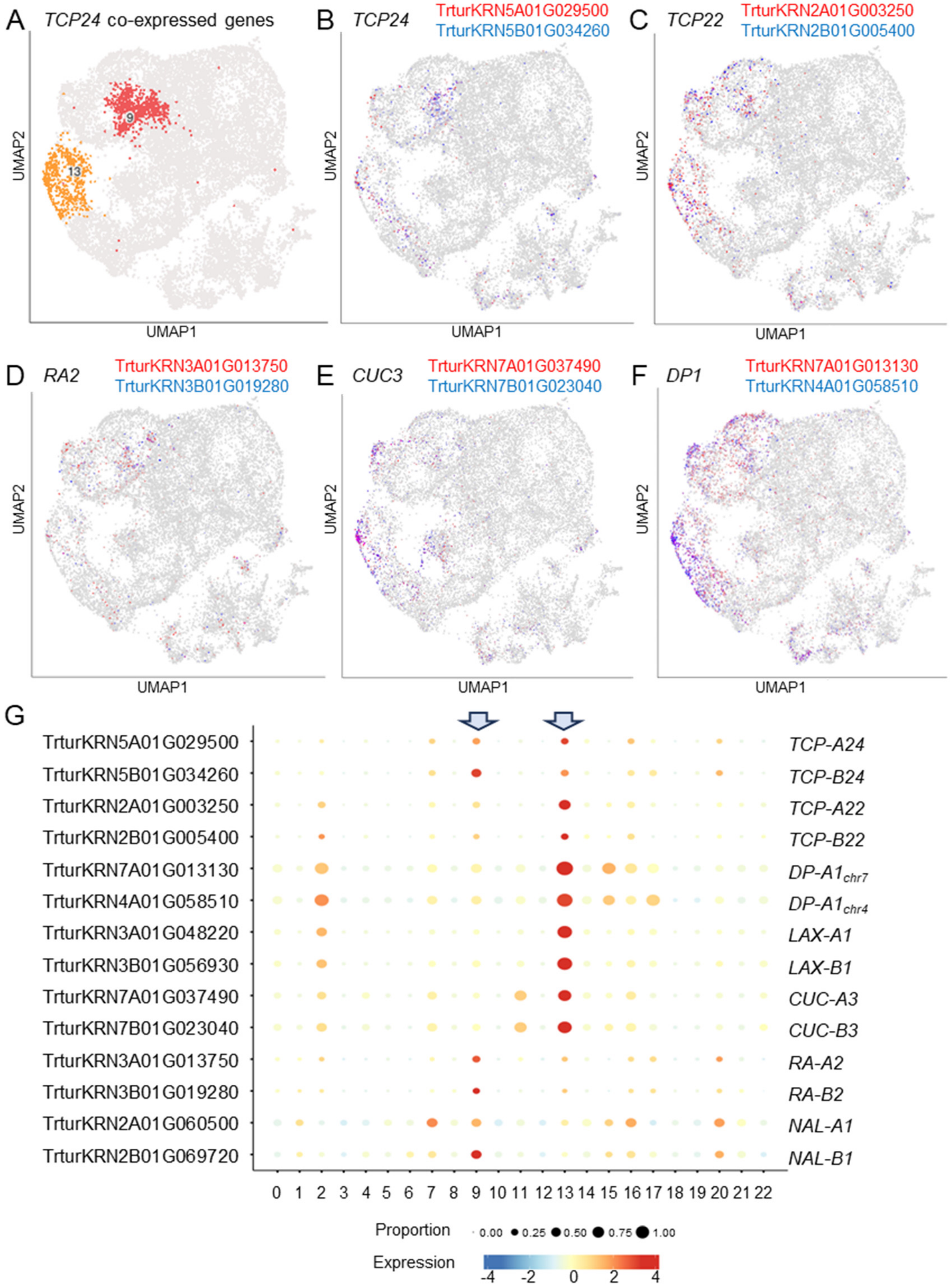

Additional file 1: Fig. S38. Genes expressed in the cortex region below developing spikelets (sc2). A-F UMAPS and **G** bubble plots of marker genes for the cortex region below the spike (sc2). **H** Spatial pattern of cortex genes using imputed expression. The upper scale is the cell-fill color for the A-genome homeolog, and the lower scale is for the cell-border color for the B-genome homeolog. Annotation of marker genes and references are in Additional file 2: Table S1. Additional genes preferentially expressed in sc2 are provided in Additional file 2: Table S9. Many of the preferentially expressed genes in sc2 and are also preferentially expressed in epidermal cluster sc13. Both clusters are highly correlated (*R* =0.96, Additional file 2: Table S8).

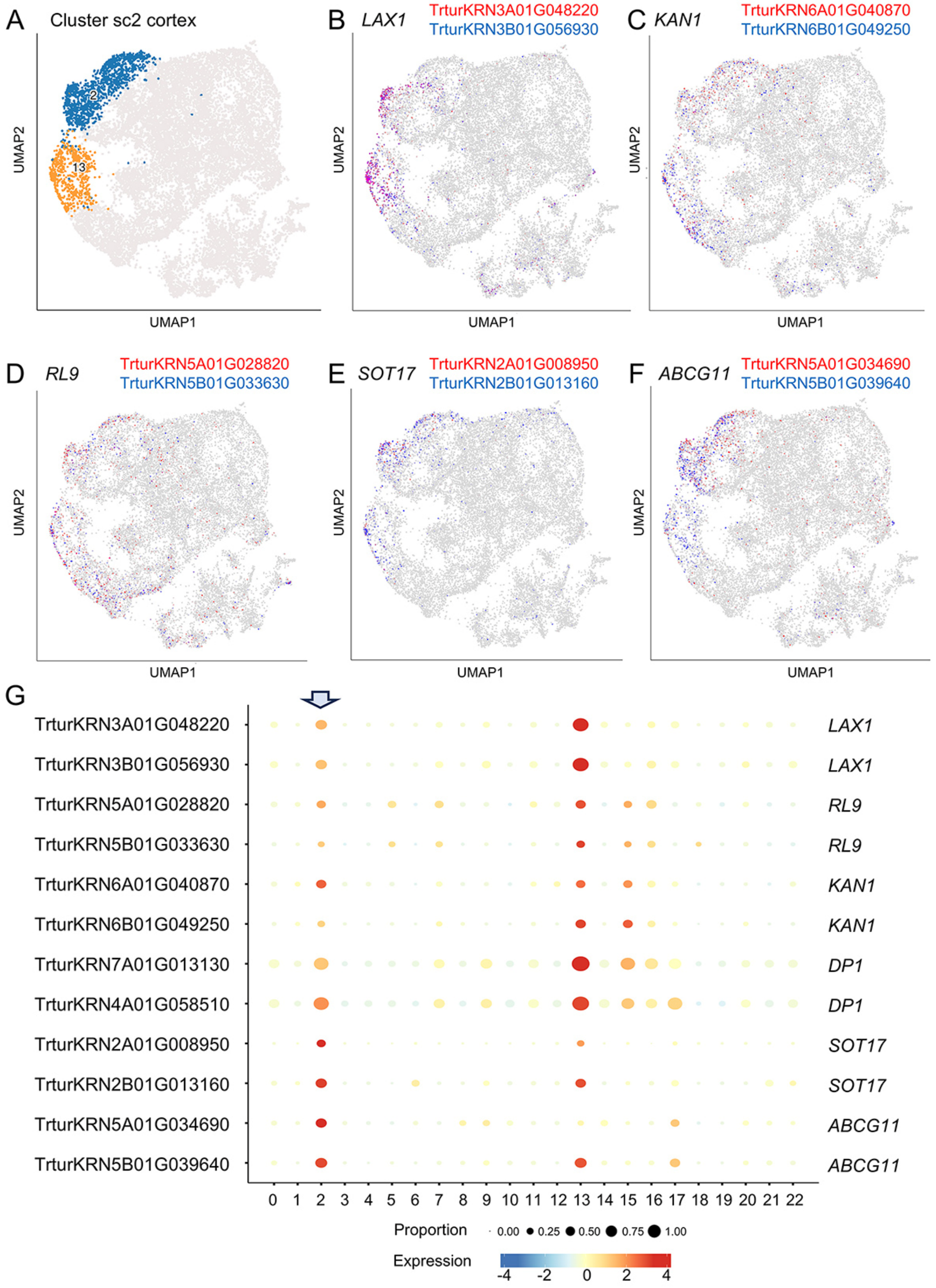

Additional file 1: Fig. S38. Continuation.

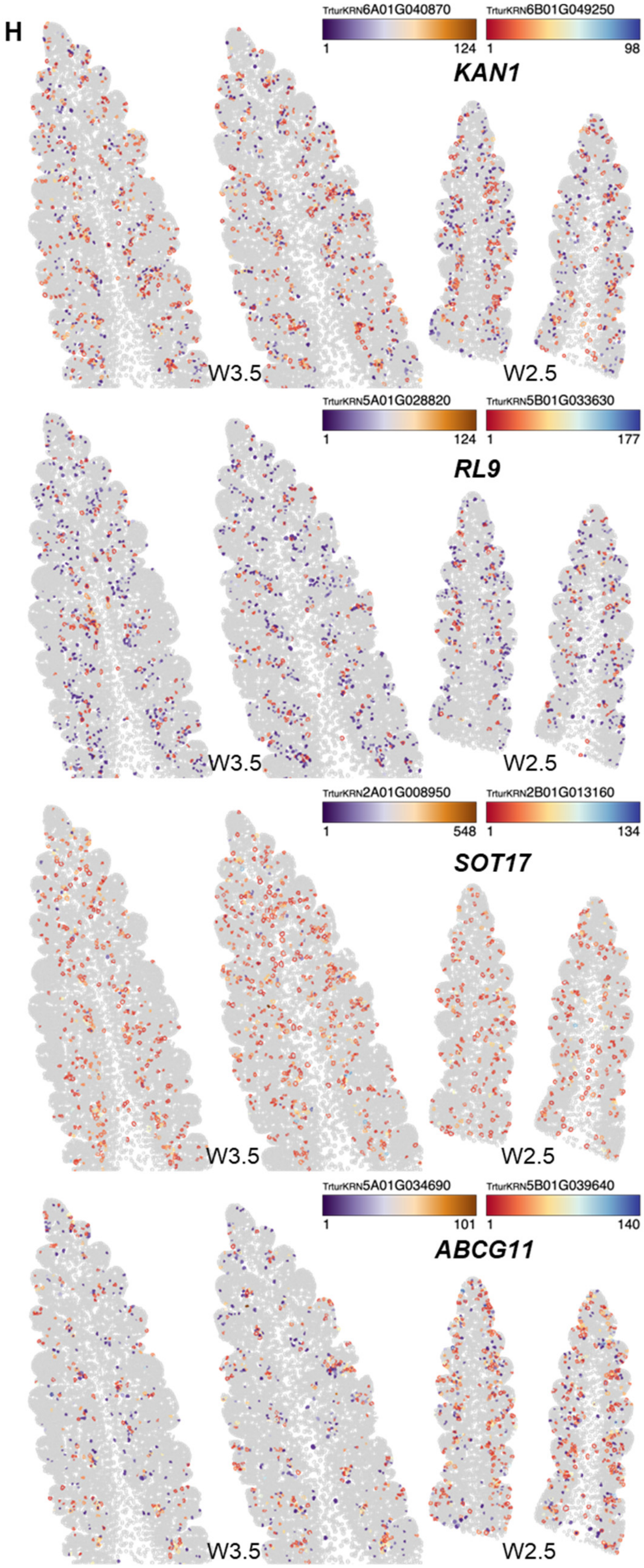

Additional file 1: Fig. S39. Genes expressed in the cortex region below developing spikelets. UMAPS and bubble plots of marker genes for the cortex region below the spike (cluster sc4 & sc17). **A** Clusters sc4 and sc17. **B-D** Examples for sc17. **E-F** Examples for sc4. **G** Bubble plot. Annotation of marker genes and references are listed in Additional file 2: Table S1. Additional genes preferentially expressed in clusters sc4 and sc17 are available in Additional file 2: Table S9.

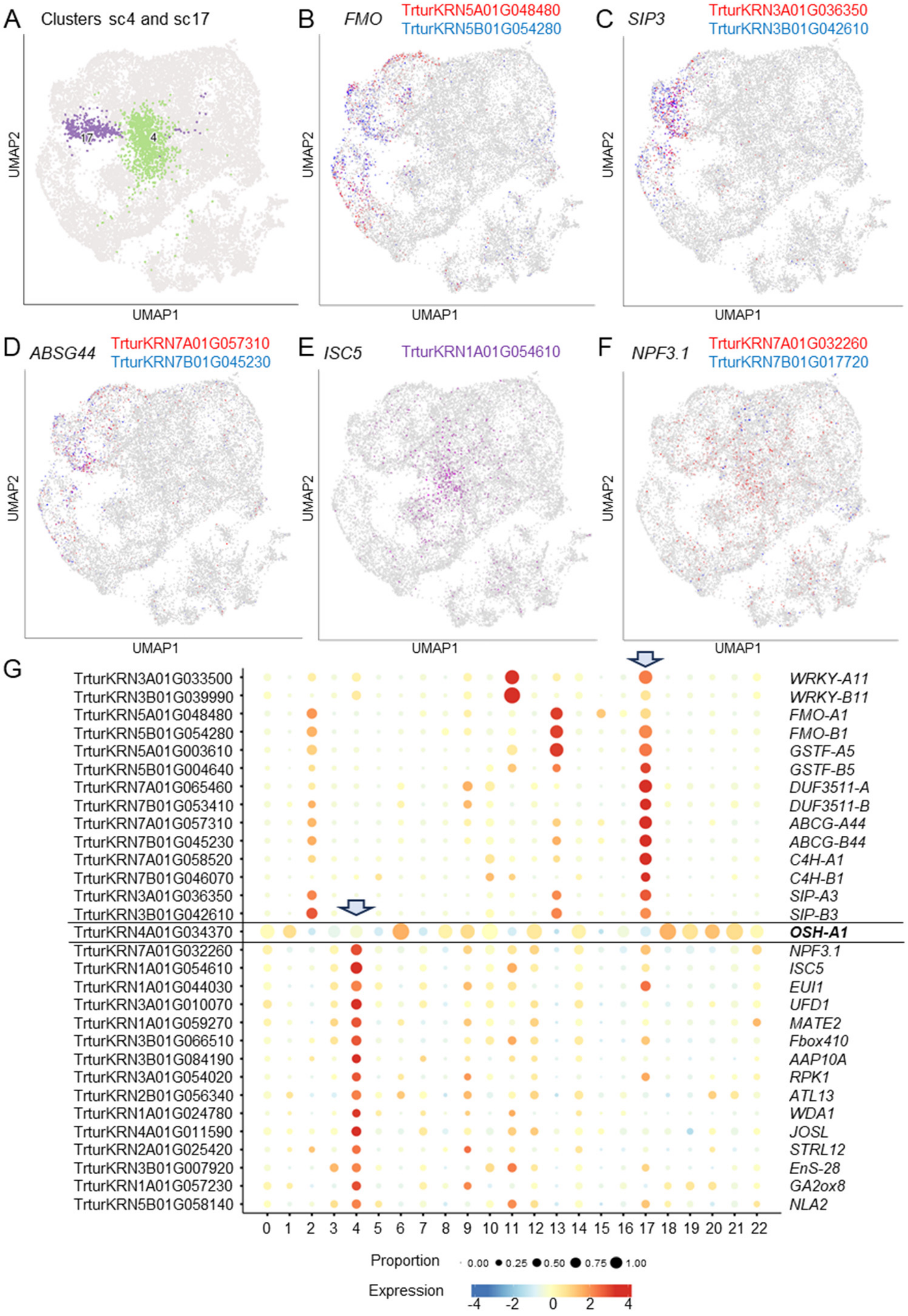

Additional file 1: Fig. S40. Genes expressed in meristems. **A-F** UMAPS and **G** bubble plots of meristematic marker genes expressed in sc14 and sc20. **H-J** Differential expression profiles of *ULTRAPETALA1* homeologs *ULT-A1* and *ULT-B1.* **H-I** UMAPS. **J-K** Imputed expression of both homeologs. **L-P** Imputed expression of additional genes expressed in meristematic clusters sc14 and sc20. In **J to P** levels of gene expression are indicated by a green-blue color expression scale for the cell-borders. Additional genes preferentially expressed in sc14 and sc20 are provided in Additional file 2: Table S9. Annotation of the marker genes and references are available in Additional file 2: Table S1.

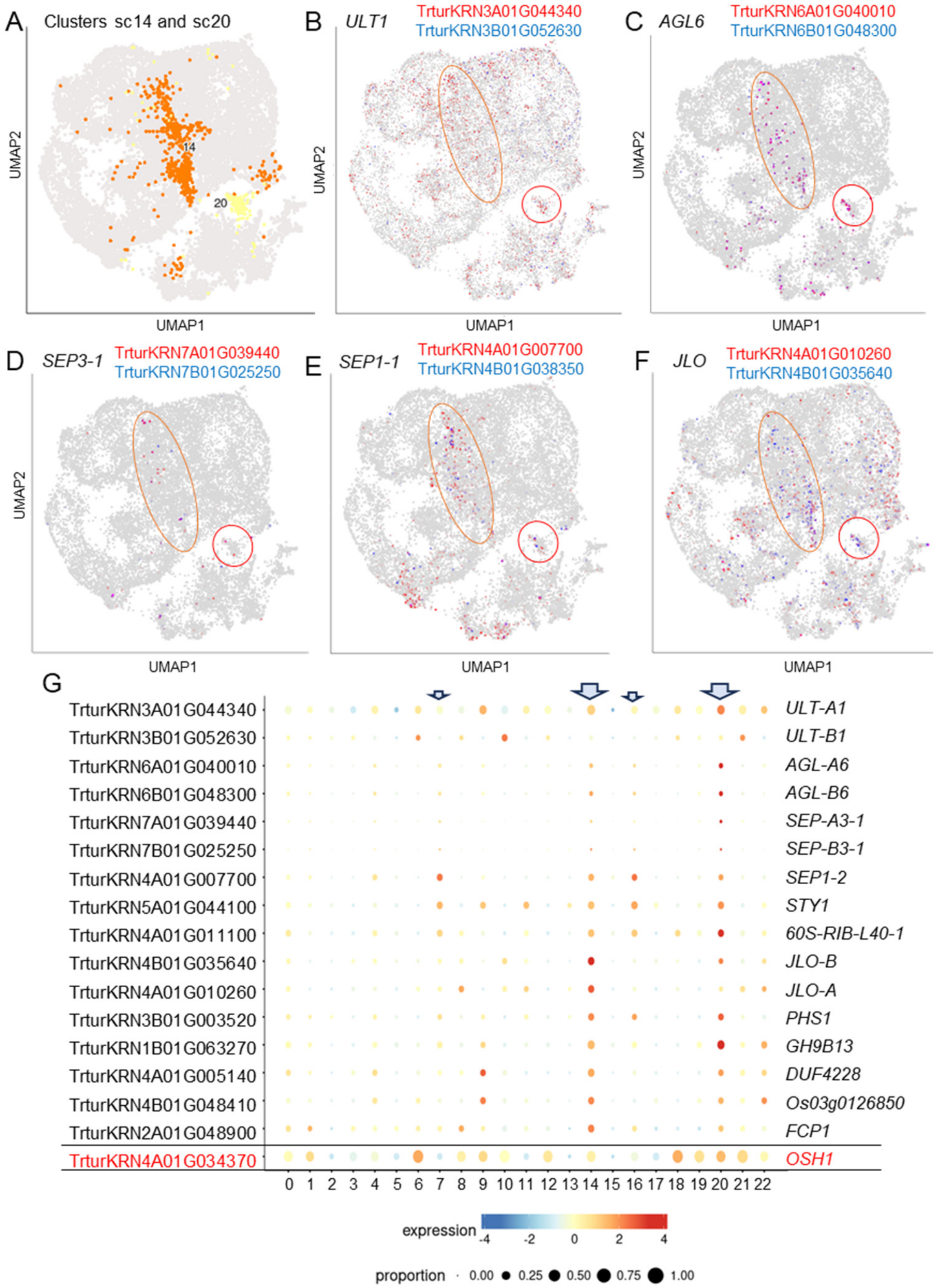

S40. Continuation. Differential expression of *ULT-A1* and *ULT-B1*

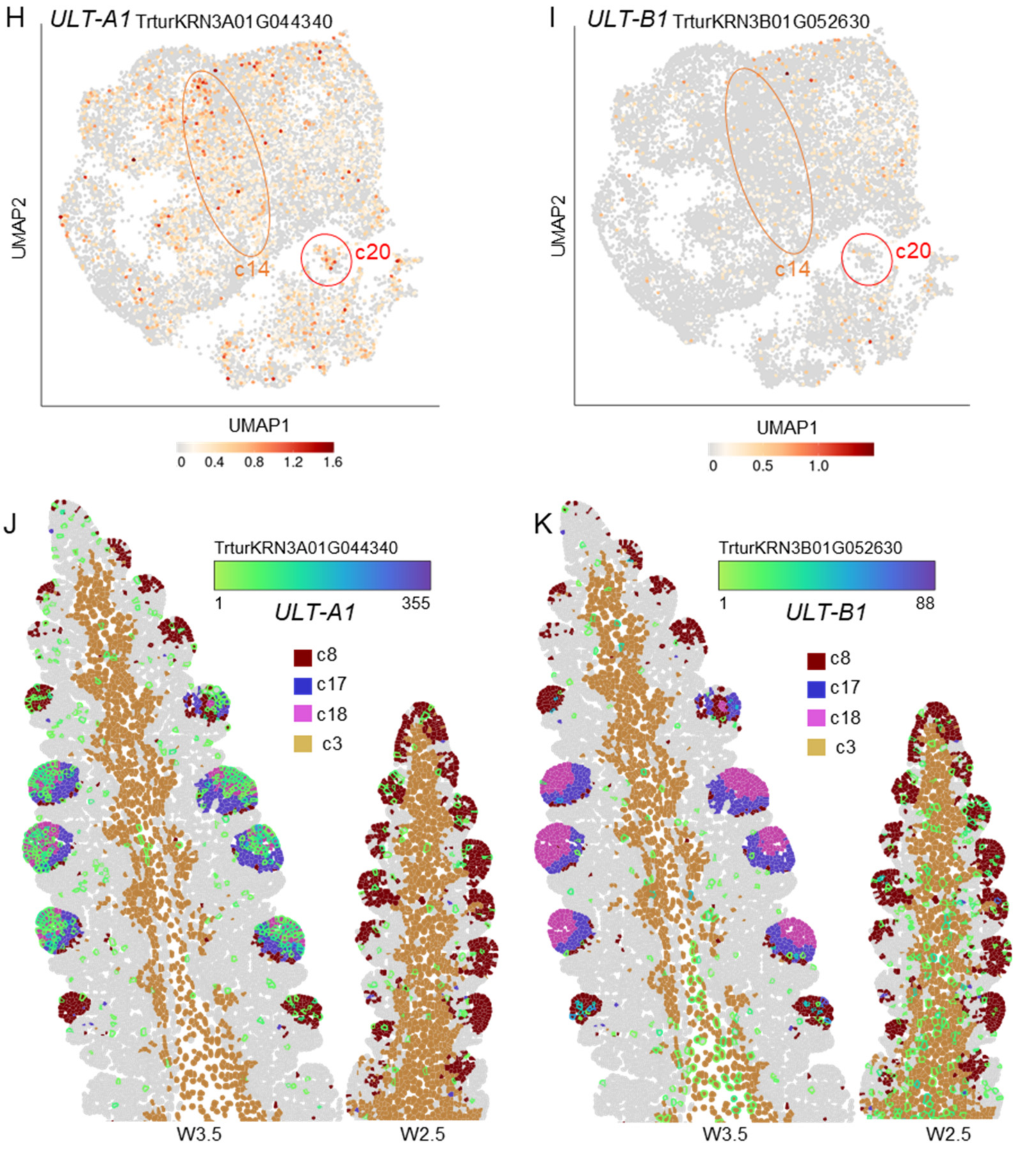

S40. Continuation. Genes preferentially expressed in meristematic clusters sc20 and sc14.

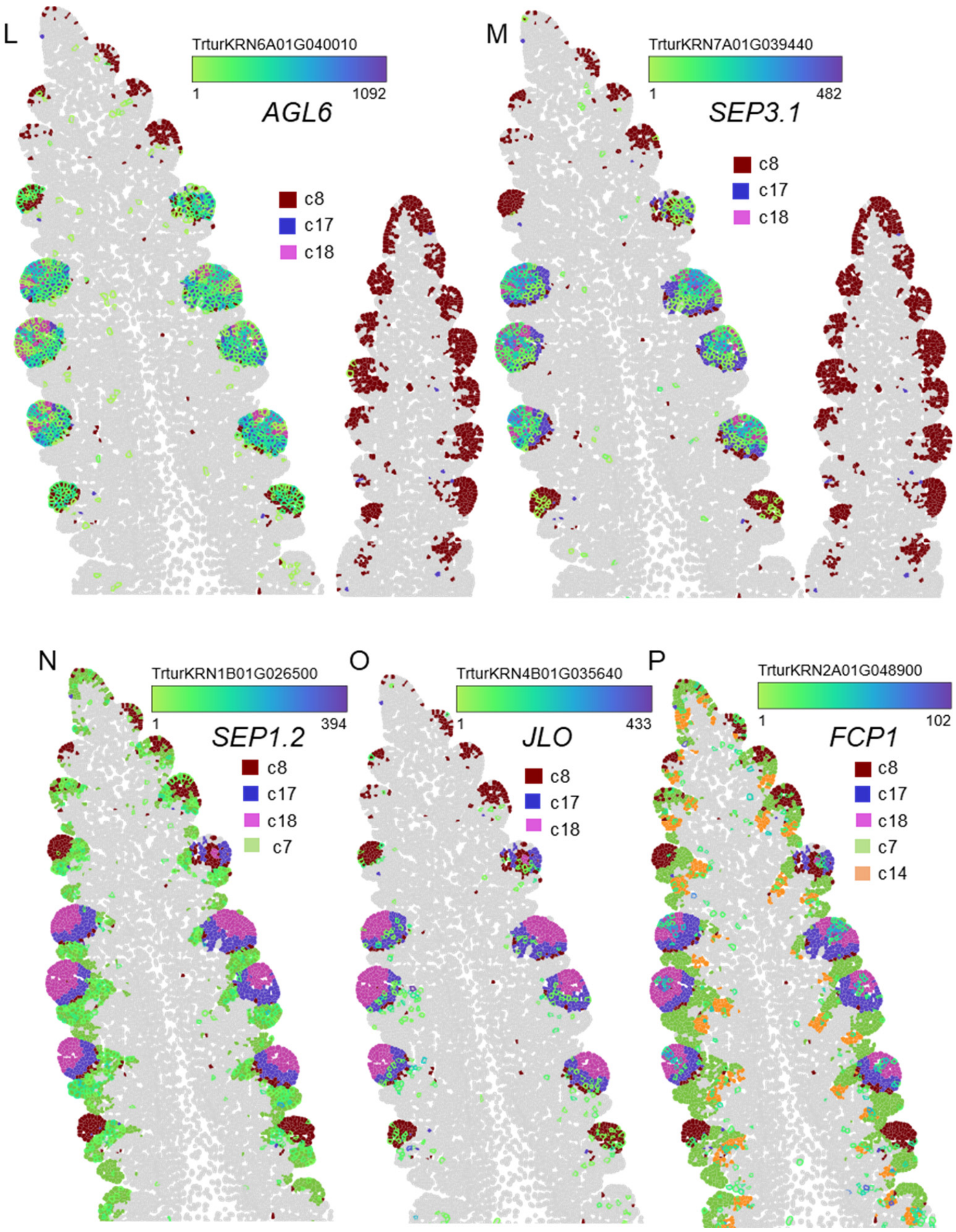

Additional file 1: Fig. S41. Genes co-expressed with *AGL-6*. Imputed expression of genes selected based on co-expression with *AGL6* and preferential expression in meristems. Only one homeolog is shown using the green-blue scale for the cell-border color. **A** *AG1* is included as control to show correct imputation of c18 location. **B** *DDM1a*, **C** *BRBN16*, and **D** *SHI1*. Annotation of the marker genes and references are available in Additional file 2: Table S1.

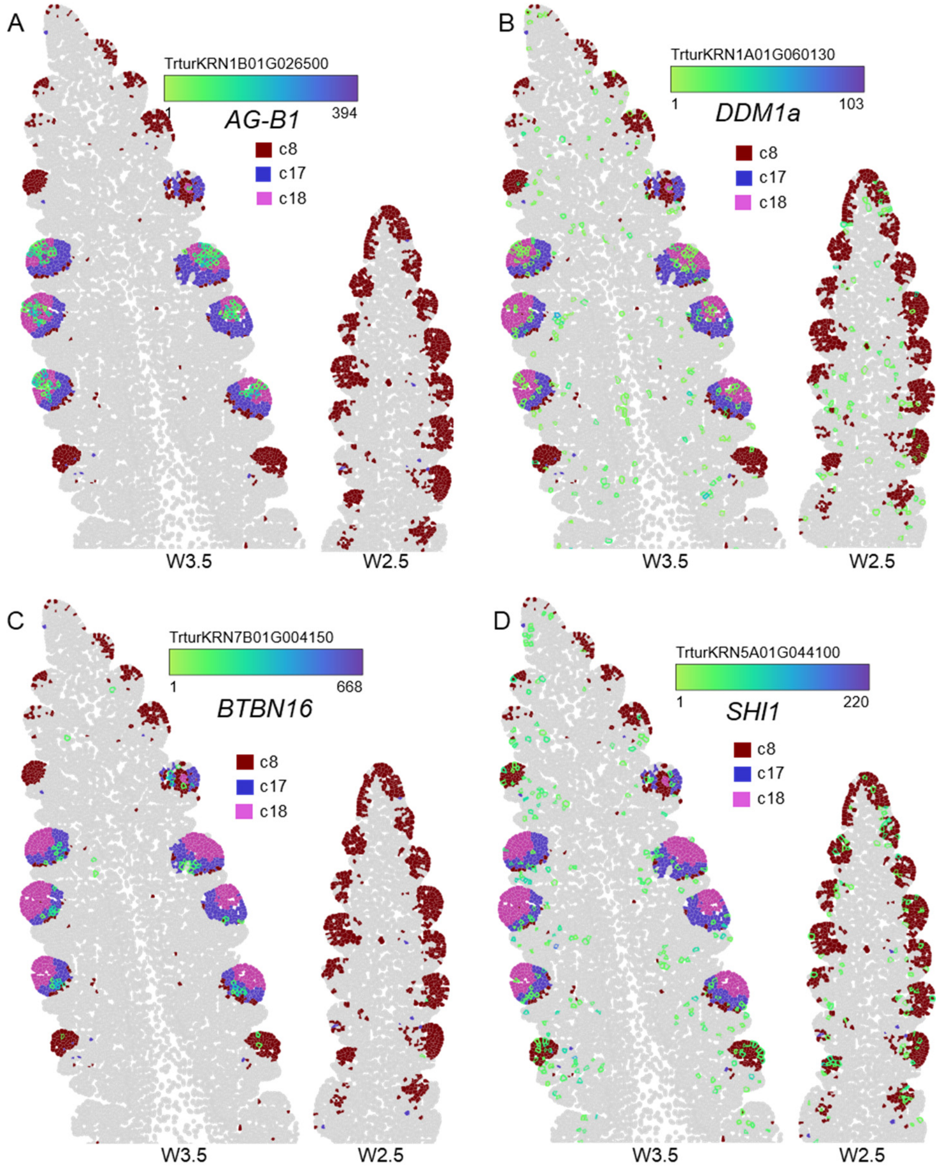

Additional file 1: Fig. S42. Gene network for *FZP* and *TCP24*. Gene regulatory network constructed with the program GENIE3. The network is based on the 988 genes co-expressed with *FZP* (Additional file 2: Table S20) and/or *TCP24* (Additional file 2: Table S21) in the scRNA-seq dataset. A summary of the gene interactions is presented in Additional file 2: Table S22. The network presented below includes the highly significant interactions visualized with Cytoscape 3.10.3 (https://cytoscape.org/).

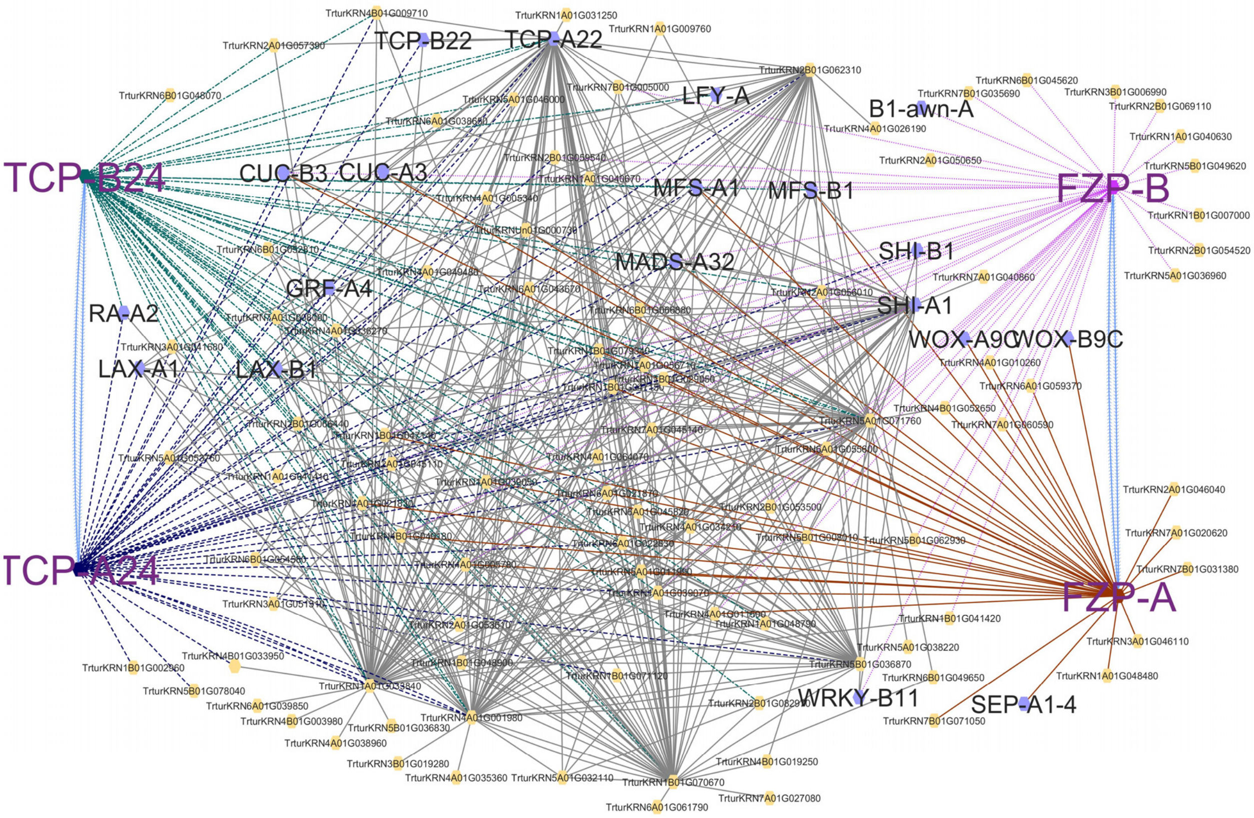

Additional file 1: Fig. S43. Heatmap for genes encoding ribosomal proteins co-expressed with *ULT-A1* (82 out of the 100 genes with highest correlations with *ULT-A1*). The *ULT-B1* homeolog showed limited co-expression with *ULT-A1* (*R*= 0.0398) and no enrichment in genes encoding ribosomal proteins suggesting functional differentiation between homeologs.

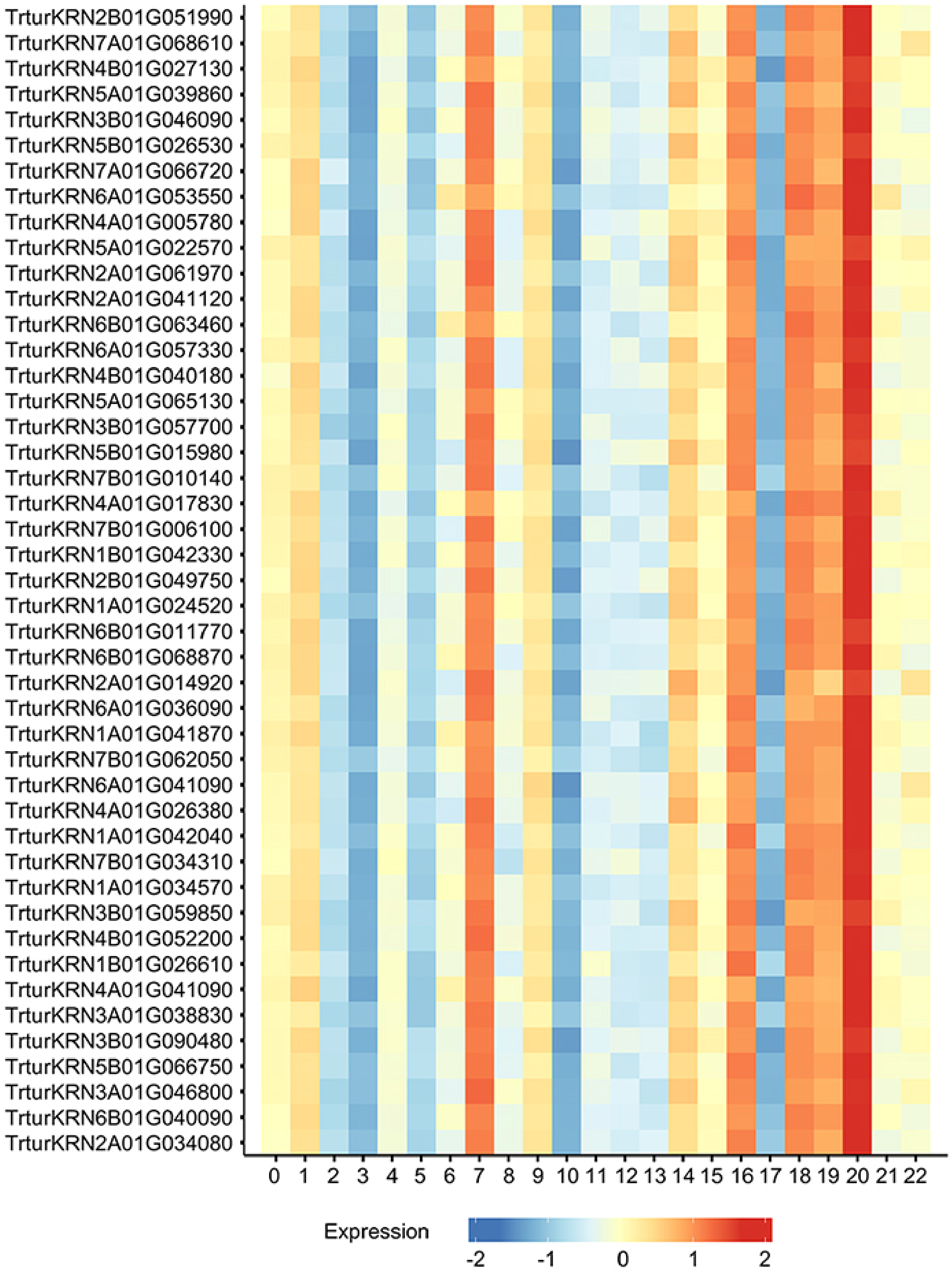

Additional file 1: Fig. S44. Trajectory analysis scRNA-seq clusters. The 23 cell clusters from the scRNA-seq analysis were used for a trajectory analysis generated with program Monacle 3. The cluster numbers and colors are the same as in Fig. 5. Cluster sc14, which was annotated as meristematic cells was used as root.

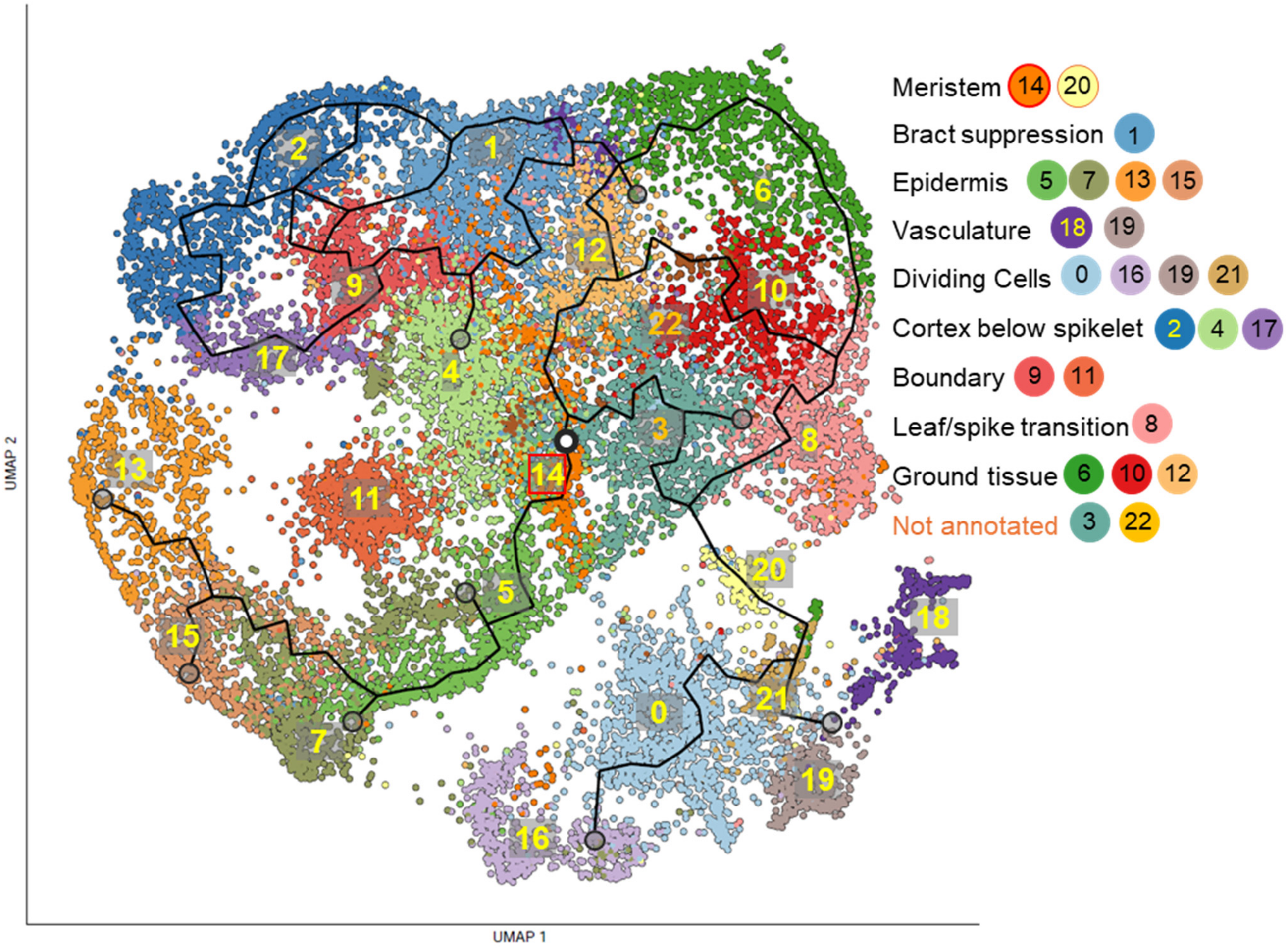

Supplement: Supplementary file 1 — Additional file 1: Fig. S1. Microscopy images of Kronos developing spikes. Fig. S2. Additional sections showing cell segmentation and clustering. Fig. S3. Calcofluor images of cell segmentation sections presented in Fig. 1. Fig. S4. Calcofluor images of cell segmentation sections in Additional file 1: Fig. S2. Fig. S5. Individual cell clusters identified at three spike development stages. Fig. S6. Validation of smFISH profiles by in situ hybridization. Fig. S7. Validation of smFISH profiles by MERFISH in hexaploid wheat. Fig. S8. Basal region below the spike smFISH. Fig. S9. Functional validation of LFY role in the intercalary meristem. Fig. S10. Expression of genes involved in the gibberellin pathway. Fig. S11. Differentially expressed genes in the transition zone. Fig. S12. Spike central region with preferentially expressed genes. Fig. S13. Genes preferentially expressed in vasculature cell clusters sc4 and sc13 at W3.5. Fig. S14. Genes preferentially expressed at the suppressed bract region. Fig. S15. Genes preferentially expressed at the base of the spikelets. Fig. S16. Genes preferentially expressed in cluster c14 at W3.5. Fig. S17. Functional validation of FZP. Fig. S18. Genes preferentially expressed in the spikelet meristem. Fig. S19. SPL14 and FZP expression in the inflorescence meristem. Fig. S20. Genes with different expression between W2.5 and W3.5 in the IM region. Fig. S21. Functional validation of SPL14. Fig. S22. smFISH clusters trajectory analysis. Fig. S23. Comparison between initial and final scRNA-seq clustering. Fig. S24. RNA-seq for three different sections of the spike. Fig. S25. Cell cycle scRNA-seq clusters. Fig. S26. Known markers for single-cell epidermal clusters. Fig. S27. Differential genes among scRNA-seq epidermal clusters. Fig. S28. Known vasculature markers. Fig. S29. Co-expression of APL phloem marker with MADS-box genes PI1 and AP3. Fig. S30. Genes expressed in the central spike photosynthetic ground tissue. Fig. S31. Heat map for [file 13059_2025_3811_MOESM1_ESM.pdf]
